# Towards a quantitative description of excitonic couplings in photosynthetic pigment–protein complexes: quantum chemistry driven multiscale approaches†

**DOI:** 10.1039/d1cp03566e

**Published:** 2022-01-03

**Authors:** Christian Friedl, Dmitri G. Fedorov, Thomas Renger

**Affiliations:** Institut für Theoretische Physik, Johannes Kepler Universität Linz Altenberger Str. 69 4040 Linz Austria thomas.renger@jku.at; Research Center for Computational Design of Advanced Functional Materials (CD-FMat), National Institute of Advanced Industrial Science and Technology (AIST) Central 2 Umezono 1-1-1 Tsukuba 305-8568 Japan d.g.fedorov@aist.go.jp

## Abstract

A structure-based quantitative calculation of excitonic couplings between photosynthetic pigments has to describe the dynamical polarization of the protein/solvent environment of the pigments, giving rise to reaction field and screening effects. Here, this challenging problem is approached by combining the fragment molecular orbital (FMO) method with the polarizable continuum model (PCM). The method is applied to compute excitonic couplings between chlorophyll *a* (Chl *a*) pigments of the water-soluble chlorophyll-binding protein (WSCP). By calibrating the vacuum dipole strength of the 0–0 transition of the Chl *a* chromophores according to experimental data, an excellent agreement between calculated and experimental linear absorption and circular dichroism spectra of WSCP is obtained. The effect of the mutual polarization of the pigment ground states is calculated to be very small. The simple Poisson-Transition-charge-from-Electrostatic-potential (Poisson-TrEsp) method is found to accurately describe the screening part of the excitonic coupling, obtained with FMO/PCM. Taking into account that the reaction field effects of the latter method can be described by a scalar constant leads to an improvement of Poisson-TrEsp that is expected to provide the basis for simple and realistic calculations of optical spectra and energy transfer in photosynthetic light-harvesting complexes. In addition, we present an expression for the estimation of Huang–Rhys factors of high-frequency pigment vibrations from experimental fluorescence line-narrowing spectra that takes into account the redistribution of oscillator strength by the interpigment excitonic coupling. Application to WSCP results in corrected Huang–Rhys factors that are less than one third of the original values obtained by the standard electronic two-state analysis that neglects the above redistribution. These factors are important for the estimation of the dipole strength of the 0–0 transition of the chromophores and for the development of calculation schemes for the spectral density of the exciton-vibrational coupling.

## Abstract 2

The importance of reaction field and screening effects on the excitonic couplings is demonstrated, and from quantum-chemical calculations a single scaling factor is derived that can be used to improve simple models based on the Poisson equation.

## Graphical abstract

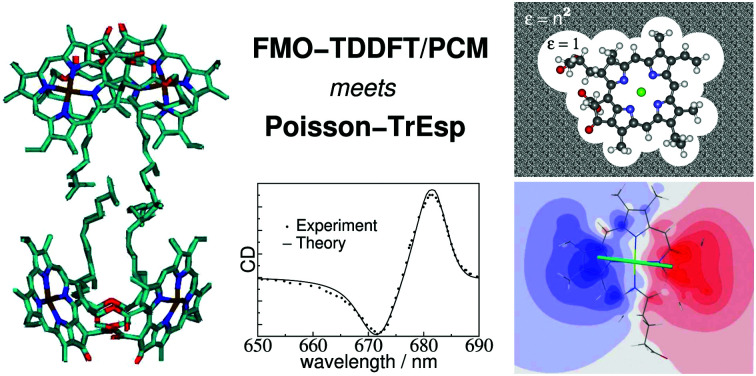

## 1. Introduction

Charge and energy transfer in materials is a broad field of research, including the transfer of excitation energy in systems with multiple chromophores.^1^ Förster resonance energy transfer (FRET)^2^ and related transfer mechanisms^3,4^ are very important phenomena in spectroscopy for measuring distances in fluorescent-labeled biomolecules,^5,6^ as well as for light harvesting in nano particles^7^ including organic solar cells^8^ and in photosynthesis with chlorophylls, carotenoids and related molecules serving as chromophores.^3,9^

For a molecular system composed of multiple chromophores, one can calculate excited states, *e.g.*, using multireference configuration interaction (MRCI)^10^ or time-dependent density functional theory (TDDFT).^11^ However, the cost of such calculations scales steeply with the system size. An alternative to this brute force approach is to compute individual chromophores at a high level and the interactions between them using a simplified model.^12,13^ Chromophores can be treated as fragments in fragment-based approaches,^14–22^ for some of which the excitonic coupling^23,24^ and delocalized excitations^25^ can be calculated. The excitonic couplings are responsible for energy transfer, and the delocalization of excited states results in a shift of optical transition energies and a redistribution of oscillator strength measured in optical spectroscopy on molecular aggregates.

The fragment molecular orbital method (FMO)^26–29^ at the TDDFT level^30,31^ has been interfaced with FRET in vacuum.^32^ Alternatively, there is an FMO method based on CI with single excitations, for computing excitonic couplings^33–35^ in vacuum, and a Green's function approach.^36^ In this work, FMO-based TDDFT and Hartree–Fock and configuration interaction with singles (HF/CIS) are combined with FRET and the polarizable continuum model (PCM)^37^ so that effects of the protein/solvent environment on the excitonic couplings^38,39^ can be studied. PCM has been applied to the calculation of excitonic couplings.^38–40^ In PCM calculations the polarization of the electronic ground state of the chromophores by the environment is taken into account by using a continuum description of the latter. In the present application, in addition, the mutual polarization of electronic ground states of all chromophores is studied, and we investigate how the regular PCM approach can be calibrated and used to improve other methods.

The excitonic couplings obtained with the quantum-mechanical (QM) FMO/PCM method in protein/solvent environment, developed in this work, are compared to two simpler models: the point-dipole approximation (PDA) and the Poisson-Transition-charges-from-Electrostatic-potential (Poisson-TrEsp) method.^41–43^ It is shown how screening and reaction field effects caused by the dynamical polarization of the protein/solvent environment influence the effective transition dipole moment of the chromophores and their excitonic couplings. While the Poisson-TrEsp method only takes into account the screening of the Coulomb coupling between non-polarizable transition densities of the chromophores, the present FMO/PCM method includes the polarization of the pigments by the reaction field of the protein/solvent environment in a self-consistent way. One aim of the present work is to find out whether this polarization effect leads to qualitative changes of the transition density of the chromophores. Our working hypothesis is that there are no strong qualitative changes and that the polarization effect, together with a correction for limitations of the quantum chemical methods, can be included in a simple calibration factor in an improved Poisson-TrEsp method.

**Fig. 1:**
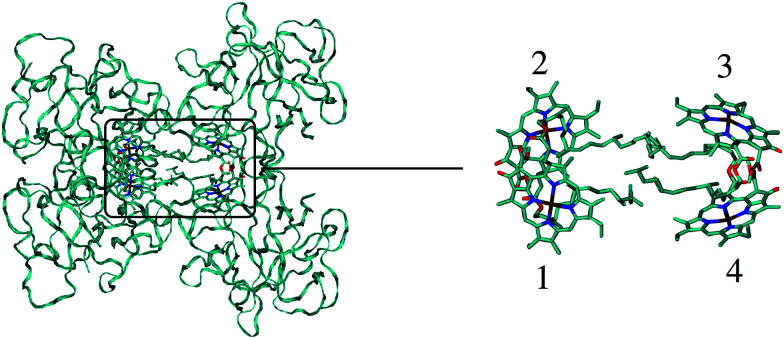
Structure of the WSCP complex from *Lepidium virginicum*.^87^ The left part shows the whole pigment–protein complex with the protein in ribbon style and the right part contains an enlarged view on the four central Chl *a* pigments. The whole complex has an approximate 222 symmetry that is disrupted only by the outer loop of the protein tetramer and the phytyl tails of the Chls forming a hydrophobic knot in the center of the complex.^87^

The different methods for the calculation of the excitonic coupling are applied in the calculation of linear absorption and circular dichroism spectra of the water-soluble chlorophyll-binding protein (WSCP). The tetrameric WSCP binds 4 chlorophyll *a* (Chl *a*) molecules, which are arranged in two dimers with weak inter-dimer and strong intra-dimer excitonic couplings. The phytyl chains of the pigments form a hydrophobic knot in the center of the complex, which keeps the tetramer together (Fig. 1). Due to weak inter-dimer couplings, the optical spectra of WSCP are determined by the excitonic dimers. The transition dipole moments of the two Chl *a* pigments in each dimer are arranged in an ‘open sandwich’ geometry. This geometry leads to a large oscillator strength of the upper exciton state and a small oscillator strength of the lower exciton state of the dimer.^44,45^

WSCP is not involved in photosynthetic light-harvesting. It is built up in plants when they experience drought, heat or salt stress.^46^ The exact functional role of WSCP has not been discovered yet.^47^ Due to its relatively simple structure, WSCP has been an important model system for the study of fundamental pigment–pigment and pigment–protein interactions.^48–59^ Because of the approximate *D*_2_ symmetry (Fig. 1), all four Chl *a* pigments have an equal average local excitation energy (site energy), and the excitonic coupling determines the splitting between exciton states that is seen in the spectra. Based on experimentally determined satellite holes in hole-burning spectra of Chl *a* – WSCP, an excitonic coupling of 100 cm^−1^ has been estimated in the dimers,^50^ that has been refined to 83 cm^−1^ from a fit of linear absorption and circular dichroism spectra.^59^ With this coupling, quantitative agreement with hole-burning data^50^ was obtained.^59^

WSCP is a rare example of a pigment–protein complex, where a direct experimental estimate of the excitonic coupling between 0–0 transitions of two strongly coupled Chl *a* chromophores is possible. There are four reasons that make WSCP a unique system for such an estimate and the development of new methods. (1) Due to its approximate *D*_2_ symmetric structure, the site energies of the Chl *a* chromophores are identical, as noted above. Hence, the splitting between optical lines is only determined by the excitonic coupling. (2) The chromophores are far enough apart such that electron exchange interaction can be neglected, greatly simplifying the theoretical analysis. (3) The high-energy exciton transition carries the major part of the oscillator strength of the 0–0 transitions of the chromophores in the dimers. This exciton transition can have a certain overlap with the vibrational sideband of the low-energy exciton transition. Therefore, a strong high-energy exciton transition is easier to identify in the spectrum. (4) The Franck–Condon factors involving excitations of the high-frequency intramolecular vibrational modes of Chl *a* are sufficiently small, so that this part of the vibronic coupling can be simply included by rescaling the excitonic coupling between the 0–0 transitions of the chromophores. Property (3) is consistent with the fact that WSCP is not a light-harvesting protein. A strong low-energy exciton transition would have advantages for energy transfer.

From hybrid quantum mechanics/molecular calculations that include a heterogeneous polarizability of the protein environment (QM/MMPol) with an induced dipole model, an excitonic coupling of 186 cm^−1^ was reported.^52^ At first glance, this coupling seems to overestimate the experimental value by more than a factor of two. Several reasons for this discrepancy, like an overestimation of the transition dipole moments by the quantum chemical method, were discussed.^52^ In order to relate this dipole strength to the experiment, the authors considered an analysis of the experimental dipole strength in different solvents by Knox,^60^ using a Lorentz local field factor and came to the conclusion that the excitonic coupling calibrated on these grounds would be too small (38 cm^−1^). Hence, up to now, there is no *ab initio* based explanation of the excitonic coupling value in the Chl *a* dimers of WSCP inferred from the fit of optical experiments. In the present work, we explain this value.

## 2. Methodology

### 2.1 Excitonic couplings from transition density

The basic approach to computing the important long-range part of excitonic couplings between chromophores is to calculate an excited state of interest in each chromophore and a transition density for an electronic excitation from the ground state to the excited state.^32^ The excitonic couplings are obtained from the Coulomb interaction between the transition densities of the chromophores. Other properties derived from the transition density are the transition dipole moment and the atomic transition charges. In contrast to the electron density of an electronic state, that has a very complex shape describing the electron distribution between atoms driven by their electronegativity, the transition density often has a simpler dipolar form. FMO provides a convenient framework for dealing with chromophores as fragments (one can also include non-chromophore fragments). By virtue of the availability of a covalent fragment boundary treatment the present formulation can be applied to bio^61^ and nano^62^ materials. To simplify the description, the following discussion assumes that chromophores are not split into multiple fragments.

The methodology for calculations in vacuum was developed earlier.^32^ In this work, the focus is on incorporating a PCM description of the protein/solvent environment of the pigments. We calculate chromophores with TDDFT, and the interaction of the chromophores with the protein/solvent environment is described in the framework of PCM. PCM is used to model the protein environment in the same way as the solvent. For excitonic couplings, the major contribution comes from the optical dielectric constant of the environment, which is very similar for proteins and aqueous solvents. Hence, the environment can be described by a homogeneous dielectric continuum surrounding the optically active pigments.

In FMO/PCM,^63^ a cavity is constructed around the whole molecular system containing all chromophores, and each fragment calculation is performed in this total cavity. On the cavity surface, divided into *N*_TS_ small pieces, called tesserae, point charges are placed, which represent the effect of the polarization of the environment by the chromophore. These charges are determined self-consistently with respect to the electronic ground state of the chromophore (that is, taking into account the mutual polarization of the environment and the chromophore). For this treatment, the response of the protein/solvent environment should be in equilibrium. This response is determined by the static dielectric constant *ε*_s_, which is much smaller for the protein than for the aqueous solvent. Because chromophores are surrounded by the protein, we use only the protein dielectric constants to describe the environment of the chromophores, indicated by the term “protein/solvent environment”. Note that the excitonic couplings for the present system essentially do not depend on *ε*_s_, as shown below.

Typically, the fragments in FMO are calculated in an embedding potential generated by the other fragments, except that the lowest order of FMO, denoted as FMO0, uses no embedding. In this work, FMO0, previously introduced in vacuum,^64^ is extended to include a homogeneous dielectric. This dielectric is described by a static dielectric constant *ε*_s_ and an optical dielectric constant *ε* = *n*^2^ (*n* is the refractive index). Whereas *ε*_s_ is used in the calculation of the electronic ground state density, *ε* is used for the electronic transition density, reflecting the fact that the slow part of the dielectric environment has no time to react during an electronic transition. In the calculations we only distinguish two regions, the cavity of the chromophores (with *ε* = *ε*_s_ = 1) and the environment (with *ε* = 2 and *ε*_s_ = 4) that includes both the protein and the solvent.

The inclusion or neglect of the polarization of one chromophore by all others is indicated by *n* in FMO*n* (FMO1: included, FMO0 neglected). In FMO-based PCM, the whole molecular cavity is constructed and used in each fragment calculation. In PCM[0], the solvent charges are induced by each fragment separately. In PCM[1], the solvent charges are induced by all fragments together. FMO0 can be combined with both PCM schemes, but FMO1 can only be used with PCM[1]. There exist also higher order embedding schemes, *e.g.*, FMO2,^65^ which can include explicit higher many-body TDDFT corrections for a single chromophore. However, FMO2-TDDFT is difficult to apply to multiple chromophores,^66^ so FMO2 is not used in the present work.

The coupling matrix element between two excited states of the complex that are localized on chromophores (fragments) *M* and *N* is given as a sum of three contributions^67^

1 *V*_*MN*_ = *V*^ES^_*MN*_ + *V*^XC^_*MN*_ + *V*^CT^_*MN*_,

with the electrostatic (ES) coupling *V*^ES^_*MN*_ and the short-range exchange–correlation (XC) and charge-transfer (CT, related to the density overlap) couplings *V*^XC^_*MN*_ and *V*^CT^_*MN*_, respectively. The latter two are neglected in this work (it was shown^67^ that their values are small for an interfragment separation of 4 Å or more). Hence, we have *V*_*MN*_ = *V*^ES^_*MN*_.

**Fig. 2:**
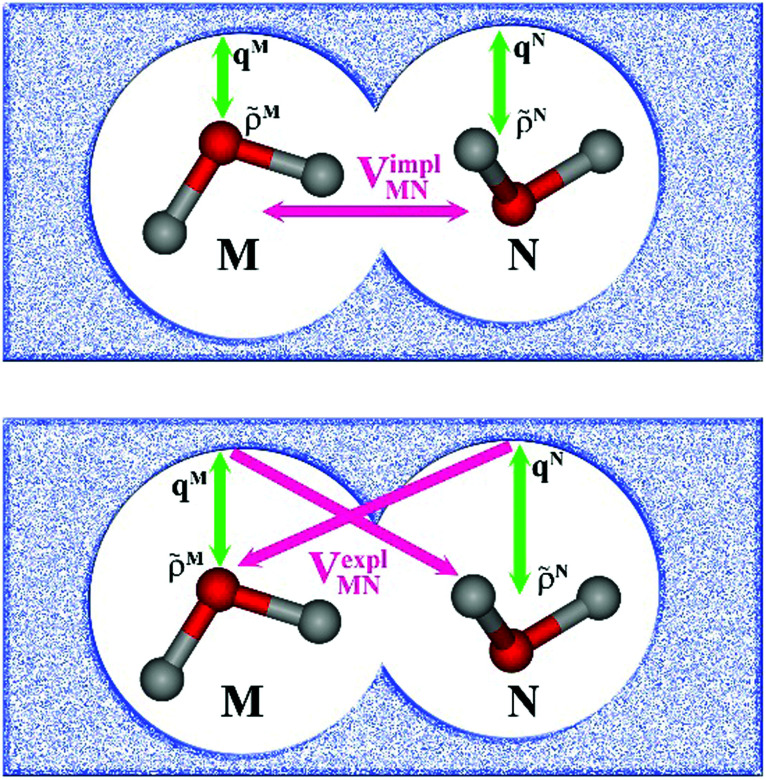
Schematic representation of two molecules *M* and *N* embedded in a protein/solvent environment, represented by the dotted pattern. Upper panel: Illustration of the reaction field effect. The transition densities of the two chromophores *ρ̃*^*M*^(**r**) and *ρ̃*^*N*^(**r**) dynamically polarize the environment. The polarized environment (described by induced transition surface charges *q*^*M*^_*i*_ and *q*^*N*^_*i*_) dynamically polarizes the chromophores *via* their transition densities, resulting in the implicit environmental contribution *V*^impl^_*MN*_ to the excitonic coupling (schematically *ρ̃*^*M*^·*ρ̃*^*N*^). Lower panel: Illustration of the screening effect. The surface transition charges of the environment induced by the transition densities of one chromophore interact with the transition density of the other chromophore, giving rise to the explicit environmental contribution *V*^expl^_*MN*_ to the excitonic coupling (schematically *ρ̃*^*M*^·*q*^*N*^ or *ρ̃*^*N*^·*q*^*M*^). Green arrows represent the mutual polarizing potentials, pink arrows represent the interaction (that is, the excitonic coupling). Note that transition charges *q*^*M*^ are induced by chromophore *M* on the whole surface, not just in the vicinity of *M* (likewise, for *N*).

In an environment described by the PCM,^38–40^ the electrostatic coupling *V*^ES^_*MN*_ between the transition densities of the pigments contains implicit and explicit environmental contributions, *V*^ES^_*MN*_ = *V*^impl^_*MN*_ + *V*^expl^_*MN*_ (Fig. 2). The implicit (impl) contribution *V*^impl^_*MN*_ describes the effect of the dynamic polarization of the transition density of the chromophores by the reaction field of the environment. The explicit (expl) environmental contribution *V*^expl^_*MN*_ contains the screening effects. It is the electrostatic interaction between the transition density of one pigment with the dynamic polarization of the protein/solvent environment (represented by surface charges) induced by the transition density of another pigment, very much like the typical solute–solvent screening.^68,69^

The transition densities of the two chromophores that enter *V*^impl^_*MN*_ and *V*^expl^_*MN*_ are determined by quantum chemical calculations on the chromophore monomers embedded in a dielectric continuum representing the protein/solvent environment. Due to mutual dynamic polarization of the chromophore and the environment, the transition dipole moment of the chromophore is enhanced. A calculation scheme for the electrostatic coupling in vacuum *V*^ES^_*MN*_(*ε* = 1) between transition densities of the chromophores in the framework of the FMO methodology was developed before.^32^ It can be used to obtain the implicit contribution *V*^impl^_*MN*_ by replacing the vacuum transition densities of the solutes by the transition densities obtained in the protein/solvent environment, described by PCM.

The explicit protein/solvent contribution *V*^expl^_*MN*_, arising from the coupling of the dynamic protein/solvent polarization, induced by the transition density of one chromophore with the transition density of the other chromophore, is obtained by perturbation theory in the sense that the dynamic protein/solvent polarization on one chromophore is not affected by the coupling to the transition density of the other chromophore. The explicit term can be computed as2
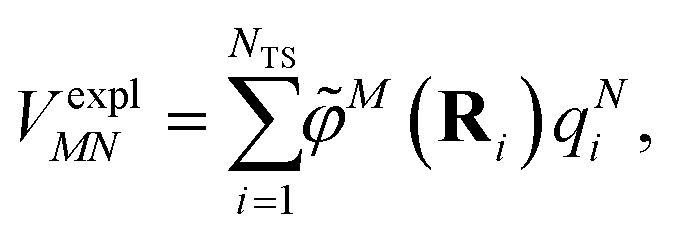
where *q*^*N*^_*i*_ are the surface transition charges representing the dynamic polarization of the protein/solvent environment induced by the transition density of chromophore *N*. The charge placed at the center of a surface element (tessera) *i* with coordinates **R**_*i*_ interacts with the transition density *ρ̃*^*M*^(**r**) of chromophore *M*, an interaction that is described by the ESP *φ̃*^*M*^(**r** = **R**_*i*_) of chromophore *M* acting at the position **R**_*i*_, where3
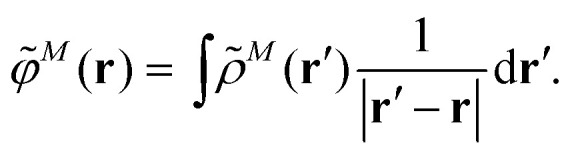


The electrostatic interaction *V*^expl^_*MN*_ in eqn (2) should be symmetric with respect to the chromophore indices *M* and *N*, *V*^expl^_*MN*_ = *V*^expl^_*NM*_. In order to avoid a violation of this symmetry by the numerical artifacts in PCM, we enforce the symmetry by using the following expression4
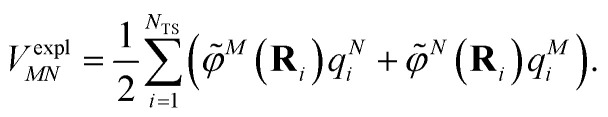
Note that the above equation for the explicit protein contribution is conceptually identical to the partial screening model of pair interactions used in FMO/PCM.^68^ However, for the screening of the excitonic coupling in this work, the transition density *ρ̃*^*M*^ is used in eqn (3), and there is no nuclear contribution to *φ̃*^*M*^(**r**).

The components *a* of the transition dipole moment of a chromophore *M* for *a* = *x*, *y* or *z* can be evaluated as5
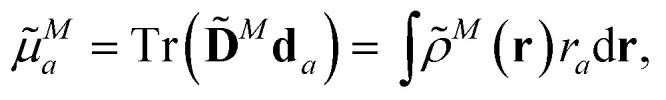
where **d**_*a*_ is the dipole integral matrix and **D̃**^*N*^ is the transition density matrix for chromophore *M*. Both matrices are in the atomic orbital basis.

An alternative description of the explicit protein contribution *V*^expl^_*MN*_ in eqn (2) is given by6
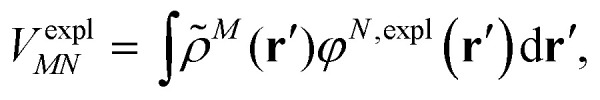
where we introduced the explicit ESP^70^*φ*^*N*,expl^(***r***) of the dynamic environmental polarization7
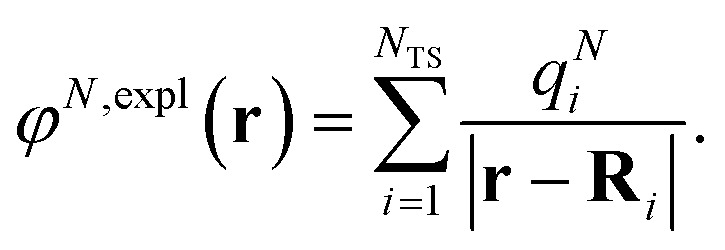


The surface charges *q*^*N*^_*i*_ that represent the dynamic polarization of the protein/solvent environment, induced by the transition density of chromophore *N*, can be used to define an explicit dipole moment of the dynamic environmental polarization8
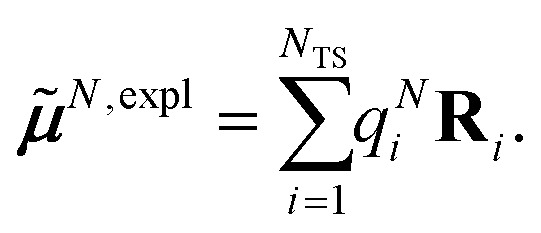


This dipole moment is used below to quantify the screening of the interaction between the transition dipole moments of the chromophores, within the point-dipole approximation (PDA) of the excitonic coupling. While absolute values of couplings strongly depend on the distance between pigments (for interpigment distances, see Table S1, ESI†), the ratios introduced below are much more uniform. The reaction field (rf) factor9
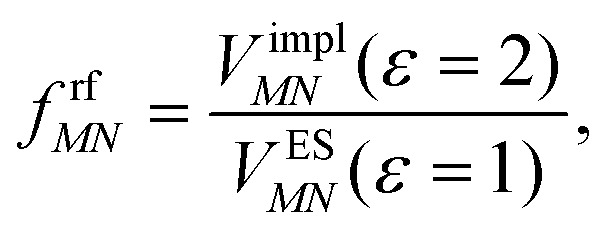
relates the unscreened coupling in vacuum and in solution. Hence, for *f*^rf^_*MN*_ only the implicit effect of the solvent is taken into account.^67^

Here, *ε* = 1 corresponds to vacuum and *ε* = 2 is the optical dielectric constant of the protein/solvent embedding (more details on the choice of *ε* are given in Section 3). The screening factor is defined as the ratio between the total electrostatic coupling and the implicit contribution^38–40^10
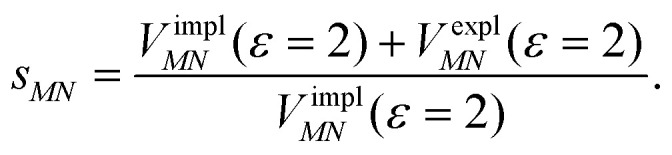
These two factors are introduced to discuss the implicit and the explicit effects of the protein/solvent environment.

### 2.2. Excitonic couplings in the point-dipole approximation (PDA)

The implicit contribution to the Coulomb coupling between the transition densities of chromophores (fragments) *M* and *N* in the PDA is obtained as^32^11
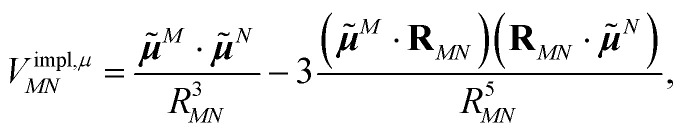
using the transition dipole moments ***μ̃***^*M*^ and ***μ̃***^*N*^ of chromophores *M* and *N*, respectively, and the vector **R**_*MN*_ = **R**_*M*_ − **R**_*N*_ that connects the centers of the two chromophores. Note that, the dipole moments include the implicit environmental effects *via* mutual dynamic chromophore-environment polarization.

The explicit protein contribution (screening) in PDA is evaluated as12
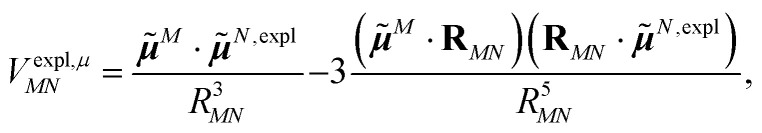
where ***μ̃***^*N*,expl^ (eqn (8)) is the dipole moment of the dynamic environmental polarization induced by the transition density of chromophore *N*.

Adding eqn (11) and (12), one obtains the total excitonic coupling in PDA as13
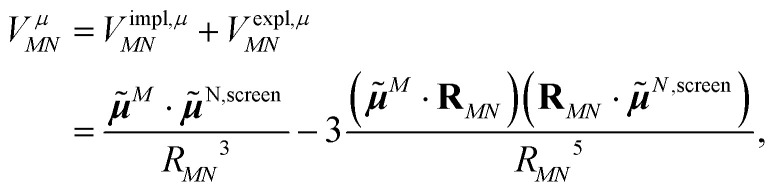
where the screened transition dipole moment ***μ̃***^*N*,screen^ = ***μ̃***^*N*^ + ***μ̃***^*N*,expl^ contains implicit and explicit contributions of the environment.

### 2.3. Excitonic coupling with the Poisson-TrEsp method

In the Poisson-TrEsp method,^41–43^ atomic transition charges are introduced to approximate the electrostatic potential of the transition density. These transition charges are placed in a molecule-shaped cavity. An important difference to PCM, as discussed above, is the neglect of the polarization of the chromophores by the protein/solvent environment. The transition charges of the chromophores in the original Poisson-TrEsp method are rescaled based on the vacuum transition dipole moment that was extracted by Knox from an analysis of the dipole strength of Chl *a* in different solvents,^60^ using an empty spherical cavity model, discussed in more detail in Section 2.4.

In Poisson-TrEsp, perturbation theory is used to describe the screening effects. This perturbation theory can be translated into classical electrostatics.^43^ The Poisson equation is solved for the electrostatic potential *φ̃*^*M*^(**r**) of pigment *M* using its atomic transition charges *q̃*^*M*^_*α*_ obtained from the fit of the ESP of the transition density14
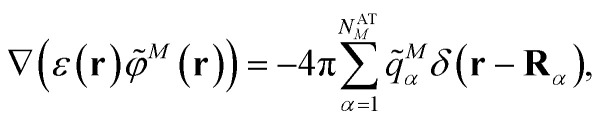
where the optical dielectric constant *ε*(**r**) is equal to 1 if **r** is inside the molecular cavity and 2 otherwise; **R**_*α*_ is the vector of coordinates of atom *α*. Note that, the tilde on the top of the atomic transition charges *q̃*^*M*^_*α*_ is used to distinguish them from the surface transition charges *q*^*N*^_*i*_ representing the dynamic polarization of the protein/solvent environment, introduced above (eqn (2)). The excitonic coupling between pigments *M* and *N* is obtained as15
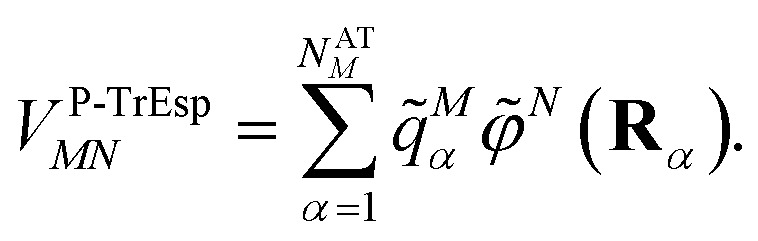
If *ε*_opt_(**r**) = 1 everywhere (no dielectric, *i.e.*, vacuum), then the coupling is16
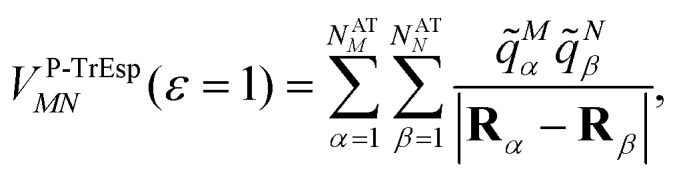
which is also known as the TrEsp coupling.^71^

For the Poisson-TrEsp method, we define the screening factor as17
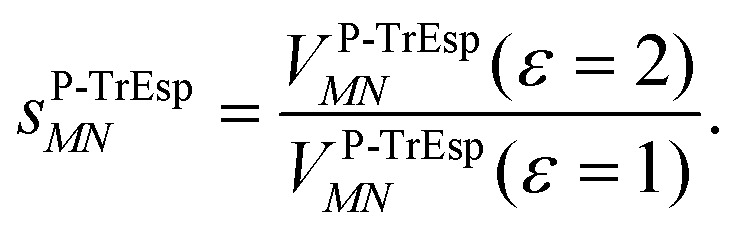


In this work, we want to investigate how this factor relates to the screening factor of PCM (eqn (10)) and how the reaction field effects, neglected in previous publications, can be incorporated in the Poisson-TrEsp method. In a first step, we study the dependence of the dipole strength of the chromophores on the optical dielectric constant *ε* of the protein/solvent environment, how it is described in the empty spherical cavity model and which role reaction field effects play.

### 2.4. Dependence of dipole strength of chromophores on the optical dielectric constant *ε*

In the empty spherical cavity model, the transition density of the chromophore is described by a non-polarizable point-dipole that is located in the center of a spherical cavity, which is surrounded by a homogeneous medium with dielectric constant *ε*. Inside the cavity we have *ε* = 1 (vacuum). In such a cavity an external field is enhanced by a factor 3*ε*/(2*ε* + 1),^72^ caused by the polarization effects of the dielectric by the external field. Only the optical dielectric constant is relevant, because the slow part of the polarization cannot follow the oscillations of the light field. Since the intensity for the absorption of light is proportional to the square of the scalar product between the field and the transition dipole moment, the enhanced field inside the cavity can be implicitly treated by an increased dipole strength of the chromophore

18 *D*(*n*) = *f*(*n*)*D*_0_.

Where the vacuum dipole strength is *D*_0_ = |***μ̃***_0_|^2^ with the vacuum transition dipole moment ***μ̃***_0_. Hence, the enhancement cavity field factor *f*, for such a spherical cavity is (note that *ε* = *n*^2^)19
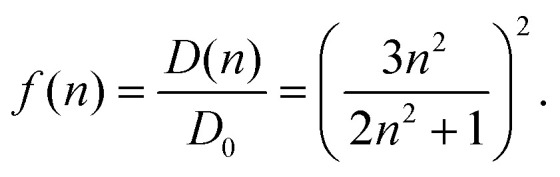


**Fig. 3:**
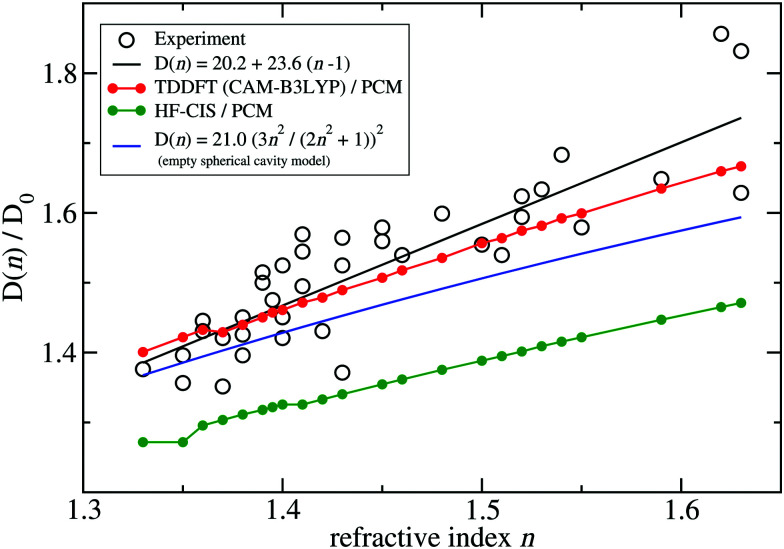
Ratio of the dipole strength *D*(*n*) of chlorophyll *a* for a given refractive index *n* of the solvent and the vacuum value *D*_0_ = *D*(*n* = 1). The experimental data^60^ (open circles) are compared with prediction of the empty spherical cavity model (blue line),^60^ an empirical linear fit of the data^60^ (black line) and the ratio of the square of the respective transition dipole moments, obtained with PCM calculations, using either TDDFT with the CAM-B3LYP functional (red line) or HF-CIS (green line) quantum chemical calculations on the 4 Chl *a* chromophores of WSCP (for contributions of individual Chls see ESI,† Fig. S5–S8 and S9–S12). The *D*_0_ value of the experimental data (20.2 D^2^) has been obtained by a fit of the experimental dipole strength *D*(*n*) by the empirical relation, given in the figure legend.

A fit of the experimental dipole strengths with this model (the blue line in Fig. 3) results in a vacuum dipole strength of 21.0 D^2^.^60^ However, the real molecular cavity is not spherical, and the transition density of the chromophore can be polarized by the solvent.

To investigate the latter effect using a realistic shape of the molecular cavity, we study the dependence of the transition dipole moment of Chl *a* in solution on the optical dielectric constant *ε* with TDDFT/PCM and HF/CIS/PCM calculations by varying *ε*. Note that in these calculations also the static dielectric constant *ε*_s_ enters, because the electronic ground state of the chromophore is polarized by the protein/solvent environment, before the optical excitation occurs. However, by doing preliminary calculations, it was found that the dependence on the static dielectric constant is weak (Tables S6–S11, ESI†) and, hence, the same static dielectric constant *ε*_s_ = 4 is used for every data point in Fig. 3. The transition dipole moment of the chromophore is enhanced by the reaction field effects that occur because of the mutual dynamic polarization of the chromophore and the protein/solvent environment. In this case, the enhancement factor of the dipole strength is obtained as

20 *f*(*n*) = |***μ̃***(*ε*)|^2^/|***μ̃***(*ε* = 1)|^2^,

where ***μ̃***(*ε*) is the transition dipole moment, calculated for a given optical dielectric constant *ε*. The different enhancement factors *f*(*n*) are compared in Fig. 3 with the experimental data, where for the latter *D*_0_ = 20.2 D^2^ was obtained from the empirical relation^60^

21 *D*(*n*) = 20.2 + 23.6(*n* − 1).

TDDFT(CAM-B3LYP)/PCM (red curve, Fig. 3) or HF/CIS/PCM (green curve), properly treat the reaction field effects for a realistic molecular cavity and show qualitative agreement with experiment, in particular for the former, even though the cavity field effect is neglected. The reaction field factors of the two methods (TDDFT and HF/CIS) for 
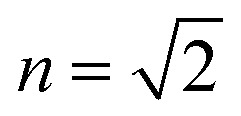
, relevant for the protein environment, differ by about 10%.

Do the neglected cavity field effects have an effect on the excitonic coupling and/or the optical spectra? Since the matrix element for the excitonic coupling does not depend on the external field, for the couplings, there is no effect. On the other hand, the optical spectra are measured with an external field and, hence, include cavity field effects. However, for a molecular aggregate made of identical chromophores, these effects have no influence on the shape of the spectrum. The equal shape of the subcavities of the chromophores leads to an equal enhancement of the electromagnetic field. Therefore, the peak heights in the spectrum, related to the square of the scalar product between transition dipole moments and the field, are affected by the cavity field effect identically for each peak. Hence, adding the cavity field effect would simply scale the total spectrum. In principle, the PCM calculations could be extended to include the cavity field effect.^73^ Such an extension could be useful in order to provide a more quantitative description of the experimental dipole strengths, and on this basis evaluate the reaction field effects obtained with different quantum chemical methods.

Although at present there is still an uncertainty concerning the exact magnitude of the reaction field factor, reflected by the 10% variation obtained between TDDFT/CAM-B3LYP and HF/CIS calculations, we can already conclude that the agreement of the empty spherical cavity model (the blue line in Fig. 3) with the experimental data is fortuitous, attributed to error compensation effects between using a spherical cavity and neglecting reaction field effects.

### 2.5 Delocalized excited states and optical spectra

To obtain the excitation energies of the low-energy delocalized states, one diagonalizes the exciton Hamiltonian22
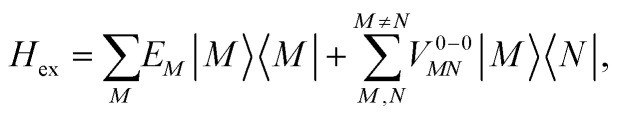
containing the local excitation energies (site energies) *E*_*M*_ of the chromophores (fragments) in the diagonal (*i.e.*, 〈*M*|*H*_ex_|*M*〉 = *E*_*M*_), and the excitonic couplings *V*^0–0^_*MN*_ between the intramolecular 0–0 transitions of the chromophores (in the off-diagonal 〈*M*|*H*_ex_|*N*〉 = *V*^0–0^_*MN*_). This coupling determines the splitting between low-energy exciton states of the complex in the presence of the coupling to intramolecular vibrations, as discussed in detail below (Section 2.6). Here, “0–0” refers to an electronic transition between the electronic ground and excited states with zero excited intramolecular vibrational quanta in both states. The site energies *E*_*M*_ refer to the energy of the 0–0 transition from the g0 (ground electronic, ground intramolecular vibrational state) to the e0 state (excited electronic, ground intramolecular vibrational), but we omit the superscript “0–0” for simplicity.

In eqn (22), we adopt the 
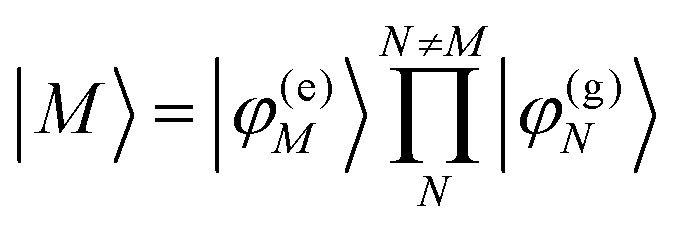
 Hartree ansatz for a localized excited state of the complex, in which chromophore *M* is excited and the remaining chromophores *N* ≠ *M* are in their electronic ground state, where |*φ*^(e)^_*M*_〉 and |*φ*^(g)^_*N*_〉 are the electronic excited and ground state wave functions. Note that the local excited states of the complex are orthogonal, 〈*M*|*N*〉 = *δ*_*MN*_, because the ground- and excited state wave functions of a chromophore *M* are orthogonal, that is, 〈*φ*^(e)^_*M*_|*φ*^(g)^_*M*_〉 = 0. A non-negligible overlap between local chromophore states would render the Hartree product ansatz invalid. For WSCP this neglect is justified by the large interchromophore distances. A proper antisymmetrization of |*M*〉 would yield the third (“exchange”) and fourth (“overlap”) term in eqn (1).

After the diagonalization of the matrix of the *H*_ex_ operator in the basis of local excited states |*M*〉, one can rewrite eqn (22) as23
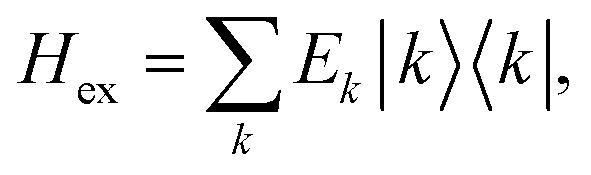
with the eigenenergies *E*_*k*_ and the eigenstates 
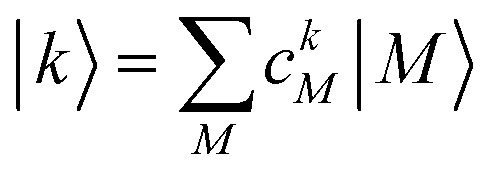
. The coefficient *c*^*k*^_*M*_ represents the *M*th component of the *k*th eigenvector of the exciton matrix (eqn (22)). The matrix size of *H*_ex_ is equal to the number of states coupled; if one state per chromophore is used, the matrix size is equal to the number of chromophores.

The Hamiltonian in eqn (22) is often applied to quasidegenerate chromophores, *i.e.*, when fragments are chemically the same, and their excitation energies are only slightly different due to their different local environments. The matrix in eqn (22) is usually constructed for a single excited state per chromophore, whereby one has to specify which excited state to pick, because the order of the excited states may depend on the chromophore, so that for example, the second excited state in chromophore *M* = 2 can correspond to the third excited state in chromophore *M* = 5. In complicated cases it may be necessary to analyze the nature of the excited states in detail and manually pick those that should be coupled. If an excited state is far separated in terms of *E*_*M*_ from all other states, then it will stay localized after the matrix diagonalization and, hence, does not need to be included in the exciton matrix. If needed, a perturbative inclusion of these off-resonant states is possible, as used, *e.g.*, in the description of non-conservative circular dichroism spectra of pigment–protein complexes.^74,75^ If two excited states in one monomer are close in energy and nearly resonant to an excited state in another monomer, all three states may be included in the exciton matrix. In the present application to a Chl *a* dimer of WSCP an inclusion of the first excited state in each chromophore is enough to analyze the low-energy region of the optical spectra. Note, that the current implementation of FMO-FRET supports just one excited state per fragment.

The linear absorption spectrum of the complex is obtained as24
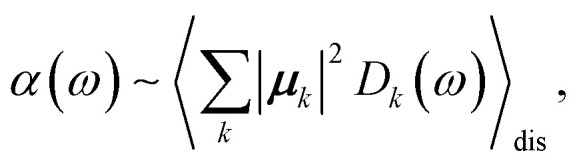
where25
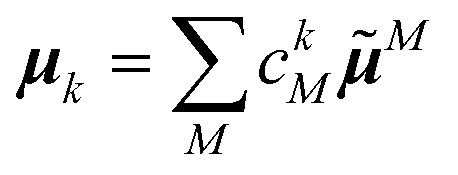
is the transition dipole moment of the *k*th exciton state. In the circular dichroism spectrum26
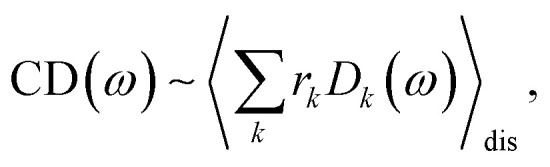
the |***μ***_*k*_|^2^ of the absorption spectrum (eqn (24)) is replaced by the rotational strength27
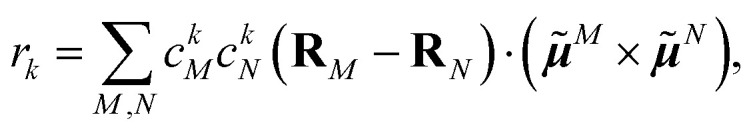
containing the centers of chromophores *M* and *N*, **R**_*M*_ and **R**_*N*_, respectively, that are defined in detail further below.

The conformational dynamics of the protein leads to fluctuations of the exciton Hamiltonian (eqn (22)), that is, the site energies and the excitonic couplings become time-dependent. The fluctuations that are fast compared to the excited state lifetimes of the pigments are taken into account in the homogeneous lineshape function *D*_*k*_(*ω*) and the slow fluctuations are described by the disorder average 〈*O*(*ω*)〉_dis_ of the intensity *O*(*ω*) of a homogeneous spectrum.

The fast fluctuations give rise to exciton relaxation-induced lifetime broadening and vibrational sidebands in the line-shape function *D*_*k*_(*ω*). A time-local density matrix theory expression is used for the lineshape function, as described earlier.^59,76^ This lineshape function includes the low-frequency continuous intermolecular part of the spectral density of the exciton–vibrational coupling. Note that in the present lineshape function uncorrelated diagonal disorder is considered, that is, we neglect a fluctuation of the excitonic couplings and the correlation between fluctuations of local excitation energies (site energies) of different pigments. A microscopic justification for this assumption was obtained in a normal mode analysis of the spectral density.^77,78^ The discrete high-frequency intramolecular part is not explicitly taken into account, since it contributes only at the high-frequency wing of the spectrum. The respective transitions are localized by static disorder, because small Franck–Condon factors lead to small effective excitonic couplings. Here, we concentrate on the main part of the spectrum that is dominated by delocalized 0–0 transitions. The high-frequency intramolecular vibrations are implicitly taken into account by a renormalization of the excitonic coupling as discussed in detail below (Section 2.6).

The disorder average of a spectral intensity *O*(*E*_1_, *E*_2_,…, *E*_*N*chr_, *ω*) (in this case the homogeneous absorption or circular dichroism), that depends on the local excitation energies *E*_*M*_, *M* = 1,…,*N*_chr_ of the *N*_chr_ chromophores, is defined as28
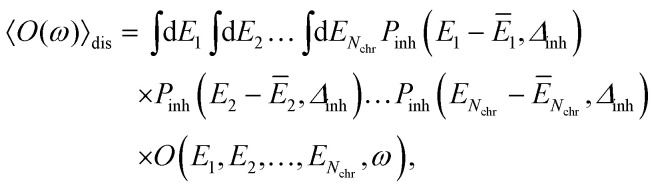
where *P*_inh_(*E*_*M*_ − *Ē*_*M*_, *Δ*_inh_) is an inhomogeneous (inh) Gaussian distribution function of a certain width *Δ*_inh_ centered at the mean site energy *Ē*_*M*_ of pigment *M*. Note that (fixed) site energies *E*_*M*_ in eqn (22) become variable in eqn (28). The variable site energies are used to describe fluctuations of the molecular structure that are slow compared to the excited state lifetimes. The high-dimensional integral in the disorder average is conveniently calculated with a Monte Carlo technique. Many different realizations of static disorder, *i.e.*, *E*_*M*_ values for every pigment in the complex are randomly drawn from the Gaussian distribution function *P*_inh_. For every such realization, the Hamiltonian in eqn (22) is diagonalized, assuming fixed values for the excitonic couplings *V*_*MN*_. This diagonalization results in eigenenergies *E*_*k*_ (eqn (23)) and respective eigenvectors with elements *c*^*k*^_*M*_. The function *O* (the homogeneous absorption or circular dichroism spectrum), which depends on these quantities is calculated for many (10^6^) different realizations of static disorder and the average over the respective homogeneous spectra gives the inhomogeneous spectrum.

Note that, the choice of a Gaussian distribution function can be motivated by the central limit theorem of statististics and was recently justified by structure-based simulations for a pigment–protein complex,^79^ where it was also shown that the variation in excitonic couplings is much smaller than that of the site energies and that correlations in static disorder are very small. The quantum chemical calculations only need to be performed once for the geometry-optimized structure, revealing the local transition dipole moments of the chromophores and the excitonic couplings, which are assumed to be constant across the inhomogeneous ensemble of complexes. In the present application to Chl *a* dimers in WSCP, the maximum of the distribution function *Ē*_*M*_ of the site energies of the two pigments in the dimers are identical for symmetry reasons. This *Ē* = *Ē*_1_ = *Ē*_2_ is treated as a fit parameter, together with the width *Δ*_inh_ of the distribution function. A change in *Ē* essentially leads to a displacement of the whole spectrum along the energy axis. Hence, this parameter can be inferred easily from experimental data. Note that, the excitonic couplings between Chls in different dimers in WSCP are so small that they have practically no influence on the shape of the linear absorption and circular dichroism spectrum of the complex.

### 2.6. Coupling of electronic excitations with high-frequency intrachromophore vibrations

In order to understand the physical nature of the scaling factor that relates the total excitonic coupling *V*_*MN*_ to the coupling of the intramolecular 0–0 transitions of the chromophores *V*^0–0^_*MN*_, we sketch the framework for the vibronic coupling. A key quantity is the overall Huang–Rhys factor *S* of intramolecular modes of the chromophores. In Section 2.7 an expression is derived to estimate *S* from fluorescence spectra of the dimer.

In order to describe the coupling of the electronic and vibrational degrees of freedom, we extend the monomer basis in eqn (22) to be a product of the electronic and vibrational wave functions. Each chromophore is described by an electronic ground (g) state 
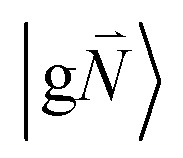
 and an excited (e) state 
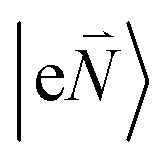
, where the vector 
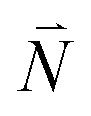
 contains the vibrational quantum numbers *N*_*ν*_ of the different intramolecular modes *ν*.

Linear absorption starts from the ground state 
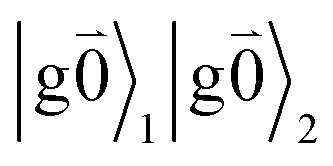
 with no vibrational excitation in both monomers (indicated by 
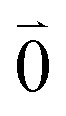
). Because of the excitonic coupling *V*_12_ between the chromophores their excited states get mixed. If the excitonic coupling between the 
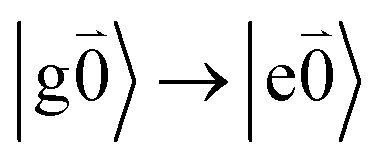
 transition (in short the 0–0 transition) of one chromophore and the 
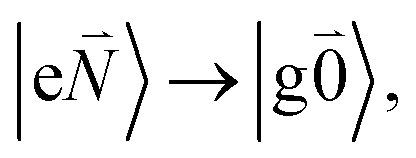
 transition (with 
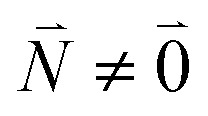
) of the other chromophore is small compared to the energy difference between the two transitions, the 0–0 transition of one chromophore mixes mainly with the 0–0 transition of the other chromophore and the two lowest excited states (*k* = 1, 2) of the dimer are in good approximation obtained as29
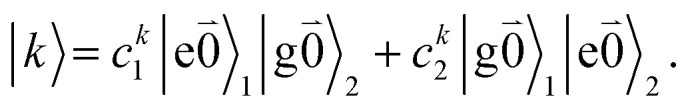
The exciton coefficients *c*^*k*^_1_ and *c*^*k*^_2_ are obtained by diagonalizing the exciton Hamiltonian in eqn (22) that contains in the diagonal the local 0–0 transition energies *E*_*M*_ of the two chromophores (which include the difference in zero point energies of the excited and ground states) and in the off-diagonal the excitonic coupling 
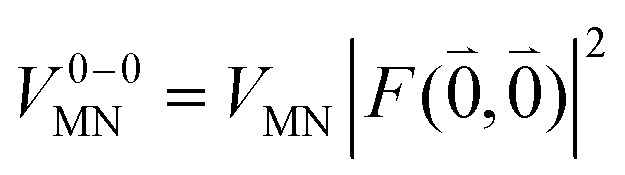
 between the 0–0 transitions that contains the respective Franck–Condon factors for the ground vibrational state 
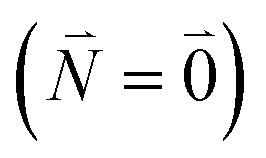
,30
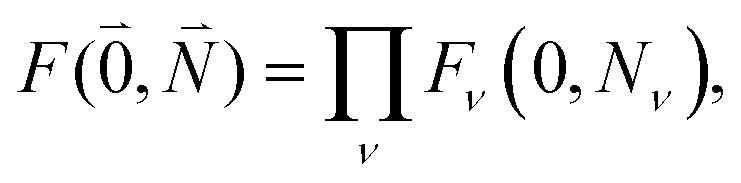
with the overlap integral

31 *F*_*ν*_(0, *N*_*ν*_) = 〈*χ*^(g)^_0_(*Q*_*ν*_)|*χ*^(e)^_*Nν*_(*Q*_*ν*_)〉

between the wave function *χ*^(e)^_*Nν*_(*Q*_*ν*_) of the *N*th vibrational state of the *ν*th mode of the electronic excited state and the vibrational ground state wave function *χ*^(g)^_0_(*Q*_*ν*_) of the *ν*th mode of the electronic ground state of the pigments. Note that we have assumed identical intramolecular Franck–Condon factors of all chromophores, that is, *F*_*ν*_(0,*N*_*ν*_) does not depend on the site index *M*. Here, *Q*_*ν*_ is the normal mode coordinate of the *ν*th mode, that is assumed the same in the two electronic states, neglecting Dushinsky rotation type effects.

Including the intrachromophore exciton–vibrational coupling by the above renormalization of the overall excitonic coupling *V*_*MN*_ with the square of the Franck–Condon factor of the 0–0 transition is valid, as long as the Franck–Condon factors involving excited vibrational states (*e.g. F*_*ν*_(0,*N*_*ν*_)) are sufficiently small, such that the excitonic coupling 
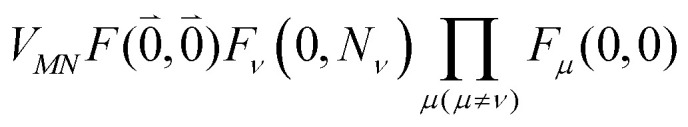
 is small compared to the intramolecular vibrational energy *ħω*_*ν*_. If this inequality does not hold, the mixing between the 0–0 transition of one chromophore with the 0–*N*_*ν*_ transition of the other chromophore would affect the whole spectrum and not just the high-frequency wing.^80^

The quantum chemically determined excitonic coupling *V*_*MN*_ is calibrated below by taking into account the experimental vacuum dipole strength 
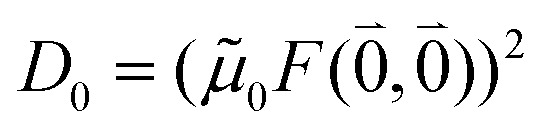
 of the 0–0 transition of Chl *a*, where *μ̃*_0_ = |***μ̃***_0_|. Noting that the quantum chemical vacuum transition dipole moment *μ̃*^*M*^_0_ of pigment *M* is the first moment of the vacuum transition density ***ρ̃***^*M*^(**r**), the calibrated excitonic coupling of the 0–0 transition is given as32
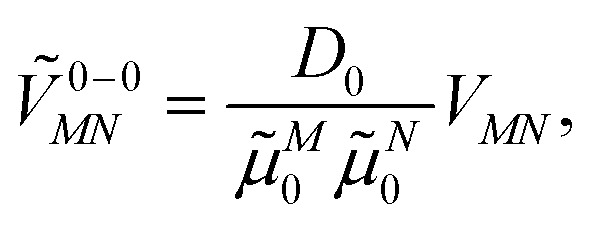
where *V*_*MN*_ is the original quantum chemical excitonic coupling in the dielectric environment and *μ̃*^*M*^_0_ = |***μ̃***^*M*^_0_|. Note that, the experimental Franck–Condon factor of the 0–0 transition is contained in the experimental vacuum dipole strength 
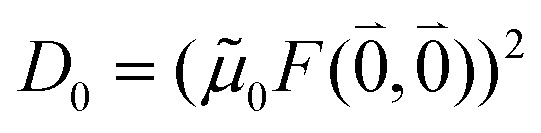
 of this transition. Besides the Franck–Condon factor, the calibration factor contains the ratio 
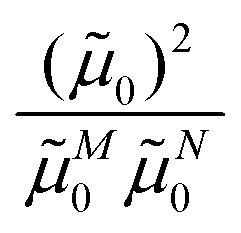
 of experimental and quantum chemical vacuum transition dipole moment magnitudes. This factor corrects for limitations in the quantum chemical calculations. Note that this calibration neglects any change of the transition dipole moments caused by the distortion of the chromophores by their protein environment in WSCP, since *D*_0_ is extrapolated from dipole strengths of isolated Chl *a* measured in different solvents.

As long as the differences between the experimental and calculated vacuum transition dipole moments are small, their effect on the reaction field and thereby on the excitonic coupling can be approximated by the linear scaling factor in eqn (32). Larger changes in the vacuum transition dipole moment most likely affect the reaction field in a non-linear way, and, therefore, cannot be taken into account by such a simple factor. Hence, the calibration of the Poisson-TrEsp and the FMO/PCM couplings should only be valid if the vacuum dipole strength calculated for the chromophores is close to the experimental value. The latter value 
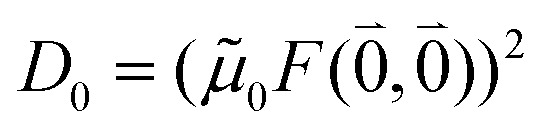
 was determined for the 0–0 transition. In order to compare this value with the quantum chemical vacuum dipole strength we need to know the factor 
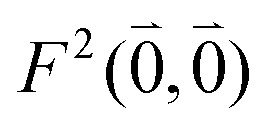
 that can be expressed as^1^ exp(−*S*), where *S* is the overall Huang–Rhys factor33
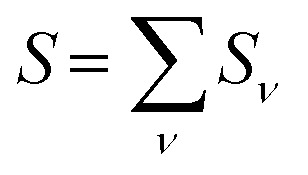
with the individual Huang–Rhys factor *S*_*ν*_ of intramolecular mode *ν*. In the following, an expression is derived for the estimation of *S* based on fluorescence spectra of the molecular dimer and applied to the Chl *a* dimer of WSCP.

### 2.7. Determination of the Huang–Rhys factor of high-frequency intramolecular modes of Chl *a* in WSCP

For the discussion of our results, we extract the Huang–Rhys factor of the high-frequency intramolecular modes of the optical transition of the Chl *a* pigments in WSCP from experimental difference fluorescence line-narrowing spectra (*Δ*-FLN) of WSCP.^49^

Using the standard electronic two-state theory, Pieper *et al.*^49^ arrived at an overall Huang–Rhys factor *S* of 0.8 for the high-frequency modes of the Chl *a* pigments. A subtlety in the analysis is that this Huang–Rhys factor, which determines the relative intensity of the high-frequency vibrational sideband with respect to the 0–0 transition, may depend on the excitonic coupling between the chromophores. Recently, Reppert^81^ approached this problem by numerical diagonalization of a large exciton Hamiltonian that explicitly included 49 high-frequency intramolecular modes per chromophore. These modes were taken from the analysis of *Δ*-FLN experiments,^49^ that can unmask the inhomogeneous broadening. For simplicity, Reppert^81^ assumed an orthogonal orientation of molecular transition dipole moments. His extensive numerical analysis revealed that while there is a reduction of the Huang–Rhys factor of the low-frequency modes by the excitonic coupling, the high-frequency modes essentially exhibit the same Huang–Rhys factor as a local optical excitation of an isolated Chl *a* chromophore, in the parameter range of excitonic couplings that is typical for pigment–protein complexes. We want to investigate how this result changes, if non-orthogonal transition dipole moments (as present in WSCP) are taken into account. It is shown below that the orientation of transition dipoles is indeed critical for the estimation of the Huang–Rhys factor.

The fluorescence at cryogenic temperatures starts from the lowest excited state |*k* = 1〉 of the dimer. As discussed above and also shown by the numerical studies of Reppert,^81^ this state is dominated by the 0–0 transitions of the two chromophores, (eqn (29)), with the exciton coefficients *c*^1^_1_ and *c*^1^_2_ of the lowest exciton state, that are obtained by diagonalization of the exciton Hamiltonian (eqn (22)) for different realizations of static disorder in local excitation energies *E*_*M*_.

From this initial state, a radiative transition is possible to the electronic and vibrational ground state of the complex 
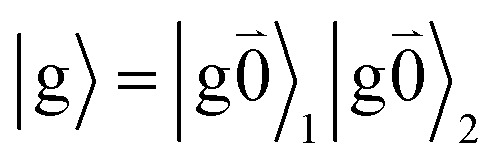
 (the 0–0 transition) with transition dipole moment

34 ***μ***_00_ = 〈*k*|***μ̂***|g〉 = *c*^1^_1_***μ̃***_1_ + *c*^2^_2_***μ̃***_2_.

Here 
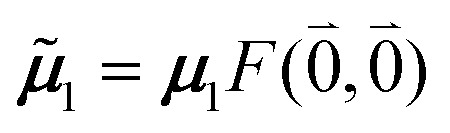
 and 
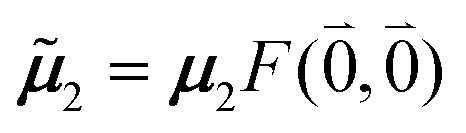
 are the 0–0 transition dipole moments of pigments one and two, respectively, with the total electronic transition dipole moments ***μ***_*i*_ = 〈e|***μ̂***|g〉_*i*_ (*i* = 1 or 2) and the Franck–Condon factors of the 0–0 transition (eqn (30) and (31)), arising from integration of the vibrational degrees of freedom in |*k*〉 and |g〉.

In addition to the 0–0 transition described above, intramolecular vibrations may be excited during the radiative transition from the low energy exciton state |*k*〉 to an electronic ground state 
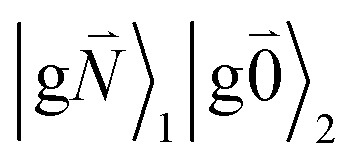
 or 
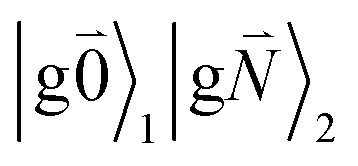
with vibrational excitation in chromophore one or two, respectively. These transitions are visible as discrete peaks in fluorescence line narrowing spectra,^49^ occurring at large vibrational energies (*ħω >* 200 cm^−1^), as compared to the continuous vibrational sideband of the 0–0 transition that has a maximum at low vibrational frequencies (*ħω* ≈ 20 cm^−1^). The vector 
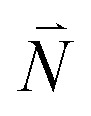
 contains the vibrational quantum numbers *N*_*ν*_ of the different intramolecular modes *ν* excluding the case where all quantum numbers *N*_*ν*_ are simultaneously zero. The respective transition dipole moments are obtained, using eqn (29), as35
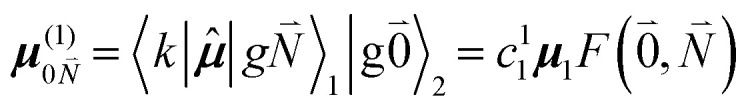
and36
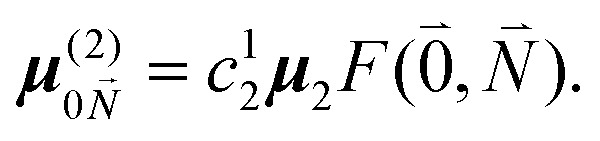
Note that, a simultaneous vibrational excitation of both monomers is impossible. The respective transition dipole moment 
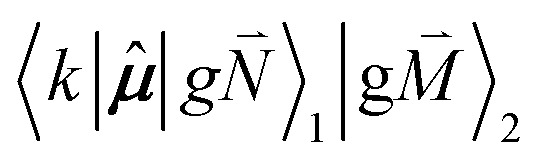
, using eqn (29) for the exciton state |*k* = 1〉, is obtained as 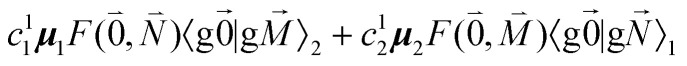 and is found to vanish for 
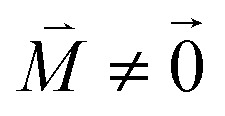
 and, simultaneously, 
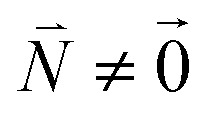
 because of the orthogonality of vibrational wavefunctions of the same electronic state (here the electronic ground state).

The relative intensity of the high-frequency vibrational sideband and 0–0 transition in the fluorescence spectrum can then be estimated using eqn (34)–(36) as37
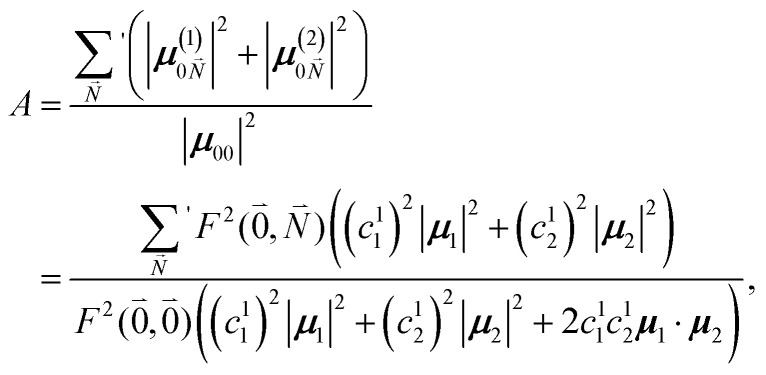
where the prime at the sum excludes the case 
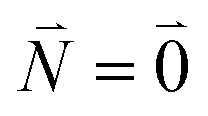
. Taking into account that the pigments have the same magnitude of the transition dipole moment, |***μ***_1_| = |***μ***_2_|, and the normalization of the excitonic wave function, (*c*^1^_1_)^2^ + (*c*^1^_2_)^2^ = 1, results in a relative intensity38
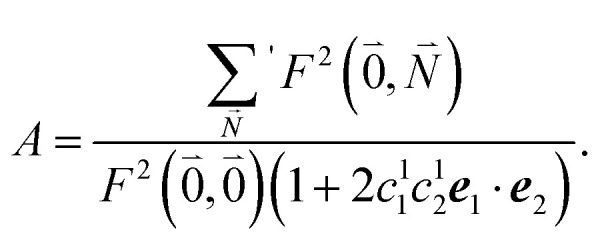
Here, ***e***_1_·***e***_2_ is the scalar product between two unit vectors that are aligned along the transition dipole moments ***μ***_*i*_ = *μ*_*i*_***e***_*i*_ of the two chromophores *i* = 1, 2. We rewrite the sum in the numerator in eqn (38) as39
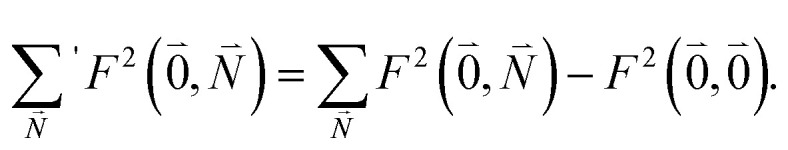


Due to the basis set completion when summed over all possible 
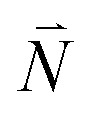
, we have^1^40
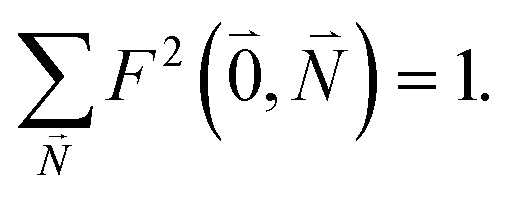
The latter equality reflects the fact that there is only a redistribution of oscillator strength by the electron–vibrational coupling, but the overall oscillator strength of the electronic transition is conserved. By using^1^41
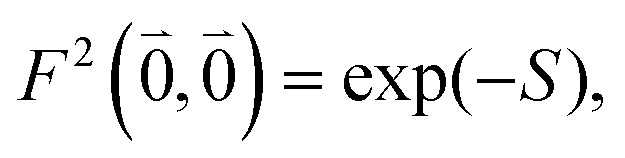
with the overall Huang–Rhys factor *S*, we obtain42
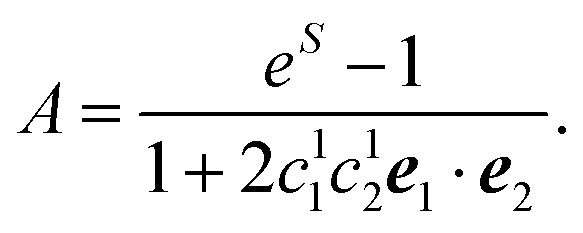


For orthogonal transition dipole moments ***e***_1_·***e***_2_ = 0 and the Reppert rule,^81^

43 *A* = *e*^*S*^ − 1

is recovered, that is also obtained for an electronic two-state system *A* = *e*^*S*′^ − 1. Hence, for orthogonal molecular transition dipole moments we have *S* = *S*′. Reppert formulated this rule in words:^81^ “…, it appears that high-frequency local mode HR factors can be extracted directly from FLN and ΔFLN data with no need for rescaling.” The electronic two-state model results in a Huang–Rhys-factor *S*′ = 0.80 for the coupling of the Chl a chromophores in WSCP to high-frequency intramolecular vibrations,^49^ as noted above. According to eqn (42), derived in this work, the measured intensity ratio *A* (which is *e*^*S*′^ − 1) needs to be interpreted in a new way. The correct Huang–Rhys factor *S*, extracted by taking into account the redistribution of oscillator strength by the interchromophore excitonic couplings, and the Huang–Rhys factor *S*′, extracted with an electronic two-state model, neglecting this redistribution, are related by

44 *S* = ln{(*e*^*S*′^ − 1)(1 + 2〈*c*^(1)^_1_*c*^(1)^_2_〉_dis_***e***_1_·***e***_2_) + 1}.

Here, we have included an average of the product of the exciton coefficients over static disorder in site energies, resulting in 〈*c*^1^_1_*c*^1^_2_〉_dis_ = −0.44 for the present system. Taking into account the angle of 30° between the transition dipole moments of the chromophores, as found in the crystal structure (that is practically identical with the geometry-optimized structure, described above) and the fact that the transition dipole moments are oriented approximately along the NB–ND axis of the pigments (Table 1), we arrive at a Huang–Rhys factor of *S* = 0.23 
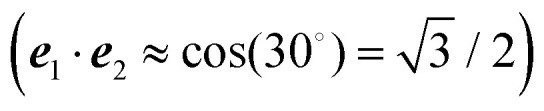
.

**Table 1:** Excitation energies *ω*^*N*^, magnitudes *μ̃*^*N*^ = |***μ̃***^*N*^| and orientations (defined by angles *ϑ*^*N*^ and *φ*^*N*^, see Fig. 4) of the transition dipole moments of individual Chl *a* chromophores *N* computed with FMO0-TDDFT in vacuum (none), or in the protein environment with FMO0/PCM[0] revealing the implicit (impl) and explicit (expl) contributions to the transition dipole moment (eqn (5) and (8) and main text below eqn (13))

*N*	Embedding^a^^b^	*ω* ^*N*^ (eV)	Dipole moments (D)	*ϑ* ^*N*^ (°)	*φ* ^*N*^ (°)
1	None (*μ̃*^1^_0_)	2.140	5.31	0.0	−6.9
	Impl (*μ̃*^1^)	2.102	6.39	−0.1	−6.4
	Impl + expl(*μ̃*^1,screen^)	2.102	3.69	−0.8	−4.8
2	None (*μ̃*^2^_0_)	2.131	5.32	−1.8	−6.6
	Impl (*μ̃*^2^)	2.092	6.41	−1.8	−6.1
	Impl + expl(*μ̃*^2,screen^)	2.092	3.66	−2.4	−4.8
3	None (*μ̃*^3^_0_)	2.136	5.36	0.1	−6.4
	Impl (*μ̃*^3^)	2.098	6.43	0.0	−6.1
	Impl + expl(*μ̃*^3,screen^)	2.098	3.72	−1.1	−4.9
4	None ((*μ̃*^4^_0_)	2.134	5.32	−1.4	−6.2
	Impl (*μ̃*^4^)	2.095	6.40	−1.7	−5.8
	Impl + expl(*μ̃*^4,screen^)	2.095	3.67	−3.6	−4.4

a *ω* ^*N*^ has no explicit embedding contribution.

b The respective symbols for the dipole moments are given in parentheses.

This value is less than one third of the original estimate^49^ that is based on an electronic-two-state theory. This result demonstrates that care should be taken in the estimates of Huang–Rhys factors of high-frequency modes of excitonically coupled pigments, where the local transition dipole moments of the chromophores are non-orthogonal. In this case the Reppert rule,^81^ which would allow for an analysis with the standard two-level system theory, does not apply. The WSCP is an extreme example, since there is a strong redistribution of oscillator strength by the excitonic coupling between the 0–0 transitions. The redistribution is so strong that the low-energy exciton state, from where the fluorescence starts, appears only as a shoulder in the linear absorption spectrum. Since the absolute intensity of the high-frequency vibrational sideband is not influenced by the excitonic coupling,^81^ the relative intensity of this sideband with respect to the 0–0 transition is much larger than for a localized excited state, explaining the large value of *S* estimated before.^49^ Consequently, the present estimate of the Huang–Rhys factor is in the same range as estimates from experimental fluorescence and absorption data of isolated Chl *a* in different solvents^82^ (*S* = 0.28 in ether and in pyridine, *S* = 0.41 in 1-propanol and *S* = 0.38 in 2-propanol).

## 3. Computational details

The solvent screening model for excitonic interactions was implemented for FMO^29,83^ in GAMESS^84,85^ and parallelized with the generalized distributed data interface.^86^ GAMESS was used for all quantum chemical calculations. The initial coordinates of all atoms except hydrogens belonging to Chl *a* chromophores were extracted from the X-ray structure (PDB: 2DRE).^87^ From each chromophore, the phytyl chain was removed, while the C1 carbon was retained. Hydrogen atoms were added using the Jmol software.^88^ In FMO, each chromophore was treated as a separate fragment (4 fragments in total).

Using the CAM-B3LYP functional^89^ with the 6-31-G* basis set, a geometry optimization was performed for each isolated chromophore in vacuum separately in a two-step process. In the first step, an optimization was done with nitrogen coordinates held fixed. A second optimization was performed without any constraints. This two-step procedure was chosen to preserve the relative orientation of the chromophores as much as possible. The inter-pigment distances in WSCP are large enough, so that no steric clashes were observed after merging the coordinates of the geometry-optimized pigments. The obtained coordinates are listed in Table S2 (ESI†). These coordinates were used in all subsequent calculations on the WSCP complex unless otherwise noted. As a check, the complete optimization was also applied to Chl *a* dimers revealing very small differences in atomic coordinates (Table S3, ESI†) and electronic structure (Table S4 and S5, ESI†), as compared to the monomer optimization described above.

FMO/PCM calculations on the WSCP complex were done using TDDFT with the range-separated CAM-B3LYP exchange correlation (XC) functional and the 6-31+G* basis set for the transition from the ground singlet state S_0_ to the first excited singlet state S_1_ of each chromophore, unless otherwise noted. In TDDFT/PCM, the ground state is computed for DFT/PCM using the static dielectric constant *ε*_s_. Then, the TDDFT equations are solved in the presence of the solvent field. For the latter step, two scenarios are possible:^90^ (a) the non-equilibrium case suitable for vertical excitations, where *ε* is set to be the optical dielectric constant (*ε* = *n*^2^) and (b) the equilibrium case suitable for studying energy minima for excited states, in which case the static dielectric constant *ε*_s_ is used in TDDFT. In FRET, the former approach is taken, because during the excitation energy transfer there is no time for nuclear relaxation.

For the calculations involving a continuum solvent, IEF-PCM was used in the non-equilibrium formulation of TDDFT (IEF-PCM is the appropriate model for small dielectric constants^91,92^). The static dielectric constant *ε*_s_ was set to 4 (a typical value for proteins^93^), while the optical dielectric constant *ε* = 2, as determined earlier from an analysis of the oscillator strength of protein-extracted chlorophylls.^45^ The molecular cavity was constructed in PCM using the Bondi radii,^94^ multiplied by a scaling factor of 1.2, such that the cavity surface is at the solvent accessible surface rather than the van der Waals surface.

The transition density between two states has an arbitrary phase. As a consequence, its first moment, that is, the transition dipole moment also has an arbitrary phase. Physically observable properties do not depend on the phase, but if one is to compare transition dipoles in various calculations, it is necessary to devise a scheme for fixing the phase (as the phase is real, the issue is whether to multiply by −1 or not). For simplicity, all couplings in which the pigment with a reversed transition dipole moment is involved are also multiplied by a factor of −1. The convention for the transition dipole direction used in the present work is defined in the following. Note, however, that this multiplication of couplings and transition dipole moments has no influence on the observables, *e.g.*, the linear absorption spectrum.

**Fig. 4:**
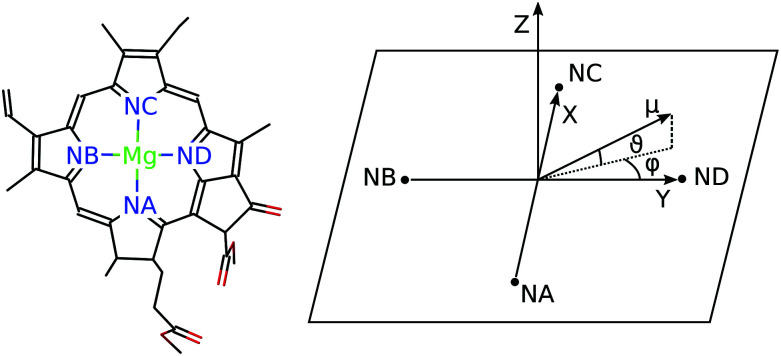
The left part shows a single Chl *a* chromophore without its phytyl tail illustrating the naming scheme for the nitrogen atoms. The right part shows the orientation of the axes *Y*, *X* and *Z* with respect to the nitrogen atoms NA, NB, NC, and ND. The angles *ϑ* and *φ* define the direction of the transition dipole moment ***μ*** with respect to these axes.

For discussing the dipole moments and excitonic couplings, it is necessary to define the orientation of the former, as noted above. In each individual Chl *a* chromophore the four nitrogen atoms NA, NB, NC, ND can be used to define a local coordinate system. *X* is the direction NA–NC, while the direction NB–ND is *Y*. The direction perpendicular to the *XY* plane is *Z*. Together, *Y*, *X* and *Z* form a right handed coordinate system (Fig. 4). The phase of the transition dipole moment is chosen such that its projection on the *Y*-axis is positive. The angle between a particular transition dipole moment and the respective molecular *XY* plane is denoted *ϑ*. A positive angle *ϑ* means that the component of the transition dipole moment in the direction of *Z* is positive. *φ* is the angle between a particular transition dipole moment and the molecular *Y* direction measured in the *XY* plane. A positive angle *φ* means that the component of the transition dipole moment in *X* direction is positive.

When the dipole model in eqn (11)–(13) is applied, one has to define a distance between two chromophores *R*_*MN*_. We found that the PDA performs best if the point dipoles are placed at the geometric centers of the four nitrogen atoms (see Fig. 4 and Table S1, ESI†),45
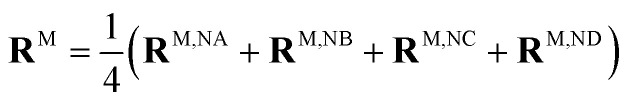


Using these centers, the distance is calculated as *R*_*MN*_ = |**R**^*M*^ − **R**^*N*^|. For a comparison with the Poisson-TrEsp method, atomic transition charges were obtained from a fit of the ESP of the transition densities of the chromophores^71,95^ using the CHELPG grid^96^ implemented in the potential derived charges (PDC) method.^95^ In the fit, the dipole moment of the fitted charges was constrained to the value obtained from the transition density (eqn (5)). The Poisson-TrEsp calculations were performed with the program MEAD^97,98^ (see ESI† for more information).

Besides the excitonic couplings, the following parameters are used in the calculation of the optical spectra, as determined before.^59^ A mean transition energy *Ē*_1_ = *Ē*_2_ of the two Chl *a* chromophores corresponding to a wavelength of 675 nm, a full width at half maximum *Δ*_inh_ of 170 cm^−1^ for the Gaussian distribution function of the local transition energies of the chromophores, a Huang–Rhys factor *S* = 0.8 for the low-frequency part of the spectral density, that is assumed to have a functional form, extracted earlier from fluorescence line-narrowing spectra of the B777 pigment–protein complex.^76^ In addition, a pure dephasing time of 2750 fs was assumed as determined earlier^59^ from a simulation of hole-burning spectra of the WSCP complex and comparison with experimental data.^50^ The pure dephasing time describes the finite width of the 0–0 line, but its effect is masked by the inhomogeneous broadening in the present ensemble spectra, which practically do not depend on the value used for this dephasing time.

## 4 Results

### 4.1 Transition dipoles and excitonic couplings calculated with FMO0 (no mutual polarization of the chromophores)

The computed excitation energies and dipole moments of the 4 Chl *a* chromophores in WSCP are shown in Table 1. For the isolated chromophores (in vacuum) the angle *ϑ* varies between 0.1° and −1.8° and *φ* varies between −6.2° and −6.9°, which means that the S_0_–S_1_ transition dipole lies in the *XY* plane and is practically oriented in the *Y*-direction (NB–ND axis) with a slight clockwise rotation (Fig. 4) in agreement with similar TDDFT calculations on Chl *a.*^74^ Including the implicit polarization by the protein/solvent environment (“impl” in Table 1) enhances the transition dipole moment of the pigment by about 20% while the change in direction is very small (<0.4° for *ϑ* and <0.6° for *φ*). Adding the screening contribution due to the protein/solvent environment (“impl + expl” in Table 1) reduces the magnitude of the effective dipole moment by about 40% and causes a slight rotation (<2.0° for *ϑ* and <1.7° for *φ*).

The couplings computed from the transition density are presented in Table 2. Three coupling values for each pair of chromophores are given: the vacuum coupling *V*^ES^_*MN*_(*ε* = 1), the protein-embedded coupling *V*^impl^_*MN*_(*ε* = 2), obtained by taking into account the mutual dynamic polarization of the chromophores and the protein/solvent environment (reaction field effects), and the total coupling *V*^impl^_*MN*_(*ε* = 2) + *V*^expl^_*MN*_(*ε* = 2) including, in addition, the explicit environmental contributions, representing screening effects.

**Table 2:** FMO0/PCM[0] excitonic couplings (cm^−1^) between WSCP chromophores *M* and *N*, in vacuum (*V*^ES^_*MN*_(*ε* = 1)), and in the protein/solvent embedding with implicit (reaction field) *V*^impl^_*MN*_(*ε* = 2) and explicit (screening) *V*^expl^_*MN*_(*ε* = 2) contributions^a^

*M*	*N*	*V* ^ES^_*MN*_(*ε* = 1)	*V* ^impl^_*MN*_(*ε* = 2)^a^	*V* ^impl^_*MN*_(*ε* = 2) + *V*^expl^_*MN*_(*ε* = 2)^a^	*f* ^rf^_*MN*_	*s*_*MN*_
3	4	147	209	130	1.423	0.621
1	2	142	204	126	1.429	0.621
2	3	35	48	29	1.383	0.600
1	4	32	44	27	1.396	0.603
2	4	10	15	10	1.504	0.682
1	3	9	13	9	1.494	0.713

a Using TDDFT calculations with the CAM-B3LYP XC-functional. The last two columns contain the reaction field and screening factors, defined in eqn (9) and (10), respectively.

In Table 2 one can see three groups of couplings with two couplings in each group: (I) 3–4 and 1–2 (II) 2–3 and 1–4, (III) 2–4 and 1–3, with large, intermediate and small excitonic couplings, respectively. The reason for this grouping can be inferred from Fig. 1: the WSCP complex has an approximate *D*_2_ symmetry. This means that every relative orientation between pigment pairs appears twice. The vacuum couplings are enhanced due to the implicit dynamic polarization effect of the protein/solvent environment by the reaction field factor *f*^rf^_*MN*_ that varies between 1.38 and 1.50. The coupling is reduced by the explicit dynamic polarization of the environment by a screening factor *s*_*MN*_ varying between 0.60 and 0.71.

These factors are somewhat larger for group (III) than for group (I) and group (II). This variation is rationalized further below in terms of a rotation of the transition dipole moments. Interestingly, there is a certain compensation between the implicit and the explicit protein-embedding effects, such that the overall scaling factor between the vacuum and protein couplings *f*^rf^_*MN*_*s*_*MN*_ is not so far from unity.

### 4.2 Couplings calculated with FMO1: the role of the mutual polarization of chromophores

In order to investigate the role of the mutual polarization of the electronic ground state of the chromophores, the results of Tables 1 and 2 (obtained with FMO0: no pigment–pigment polarization) can be compared with those of Tables 3 and 4, respectively, obtained with FMO1 (with such polarization). The polarization in FMO1 is taken into account by including in the Hamiltonian a self-consistently determined embedding potential describing the electrostatic field of the ground state of fragments.^29^

**Table 3:** Same as in Table 1, but obtained with FMO1/PCM[1]

*N*	Embedding	*ω* ^*N*^ (eV)	*μ* ^*N*^(D)	*ϑ* ^*N*^ (°)	*φ* ^*N*^ (°)
1	None	2.150	5.28	0.0	−6.2
	Impl	2.115	6.32	−0.1	−5.7
	Impl + expl	2.115	3.65	−0.7	−4.3
2	None	2.140	5.27	−1.8	−6.4
	Impl	2.101	6.33	−1.8	−5.7
	Impl + expl	2.101	3.61	−2.4	−4.4
3	None	2.147	5.31	0.1	−5.7
	Impl	2.112	6.35	0.1	−5.6
	Impl + expl	2.112	3.67	−1.1	−4.4
4	None	2.144	5.28	−1.3	−5.6
	Impl	2.106	6.32	−1.6	−5.3
	Impl + expl	2.106	3.62	−3.6	−3.9

**Table 4:** Same as in Table 2, but obtained with FMO1/PCM[1]

*M*	*N*	*V* ^ES^_*MN*_(*ε* = 1)	*V* ^impl^_*MN*_(*ε* = 2)	*V* ^impl^_*MN*_(*ε* = 2) + *V*^expl^_*MN*_(*ε* = 2)	*f* ^rf^_*MN*_	*s*_*MN*_
3	4	144	203	126	1.413	0.621
1	2	140	199	123	1.421	0.621
2	3	34	47	28	1.369	0.600
1	4	31	43	26	1.381	0.602
2	4	10	15	10	1.494	0.680
1	3	9	13	9	1.466	0.710

Due to the polarization, the excitation energies are increased by ∼10 meV, while the transition dipole moments generally are 1–2% smaller. It can be noted that the embedding shifts both the ground and excited state energies (although not equally) and thus the effect on the transition energy is relatively weak. As can be seen from Fig. 4, chlorophylls are neutral non-polar molecules, although they do include a cation Mg^2+^, but its charge is compensated by the donating lone pairs on the nitrogens. In addition, the centers of chlorophylls are quite far separated from each other (Table S1, ESI†). Thus, the polarization of chlorophylls by each other is not very strong. The chromophore polarization lowers the couplings by a few percent at most (Table 4*vs.*Table 2), which can be rationalized by the slightly smaller transition dipoles (Table 3*vs.*Table 1). The polarization has a negligible effect on the screening factor *s*_*MN*_ and a very small effect on the reaction field factor *f*^rf^_*MN*_.

The small effect of the mutual polarization between the chromophores on the excitonic couplings obtained with FMO1, using an atomistic description of the chromophores, is consistent with the weak dependence of the excitonic coupling on the static dielectric constant *ε*_s_ used in FMO0 and FMO1 to describe the polarization of the electronic ground state of the chromophores by the homogeneous dielectric representing the protein and solvent environment. The excitonic couplings vary by at most 0.2% when *ε*_s_ is varied between *ε*_s_ = 2 and *ε*_s_ = 20 (Table S19, ESI†). Note that the polarization of the excited states of the pigments can be expected to be similarly small, since the change in permanent dipole moment between excited and ground state of Chl *a* is small.^99^

### 4.3 Excitonic couplings in the point-dipole approximation

**Fig. 5:**
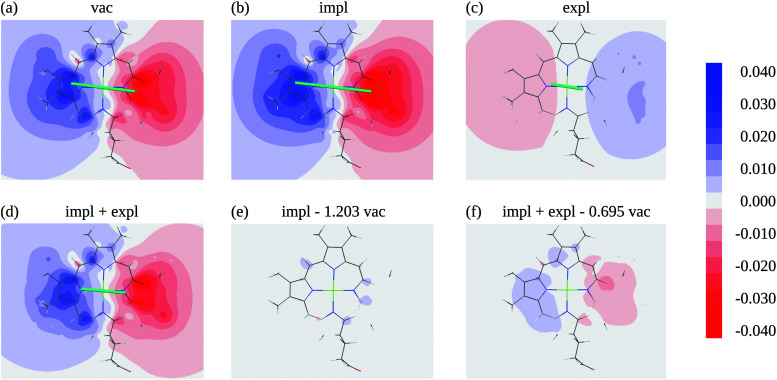
Electrostatic potential (ESP) of chromophore 1 evaluated in the molecular plane in vacuum (a) and in protein/solvent environment (b–d). In the ESP in panel (b) the implicit solvent contribution is included (*φ̃*^1^(**r**), eqn (3)), the ESP in panel (c) contains the explicit solvent contribution (*φ*^1,expl^(**r**), eqn (7)), panel (d) shows the overall ESP in the protein/solvent environment, panel (e) contains the difference between the implicit ESP in panel (b) and a scaled vacuum ESP of panel (a); the scaling factor *μ̃*^1^/*μ̃*^1^_0_ is the ratio of the magnitudes of the pigment transition dipole moment *μ̃*^1^ = 6.39 D and the moment in vacuum *μ̃*^1^_0_ = 5.31 D, panel (f) shows the difference between the overall ESP in (c) and a scaled vacuum ESP of (a); the scaling factor *μ̃*^1,screen^/*μ̃*^1^_0_ is the ratio of the magnitudes of the screened transition dipole moment *μ̃*^1,screen^ = *|****μ̃***^1^ + ***μ̃***^1,expl^*|* = 3.69 D, where ***μ̃***^1,expl^ (eqn (8)) is the dipole moment of the solvent polarization induced by the transition density of chromophore 1, and the moment in vacuum. The teal lines in (a)–(d) show the direction and magnitude of the transition dipole moment. All quantum chemical calculations for this figure were performed with TDDFT using the CAM-B3LYP functional.

In order to judge the plausibility of the PDA and to investigate reaction field and screening effects, the ESP *φ̃*^1^(**r**) (eqn (3)) of the molecular transition density of pigment 1 including also the ESP of the dynamic solvent polarization induced by the latter *φ*^1,expl^(**r**) (eqn (7)) is plotted in Fig. 5 in the molecular plane defined by the nitrogen atoms NA, NB, NC, and ND. The potential is clearly dominated by the dipole contribution. A visual comparison of panels (a), (b) and (d) in Fig. 5 shows that the change of the potential from vacuum to protein-embedding (both with and without the explicit contribution from the solvent polarization *φ*^1,expl^(**r**) can mainly be attributed to a global amplification or attenuation. The attenuation is due to the sign inversion of *φ*^1,expl^(**r**) with respect to the ESP in vacuum in Fig. 5(c) and (a), respectively. This observation is confirmed in Fig. 5(e) and (f) where the differences of the ESPs in the protein/solvent environment with respect to the ESP in vacuum scaled with the ratio of the respective transition dipole moment magnitudes *μ̃*^1^/*μ̃*^1^_0_ and (*μ̃*^1^ + *μ̃*^1,expl^)/*μ̃*^1^_0_, respectively, are shown, where *μ̃*^1^_0_ is the magnitude of the vacuum transition dipole moment of chromophore 1. The potential differences are somewhat larger if both implicit and explicit environmental contributions are taken into account (Fig. 5f) as compared to the case where only implicit effects contribute (Fig. 5e).

The excitonic couplings in PDA computed with FMO0 are shown in Table 5. From a comparison to the results obtained with the transition density (Table 2), it can be seen that the dipole approximation is quite accurate, especially in the case where both implicit and explicit environmental effects are considered (Table 5, column 5). The PDA couplings are a few percent larger than the transition density couplings. Interestingly, the increase of the couplings in PDA with respect to the transition density couplings is somewhat stronger in vacuum than in the medium, indicating a partial error compensation effect. Apparently, in PDA the larger vacuum couplings are partially compensated by the stronger screening (smaller *s* values). While the order of the reaction field and screening factors *f*^rf^_*MN*_ and *s*_*MN*_ in Tables 5 and 2 is not the same, the factors in group (III) (couplings 2–4 and 1–3) are the largest in both tables.

**Table 5:** Same as in Table 2, but in point-dipole approximation (eqn (11)–(13))

*M*	*N*	*V* ^ES,*μ*^_*MN*_ (*ε* = 1)	*V* ^impl,***μ***^_*MN*_ (*ε* = 2)	*V* ^impl,***μ***^_*MN*_ (*ε* = 2) + *V*^expl,***μ***^_*MN*_ (*ε* = 2)	*f* ^rf,*μ*^_*MN*_	*s* ^*μ*^_*MN*_
3	4	167	241	136	1.441	0.566
1	2	161	233	133	1.449	0.569
2	3	36	51	30	1.444	0.581
1	4	34	50	29	1.447	0.578
2	4	8	12	8	1.527	0.635
1	3	7	11	7	1.503	0.626

From analyzing the trends of the three kinds of couplings (vacuum, impl, impl + expl), it can be hypothesized that the results obtained with the transition density can be accurately modeled by a change in the transition dipole vector. This change can be separated into two aspects: a change in the vector length and a change in the direction (a rotation). To separate these two aspects, the vacuum PDA couplings in Table 5 were scaled by factors containing different transition dipole magnitudes46
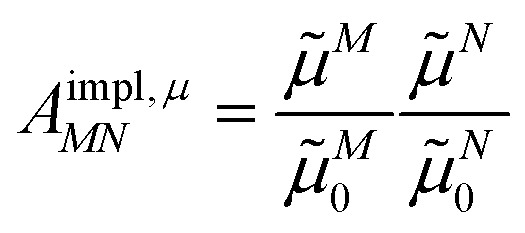
and47
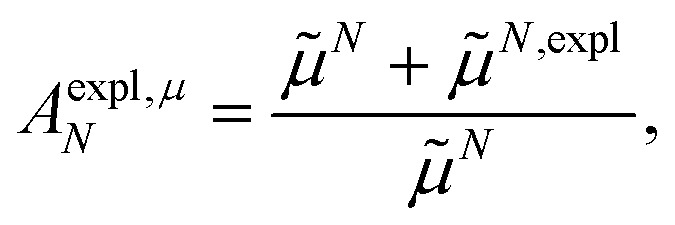
where *μ̃*^*M*^_0_ and *μ̃*^*N*^_0_ are the magnitudes of the vacuum transition dipole moments of chromophores *M* and *N*, respectively. The dipole moment magnitudes *μ̃*^*M*^ and *μ̃*^*N*^ are computed for pigments in the protein/solvent environment, and *μ̃*^*N*,expl^ is the magnitude of the explicit transition dipole moment of the environment induced by the transition density of chromophore *N* (eqn (8)).

The scaled couplings shown in Table 6 agree very well with the respective values in Table 5. Hence, the magnitude of the transition dipole moments is the decisive factor. Also, the polarization and screening factors factors *f*^rf,*μ*^_*MN*_ and *s*^*μ*^_*MN*_ in Tables 5 and 6 are very similar. A closer inspection shows that for group (III) the screening factors (*s*^*μ*^_24_, *s*^*μ*^_13_) are more similar to the values for group (I) and (II) in Table 6 than in Table 5. Hence, this difference can be attributed to an effective rotation of transition dipole moments of the pigments by the polarization of the environment.

**Table 6:** Excitonic couplings *V*^*μ*^_*MN*_ (*ε* = 1) from the 3rd column of Table 5 are scaled by the ratios *A*^impl,*μ*^_*MN*_ of the magnitudes of the embedded and vacuum dipole moments (eqn (46)) and by the ratio of the magnitude of the screened and the unscreened dipole moment *A*^expl,*μ*^_*MN*_ (eqn (47)) as an approximation for the implicit and the overall excitonic couplings in the 4th and 5th column of Table 5, respectively. The last two columns contain the resulting approximations for the reaction field and screening factors

*M*	*N*	*A* ^impl,*μ*^_*MN*_ *V* ^*μ*^_*MN*_ (*ε* = 1)	*A* ^expl,*μ*^_*MN*_ *A* ^impl,*μ*^_*MN*_ *V* ^*μ*^_*MN*_ (*ε* = 1)	*f* ^rf,*μ*^_*MN*_	*s* ^*μ*^_*MN*_
3	4	241	138	1.444	0.573
1	2	233	133	1.448	0.570
2	3	52	30	1.446	0.578
1	4	50	28	1.446	0.573
2	4	12	7	1.449	0.573
1	3	10	6	1.443	0.578

### 4.4 Comparison of the FMO0/PCM[0] and the Poisson-TrEsp couplings and their calibration

For comparison, Poisson-TrEsp calculations were performed with the atomic charges obtained from the transition density in protein (FMO0/PCM[0]) and in vacuum (FMO0). The results are shown in Table 7 and can be compared with the original excitonic couplings obtained with the FMO0/PCM[0] method (Table 2). An excellent agreement is obtained between the vacuum Poisson-TrEsp couplings *V*^P-TrEsp^_*MN*_(*ε* = 1) of the unpolarized chromophores in vacuum (*q̃*^*M*^_*α*_(*ε* = 1)) as well as well as the chromophores polarized by the protein/solvent environment (*q̃*^*M*^_*α*_(*ε* = 2)) shown in Table 7 and the corresponding results in Table 2. Including the dielectric continuum in the Poisson-TrEsp calculations leads to a reduction (screening) of the excitonic coupling, as described by the respective screening factors *s*^P-TrEsp^_*MN*_ in Table 7. Identical screening factors are obtained for the two sets of charges, indicating that, except for their magnitude, the polarized and unpolarized transition densities of the chromophores are very similar, as noted already above. Moreover, the screened excitonic couplings *V*^P-TrEsp^_*MN*_(*ε* = 2) of the polarized transition densities obtained with Poisson-TrEsp agree quite well with the total excitonic couplings obtained in FMO0/PCM[0] in Table 2. Hence, Poisson-TrEsp provides an accurate description of the screening part of the FMO[0]/PCM[0] calculations. As discussed below in more detail, this result reflects similarities in the quantum mechanical perturbation theory and the interpretation of the quantum-mechanical results in terms of classical electrostatics.^43,67,100^

**Table 7:** Excitonic couplings (cm^−1^) between WSCP chromophores *M* and *N* calculated with the Poisson-TrEsp method in vacuum (*ε* = 1) or in a dielectric medium (*ε* = 2) using the sets of atomic transition charges *q̃*^*M*^_*α*_ obtained from FMO0 in vacuum (*ε* = 1) or FMO0/PCM[0] in a protein/solvent embedding (*ε* = 2); *s*^P-TrEsp^_*MN*_ is the screening factor (eqn (17))

*M*	*N*	*q̃* ^*M*^_*α*_ (*ε* = 1) from FMO0			*q̃* ^*M*^_*α*_ (*ε* = 2) from FMO0/PCM[0]		
		*V* ^P-TrEsp^_*MN*_ (*ε* = 1)	*V* ^P-TrEsp^_*MN*_ (*ε* = 2)	*s* ^P-TrEsp^_*MN*_	*V* ^P-TrEsp^_*MN*_ (*ε* = 1)	*V* ^P-TrEsp^_*MN*_ (*ε* = 2)	*s* ^P-TrEsp^_*MN*_
3	4	147	90	0.62	211	130	0.62
1	2	141	87	0.62	202	125	0.62
2	3	35	21	0.60	48	29	0.60
1	4	32	19	0.60	44	27	0.60
2	4	10	7	0.74	14	11	0.74
1	3	8	6	0.78	13	10	0.77

Because the polarization of the transition density in the PCM calculations effectively corresponds to a multiplication of the vacuum transition dipole moment by a constant polarization factor 
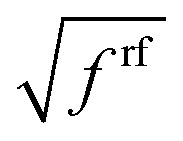
, we can take into account this polarization by just multiplying the vacuum transition charges in Poisson-TrEsp by this factor. To correct for limitations in the quantum chemical calculations on the isolated chromophores, the experimental vacuum transition dipole moment of the 0–0 transition of Chl *a* is taken into account, obtained from the empirical relation of Knox,^60^ discussed above. The transition charges *q̃*^*M*^_*α*_ used in the Poisson-TrEsp calculations should be scaled such that the first moment, *i.e.*, the transition dipole moment, satisfies the relation48
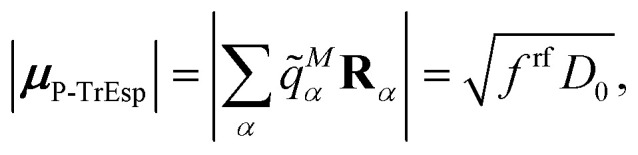
where *f*^rf^ is the average reaction field factor, obtained by the present FMO0-PCM[0] calculations, and *D*_0_ is the experimental vacuum dipole strength of the 0–0 transition.

For the present Chl *a* chromophores,^60^*D*_0_ = 20.2 D^2^ and from the reaction field factors *f*^rf^_*MN*_ in Table 2, an average polarization (reaction field) factor *f*^rf^ = 1.44 is obtained. These two factors give rise to a transition dipole moment of *μ*_P-TrEsp_ = 5.39 D for Chl *a*. Hence, considering the present Poisson-TrEsp (P-TrEsp) calculations, the calibration factor is49
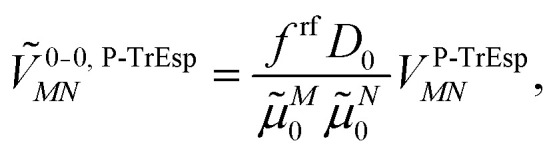
where *V*^P-TrEsp^_*MN*_ is the coupling obtained for the uncalibrated quantum chemical transition charges of the isolated chromophores *M* and *N* that result in vacuum transition dipole moment magnitudes *μ̃*^*M*^_0_ and *μ̃*^*N*^_0_, respectively.

Note, that the experimental vacuum dipole strength *D*_0_ of the 0–0 transition also serves as a calibration factor of the FMO/PCM couplings. In this case the polarization effect is explicitly included in the calculations and, therefore, the calibrated FMO/PCM coupling is given by eqn (32). The rationale behind this scaling is that without environment the transition densities should be consistent with the experimental vacuum transition dipole moments.

The calibrated couplings of the FMO0/PCM[0] approach and the Poisson-TrEsp values are compared in Table 8, revealing excellent agreement. In the calculations presented in Table 7, “direct” transition charges resulting from the PCM calculations are used in the Poisson-TrEsp calculations. In contrast, in the Poisson-TrEsp calculations in the 4th column of Table 8 only the average reaction field factor is introduced. Nevertheless, these Poisson-TrEsp calculations still give identical results to the FMO0/PCM[0] method.

**Table 8:** Calibrated excitonic couplings *Ṽ*^0–0^_*MN*_ (cm^−1^) between 0–0 transitions of WSCP chromophores *M* and *N*

*M*	*N*	FMO0/PCM[0]^a^	Poisson-TrEsp (*q̃*^*M*^_*α*_ (*ε* = 1) from FMO0)^b^^c^	Poisson-TrEsp (original)^d^	Poisson-TrEsp (improved)^b^^e^	QM/MMPol^a^^f^
3	4	92	92	62	86	76 (188)
1	2	90	90	60	83	75 (183)
2	3	21	21	17	24	12 (31)
1	4	19	20	17	24	12 (31)
2	4	7	7	6	8	4 (9)
1	3	6	6	5	7	6 (15)

a Calibrated as described in the text and in eqn (32).

b Calibrated as described in the text and in eqn (49).

c The vacuum transition charges *q̃*^*M*^_*α*_(*ε* = 1) of the FMO0 method (with TDDFT/CAM-B3LYP) are used.

d Calibrated as described in the text and in eqn (49), but setting *f*^rf^ = 1 and using the vacuum dipole strength _0_ = 21.0 D^2^ of the 0–0 transition of Chl *a*,^60^ obtained from an analysis of the dipole strength using an empty spherical cavity local field factor (the blue line in Fig. 3).

e Transition dipole moments *μ̃*^*M*^_0_ and *μ̃*^*N*^_0_ and the uncalibrated Poisson-TrEsp couplings *V*^P-TrEsp^_*MN*_ are obtained as in the original Poisson-TrEsp method.^41–43^

f Uncalibrated values in parentheses are taken from Table 1 of ref. 52.

Next, we investigate how the standard Poisson-TrEsp calculations that are based on atomic transition charges of the non-hydrogen atoms, obtained from a fit of the ESP of the transition density of isolated geometry optimized Chl *a*,^71^ can be improved by taking into account the average reaction field factor *f*^rf^ = 1.44 obtained from the present FMO0/PCM[0]/TDDFT/CAM-B3LYP calculations. The standard Poisson-TrEsp couplings are obtained by placing the TDDFT/B3LYP transition charges (taken from Table 1 in the supporting information of ref. 71) on the respective non-hydrogen atom positions of the chromophores in the crystal structure (PDB: 2DRE), resulting in uncalibrated Poisson-TrEsp couplings *V*^P-TrEsp^_*MN*_ and transition dipole moment magnitudes *μ̃*^*M*^_0_ and *μ̃*^*N*^_0_. The calibrated couplings of the 0–0 transition *Ṽ*^0–0,P-TrEsp^_*MN*_ are obtained from *V*^P-TrEsp^_*MN*_ using eqn (49), but setting the reaction field factor *f*^rf^ = 1 and applying *D*_0_ = 21.0 D^2^ of the empty spherical cavity model. The couplings obtained with this original Poisson-TrEsp approach (Table 8, 5th column) are significantly smaller than the FMO0/PCM[0] values. The largest couplings differ by about 30%. In the improved Poisson-TrEsp approach we use the same uncalibrated couplings *V*^P-TrEsp^_*MN*_ and transition dipole moment magnitudes *μ̃*^*M*^_0_ and *μ̃*^*N*^_0_ as in the original approach, but include the average reaction field factor *f*^rf^ = 1.44 from the present FMO0/PCM[0]/TDDFT/CAM-B3LYP calculations. In addition, we replace the *D*_0_ value of the empty spherical cavity model by the *D*_0_ = 20.2 D^2^ obtained from the empirical fit of the experimental dipole strength of the 0–0 transition of Chl *a* (the black line in Fig. 3). The resulting excitonic couplings *Ṽ*^0–0,P-TrEsp^_*MN*_ are within 10% of the FMO0/PCM[0] values and the values for the strongly coupled pigments are close to the value of 83 cm^−1^ that has been inferred^59^ from a fit of the optical spectra. The latter are analyzed next.

### 4.5 Optical spectra of WSCP

**Fig. 6:**
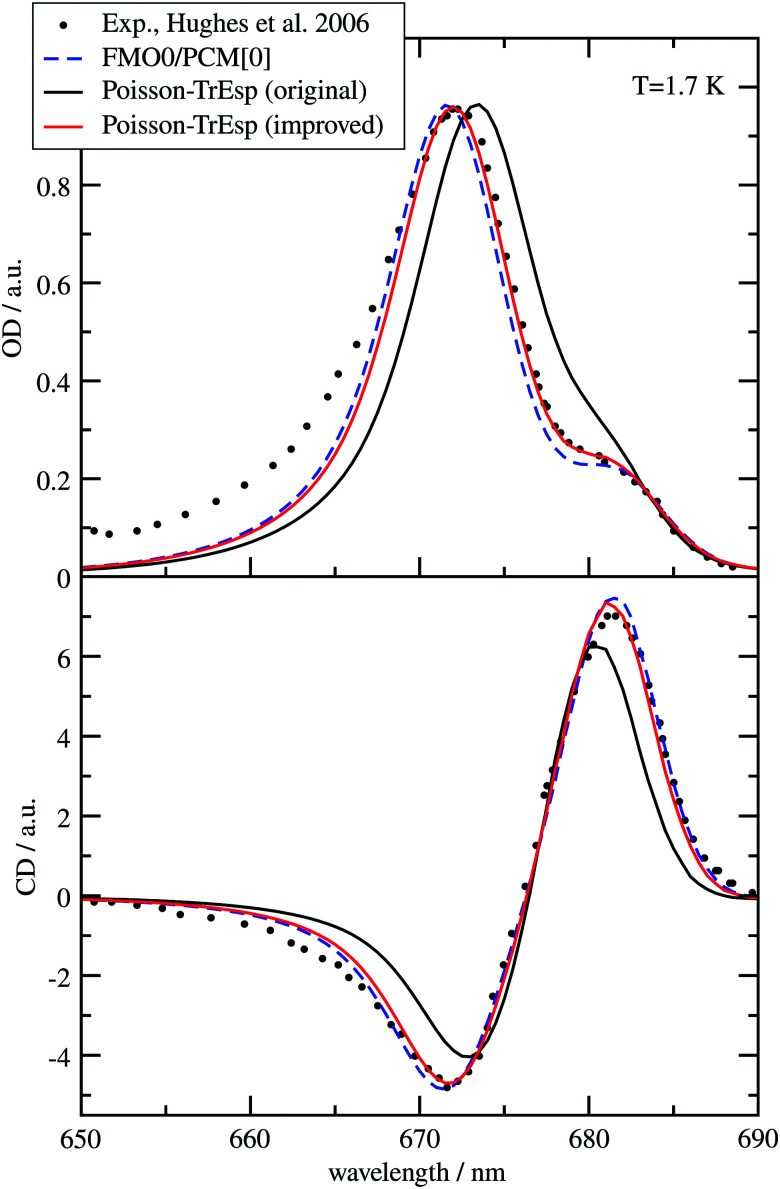
Comparison of low-temperature optical spectra of WSCP dimers calculated with calibrated excitonic couplings (the average of 1–2 and 3–4 couplings in the 3rd, 5th and 6th column of Table 8), obtained in the present work (lines), with experimental absorption (upper part) and circular dichroism (lower part) spectra^48^ (circles). The black solid line is obtained for the standard Poisson-TrEsp coupling that neglects reaction field effects, the blue dashed line is calculated with the FMO0/PCM[0] coupling and the solid red line with the improved Poisson-TrEsp coupling.

The optical spectra obtained for the calibrated FMO0/PCM[0] excitonic couplings and couplings obtained with the original and the improved Poisson-TrEsp approach are compared with the experimental data^48^ in Fig. 6. The parameters of our exciton model (namely, local excitation energy, width of the inhomogeneous distribution function for the local excitation energies, Huang–Rhys-factor of the low-frequency part of the spectral density, pure-dephasing time) have been inferred before from comparison of calculated and measured absorption and hole-burning spectra.^59^

Due to the open-sandwich geometry of the transition dipole moments of the two chromophores in the dimer, the upper exciton state has the major part of the oscillator strength and the low-energy exciton transition appears only as a shoulder in the linear absorption spectrum. The obtained excitonic CD spectrum clearly shows the two transitions, that have a sign-inverted rotational strength of the same magnitude. Whereas the original Poisson-TrEsp method underestimates the splitting between the two exciton transitions, the FMO[0]/PCM[0] and improved Poisson-TrEsp couplings result in optical spectra that fit the experimental optical spectra very well. Because the high-frequency intramolecular modes were not explicitly included in the calculations of the spectra, deviations appear between the calculated and measured absorption spectra (Fig. 6) at high energies (*λ* < 665 nm). Whereas the low-frequency vibrations, which appear close to the 0–0 transitions determining the main part of the spectra (*λ >* 665 nm), are included in our lineshape theory, the high-frequency intramolecular vibrations would have to be treated separately, because an unphysical scaling of the Huang–Rhys factors with the inverse participation ratio of exciton states would result.^81^ Note however that, the effect of the intramolecular vibronic coupling on the dipole strength of the 0–0 transition is included in the calculations.

## 5. Discussion

The Förster resonance energy transfer method based on the fragment molecular orbital and time-dependent density functional theory has been extended to describe excitonic couplings in solvent/protein environment that is approximated by a homogeneous continuum characterized by an optical dielectric constant.

By varying the calculation level, it is possible to extract valuable physical insight into the physicochemical factors affecting the excitonic coupling. Namely, from the difference between FMO0 and FMO1 results, one can evaluate the importance of the polarization of a chromophore by the other chromophores. From the difference between the results obtained from the transition density in vacuum and in protein/solvent environment, one can evaluate the importance of the polarization of the chromophores by the chromophore-induced dynamic environmental polarization (the reaction field effect). From the explicit contribution of the dynamic environmental polarization, one can evaluate the screening of the electrostatic coupling between the transition densities of the chromophores.

Key features of the different methods to calculate excitonic coupling here, are summarized in Table 9. The original Poisson-TrEsp method misses the reaction field enhancement of the dipole strength of the chromophores. The improved Poisson-TrEsp method includes this effect, using an average reaction field factor derived from FMO0/PCM[0] calculations. The latter method allows for a site-specific calculation of the reaction field enhancement. Finally, the FMO1/PCM[1] method includes the mutual polarization of ground state charge densities of the chromophores, that is neglected by the other three methods.

**Table 9:** Comparison of different methods for the calculation of excitonic couplings, with respect to treatment of screening effects, reaction field enhancement of the dipole strength and the mutual polarization between the ground states of the different chromophores

Method	Screening^a^	Reaction field^b^	Mutual polarization^c^
Poisson-TrEsp (original)	Yes	No	No
Poisson-TrEsp (improved)	Yes	Yes	No
FMO0/PCM[0]	Yes	Yes	No
FMO1/PCM[1]	Yes	Yes	Yes

a Explicit screening by the chromophore-protein/solvent electrostatic interaction.

b Implicit polarization of the chromophores by the protein/solvent environment.

c Mutual polarization of the chromophores.

The developed methods have been applied to the WSCP complex containing four chromophore units, in which electronic excitations are coupled. It has been shown that both the reaction field enhancement of the dipole strength of the chromophores as well as the explicit screening of the excitonic coupling are very important, whereas the site-dependence of the reaction field effect and the mutual polarization of electronic ground states are small effects. An improvement of the standard Poisson-TrEsp model has been suggested based on these findings, namely, to use a single scaling factor describing the polarization of the chromophore due to the reaction field, that was neglected before.

Note that the error compensation effect present in the description of the dipole strength with the empty spherical cavity model in Fig. 3, resulting from the assumption of a spherical cavity and the neglect of reaction field effects, is practically absent in the standard Poisson-TrEsp calculations, because a realistic molecular cavity is used in the latter. The only remaining relict of this error compensation is given by the calibration of the vacuum dipole strength of the chromophores with respect to the value *D*_0_ = 21.0 D^2^ obtained from the empty spherical cavity analysis of the experimental dipole strength that is slightly larger than the empirical value *D*_0_ = 20.2 D^2^. However, the ratio of these two values (1.04) is much smaller than the average reaction field factor *f*^rf^ = 1.44 resulting from the present FMO0/PCM[0] calculations. For complete error compensation these two factors should be equal.

One might wonder, if instead of going one step ahead in the theory and include reaction field effects in Poisson-TrEsp, it would also be possible to go one step back, rely again on error compensation effects, and use a spherical cavity in the calculation of the screening. In order to obtain an analytical estimate, we approximate the transition density of each chromophore *M* by a non-polarizable point dipole ***μ̃***^*M*^_0_ in the center of a spherical cavity with *ε* = 1 inside and *ε = n*^2^ outside the cavity. Neglecting the presence of the cavity of all other chromophores in the solution of the Poisson equation for the ESP *φ*^*M*^(**r**) of the Mth chromophore, results in^72^50
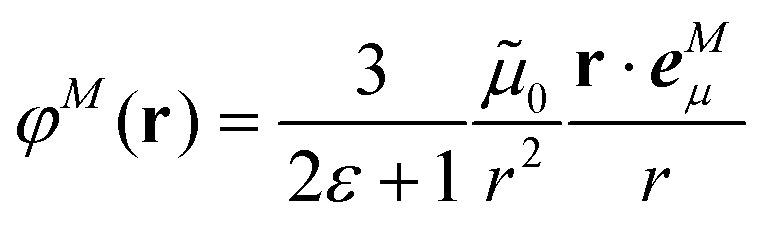
outside the cavity of this chromophore. Here, the origin of the coordinate system is in the center of the cavity and the transition dipole moment ***μ̃***^*M*^_0_ is expressed as *μ̃*_0_**e**^*M*^_*μ*_ using a unit vector **e**^*M*^_*μ*_. In the spirit of eqn (3) and (7), this ESP may be dissected,

51 *φ*^*M*^(**r**) = *φ*^*M*,0^(**r**) + *φ*^*M*,expl^(**r**),

into a vacuum contribution52
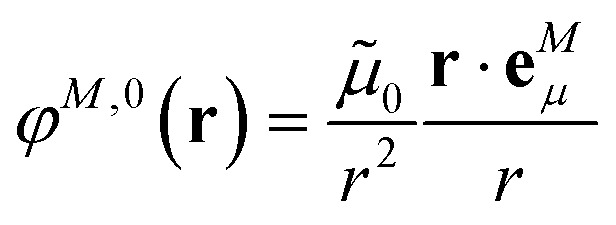
and an explicit solvent contribution53
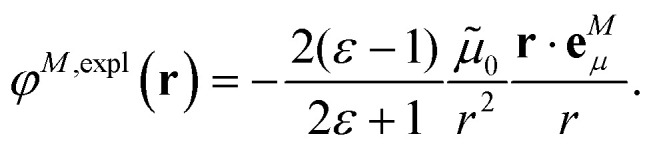
Hence, the explicit transition dipole moment of the solvent, induced by chromophore *M*, can be defined as54
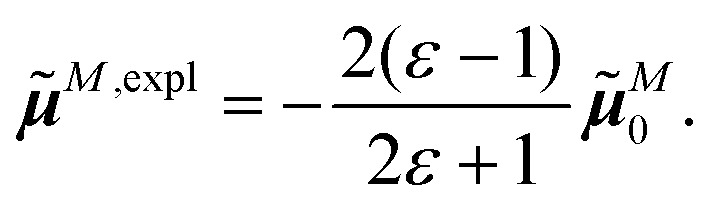
The excitonic interaction between chromophores *M* and *N* then is given as *V*^*μ*^_*MN*_ = *V*^0,*μ*^_*MN*_ + *V*^expl,*μ*^_*MN*_, where *V*^0,*μ*^_*MN*_ is the coupling between vacuum transition dipoles ***μ̃***^*M*^_0_ and ***μ̃***^*N*^_0_ and *V*^expl,*μ*^_*MN*_ is the coupling between the explicit transition dipole ***μ̃***^*M*,expl^ of the solvent (induced by chromophore *M*) and the vacuum transition dipole ***μ̃***^*N*^_0_ of chromophore *N*. With eqn (54), *V*^expl,*μ*^_*MN*_ can be expressed as55
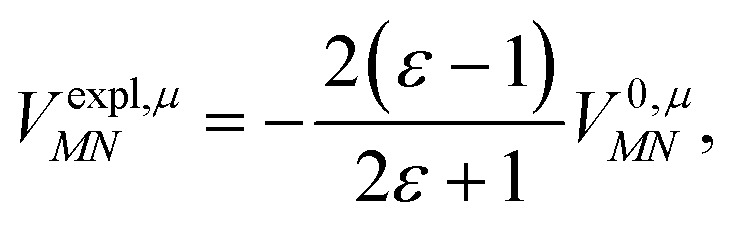
resulting in a uniform screening factor56
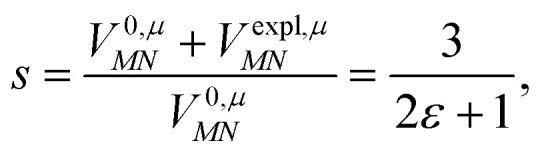
which for the present *ε* = 2 gives *s* = 0.6. Note that, the implicit contribution *V*^impl^_*MN*_ in eqn (10) corresponds to the vacuum coupling *V*^0,*μ*^_*MN*_ in eqn (56), since the transition dipoles in the present model are non-polarizable. This screening factor (eqn (56)) is in agreement with earlier work by Hsu *et al.*,^100^ who, in addition, investigated the screening factors of higher order multipoles. They found that the screening factor increases with the order of the multipole, reaching 2/(*ε* + 1) in infinite order. Using *ε* = 2 gives *s* = 0.66 in this limit. This range of screening factors of the analytical model agrees at least qualitatively with the values obtained numerically for realistic molecule-shaped cavities using the transition density (Table 2, *s*_*MN*_ = 0.60,…,0.71) or its point dipole approximation (PDA, Table 5, *s*_*MN*_ = 0.57,…,0.64). The analytical estimate explains why the *s* factors obtained in PDA are somewhat smaller than those resulting from the complete transition density.

Because the screening factors obtained in the analytical model and in the numerical calculations (Tables 2 and 5) are close, the assumption of a spherical cavity in the screening calculations cannot compensate for the neglect of the reaction field effects in the calculation of excitonic couplings. This kind of error compensation seems to be unique for the calculation of the dipole strength in different solvents (the blue line in Fig. 3).

Improving the Poisson-TrEsp method by including the reaction field effects, results in an average excitonic coupling of 85 cm^−1^ between the strongly coupled Chl *a* chromophores in WSCP providing an excellent agreement between calculated and experimental linear absorption and circular dichroism spectra (Fig. 6). Can this reaction field factor be applied to other antenna complexes containing Chl *a* chromophores? What are the limitations and uncertainties of the present estimate of *f*^rf^ and calibration of the excitonic couplings? These and related questions are discussed in the following.

Our average reaction field factor *f*^rf^ = 1.44 neglects any qualitative change of the transition density of the chromophore by the reaction field of the protein/solvent environment, not described by a single scaling factor. The present analysis of the transition dipole moments of the chromophores and of the electrostatic potential of the reaction field contribution to the transition density (Fig. 5b) indeed show that the major effect of the environment is on the magnitude and not on the direction of the transition dipole. Further support is obtained from the perfect correlation between the ESP of the transition density of the chromophores calculated in vacuum and in the dielectric environment (Fig. S3, ESI†). Consequently, the calibrated Poisson-TrEsp couplings (neglecting the change in shape of the transition density) and the calibrated FMO/PCM couplings (taking into account a change in shape of the transition density) agree very well. From the present FMO0/PCM[0] calculations of the excitonic couplings between the 4 Chl *a* chromophores the reaction field factors are all within a few percent, suggesting that it is the immediate pigment environment that has the strongest influence. Indeed, a very similar reaction field factor *f*^rf^ = 1.46 is obtained, if instead of all pigment subcavities just the one of the pigments, for which the transition density is calculated, is included. This result is in agreement with independent PCM calculations on a different system (the reaction center of photosystem II).^39^ As seen in Fig. S1 (ESI†), the ratio between the dipole strength obtained by including just the subcavity of one pigment and that by including also that of the other, varies by at most 8 percent between short and long inter-pigment distances.

As noted before, by calibrating the Poisson-TrEsp as well as the FMO[0]/PCM[0] couplings, according to the experimental vacuum dipole strength of the chromophore we implicitly assume that the quantum chemical vacuum transition dipole moment is not too far from the experimental value. Since the reaction field factor is determined in a self-consistent way in PCM calculations, the reaction field can, in principle, depend in a non-linear way on the transition density of the chromophore, which limits a linear correction to small differences in the transition densities. In order to compare the calculated and measured transition dipole moments, we also have to take into account that the experimental value refers to the 0–0 transition of Chl *a*, that is, the transition that does not involve the excitation of high-frequency intramolecular vibrational modes. In the Condon approximation,^1^ the transition dipole moment of the 0–0 transition is related to the full dipole moment by a factor exp(−*S*/2) with the total Huang–Rhys factor *S* (eqn (33)).

The relevant quantum-mechanical dipole strength *D*^(0–0)^_0_ of the 0–0 transitions is related to the calculated vacuum dipole moment magnitude by

57 *D*^(0–0)^_0_ = *e*^−*S*^*μ*_0_^2^.

With the average vacuum dipole moment magnitude of 5.34 D (Table 1) and the total Huang–Rhys factor *S* = 0.23 of the high-frequency intramolecular modes of Chl *a* in WSCP, determined above, a quantum chemical vacuum dipole strength of the 0–0 transition *D*^(0–0)^_0_ = 22.7 D^2^ results, which is reasonably close to the experimental value (*D*^(0–0)^_exp_ = 20.2 D^2^) to justify a linear correction. A certain robustness of this calibration procedure is seen in the improved Poisson-TrEsp method, where the transition charges of non-hydrogen atoms, obtained earlier^71^ from a fit of the ESP of the transition density of isolated Chl *a*, calculated with TDDFT/B3LYP are placed on the respective atom positions of the chromophores in the crystal structure of WSCP. The average dipole strength obtained in this way for the 4 chromophores is 25.9 D^2^, which deviates somewhat stronger from the experimental value than the FMO0/PCM[0]/CAM-B3LYP value. Nevertheless, the calibrated excitonic couplings, obtained with the two methods, are very close.

In order to investigate the robustness of this calibration procedure further, we performed additional FMO[0]/PCM[0] calculations, where the excited state calculations were performed with CIS (configuration interaction with single excitations) using molecular orbitals from Hartree–Fock (HF/CIS) and with TDDFT/B3LYP calculations, in order to compare these results with those obtained above using TDDFT/CAM-B3LYP. In case of HF/CIS, an average vacuum transition dipole moment of 6.23 D is obtained (for individual values see Table S12, ESI†) that gives rise to a 0–0 transition dipole strength of 30.8 D^2^. This value is considerably larger than the experimental value (20.2 D^2^). Nevertheless, the majority of the calibrated excitonic couplings is within 10% of the corresponding TDDFT/CAM-B3LYP values (Table 10, 3rd and 6th columns). The largest deviations of 20% are obtained for the intermediate couplings. The smaller values of the HF/CIS couplings reflect the smaller reaction field effect obtained with this method (Fig. 3). An estimation of the cavity field effect with PCM calculations could help to improve the description of the experimental dipole strengths in Fig. 3 and on this basis decide which of the two descriptions (TDDFT or HF/CIS) is more realistic. In case of TDDFT/B3LYP the deviations with respect to the TDDFT/CAM-B3LYP values are less than 10% for all couplings (Table 10, 3rd and 5th columns).

**Table 10:** Effect of different quantum chemical methods and geometry optimization schemes on the calibrated couplings (cm^−1^)

*M*	*N*	CAM-B3LYP^a^	CAM-B3LYP^b^	B3LYP^c^	HF/CIS^d^	HF/CIS^e^
3	4	92	84	93	85	91
1	2	90	85	91	83	92
2	3	21	21	23	17	15
1	4	20	20	21	16	14
2	4	7	7	6	6	7
1	3	6	7	6	5	5

a Same as in 3rd column of Table 8.

b Using a different optimized geometry based on dimer calculations (coordinates are given in the ESI, Table S3), as described in the methods section, original data are given in the ESI, Tables S4 and S5.

c Full data are given in Tables S14 and S15 (ESI).

d Full data are given in Tables S12 and S13 (ESI).

e Using a different geometry optimization (coordinates are given in Table S16, ESI), based on HF, original data are given in Tables S17 and S18 (ESI).

If the geometry optimization, performed above with DFT/CAM-B3LYP, is instead done with HF, the HF/CIS calculations result in calibrated excitonic couplings that in the majority of cases are even closer to the TDDFT/CAM-B3LYP results (Table 10, 3rd and 7th columns). If rather than a monomer, a dimer optimization with DFT/CAM-B3LYP is used, as described in the methods section, the resulting calibrated TDDFT/CAM-B3LYP couplings are all within 10% of the original TDDFT/CAM-B3LYP couplings (Table 10, 3rd and 4th columns). These results on one hand justify our linear scaling procedure and on the other hand they demonstrate a great robustness of our methods with respect to the details of the quantum chemical method used. Note that, besides the redistribution of oscillator strength discussed in Section 2.7, there are additional subtleties as a Dushinsky rotation of normal modes,^101^ Jahn–Teller corrections^102^ to the Condon approximation that have an influence on the exact value of the (effective) Huang–Rhys factor. However, these additional effects are small compared to the 3.5 fold correction found above (eqn (44)).

Our estimates rely on the validity of the PCM model, that describes the solvent and protein environment by a simple homogeneous dielectric continuum. One way to check this approximation is to include the heterogeneous polarizability of the protein in the calculations. QM/MMPol calculations that take into account the chromophores on a quantum mechanical level and the protein/solvent environment by a classical polarizable force field, revealed a somewhat larger variation of the screening factor but a similar average value as PCM calculations for the excitonic couplings in a light-harvesting antenna of cryptophytes.^103^ As for the present system, screening factors smaller than one were obtained for cryptohytes. A detailed investigation of the contribution of environmental molecules to the screening factor of excitonic couplings in the LH2 light-harvesting complex of purple bacteria with QM/MMPol^104^ revealed that there can be parts of the environment that enhance the excitonic coupling and other parts that decrease it. The latter seem to prevail so that overall the excitonic coupling gets smaller, in agreement with the present and our earlier^43^ calculations which use a continuum description of the environment. Note, however, that in very few cases an enhancement was obtained.^43^

As discussed in the introduction, the QM/MMPol method was also applied to WSCP. This complex has the advantage that the largest coupling can be directly estimated from the splitting between the optical transitions in the spectra, as discussed above. Rosnik and Curutchet^52^ report that the average transition dipole moment magnitude of 8.75 D for the Chl a pigments in WSCP in their calculations includes a 25% increase by the protein/solvent environment. Hence, we obtain that the magnitude of the average vacuum transition dipole moment in their calculation is equal to 7.00 D. Using this value for *μ̃*^*M*^_0_ and *μ̃*^*N*^_0_ in eqn (32) together with the experimental vacuum dipole strength *D*_0_ = 20.2 D^2^ and the original average intradimer coupling^52^ (*V*_12_ + *V*_34_)/2 = 186 cm^−1^ results in a calibrated average coupling (*Ṽ*_12_ + *Ṽ*_34_)/2 = 76 cm^−1^, which is now within a 10 percent margin of the coupling obtained with the improved Poisson-TrEsp method that describes the optical spectra very well (Fig. 6).

The individual QM/MMPol couplings are given in the last column of Table 8. Let us have a look at the physical origin of the calibration factor, used above, that brought the *ab initio* coupling value of the QM/MMPol method so close to the experimental estimate. One part of the calibration is due to the renormalization of the excitonic coupling by the vibronic coupling. Using the total Huang–Rhys factor of the high-frequency intramolecular modes *S* = 0.23, discussed in Section 2.7, the overall excitonic coupling (*V*_12_ + *V*_34_)/2 = 186 cm^−1^ is reduced by a factor *e*^−*S*^ = 0.79 to yield a coupling of 148 cm^−1^ between 0–0 transitions of the chromophores. The remaining deviation is due to the limitations of the semi-empirical Zerner's intermediate neglect of differential overlap (ZINDO) method used in the quantum-chemical calculations, which results in a vacuum transition dipole strength of the 0–0 transition of 49 D^2^ × *e*^−*S*^ = 39 D^2^ (eqn (57)), which is almost twice as large as the experimental value *D*_0_ = 20.2 D^2^. Therefore, the linear calibration of the excitonic coupling (eqn (32)) becomes questionable and the good agreement of the calibrated coupling with the experimental estimate appears somewhat fortuitous.

Interestingly, the calibrated QM/MMPol couplings between chromophores 2 and 3 and between 1 and 4 are 30% smaller than the respective Poisson-TrEsp and FMO0/PCM[0] values, an effect that could be caused by the heterogeneous polarizability of the environment (an advantage of QM/MMPol). The 50% variation between the two smallest QM/MMPol couplings (1–3 and 2–4) implies that the *D*_2_ symmetry of the complex was broken in the MD simulations. The QM/MMPol calculations took into account protein-induced distortions of the pigments that should affect their excitonic couplings. In addition to the excitonic couplings, the spectral density of the exciton-vibrational coupling has been calculated in the QM/MMPol study^52^ and good agreement with the spectral density extracted by Pieper *et al.* from their experimental *Δ*-FLN spectra,^49^ using the standard electronic two state approach, was reported. The present 3.5 fold correction of the intramolecular Huang–Rhys factor of the chromophores (see Section 2.7) suggests that the above agreement is not as good as it was thought.^52^

The present analysis shows that the mutual polarization of the ground states of the chromophore, included in FMO1/PCM[1] and neglected in FMO0/PCM[0] has only a very minor effect on the excitonic couplings in WSCP. This result is consistent with the fact that the excitonic couplings obtained for different static dielectric constants are practically identical (Table S19, ESI†).

It might be surprising at first glance that the FMO-TDDFT/PCM calculations and the Poisson-TrEsp calculations practically obtain identical screening factors of the excitonic interaction. As noted above, both calculations rely on a perturbative treatment of the screening and on classical electrostatics,^43,67,100^ solving the Poisson equation for the ESP of the transition density of the chromophores that are surrounded by a dielectric continuum with optical dielectric constant *ε*. In PCM a boundary element method is used for the solution of the Poisson equation, which allows one to represent the influence of the solvent by apparent surface charges on the chromophore subcavities. These surface charges are self-consistently determined with the transition density that polarizes the dielectric. Using perturbation theory,^67,100^ the overall Coulomb coupling is dissected into a direct interaction term that contains the influence of the solvent on the transition density and an indirect (screening) part that contains the Coulomb interaction of the solvent polarization induced by the transition density of one chromophore with the transition density of the other chromophore.

In Poisson-TrEsp,^42,43^ the Poisson equation is solved by a finite difference method revealing the overall ESP of the transition density of the chromophores that is used to calculate the overall excitonic coupling. By comparing this coupling with the vacuum coupling, the screening factor is obtained. In PCM this factor results from comparing the excitonic coupling with and without the explicit contribution of the solvent, but taking into account the implicit contribution of the solvent to the transition density of the chromophores. Since, however, the Poisson equation is linear in the transition density, the implicit contribution of the solvent, present in PCM and absent in Poisson-TrEsp, cancels out in the ratio of electrostatic couplings that defines the screening factor, as long as the transition densities with and without implicit environmental contribution differ only by a scalar constant. Therefore, both methods give the same value for the screening factor. Hence, Poisson-TrEsp is fully capable to resolve the slight rotation of the screened transition dipole moment seen in Fig. 5d.

There is a long-standing controversy concerning the distance dependence of the screening factor obtained with HF/CIS/PCM^38,39^ and Poisson-TrEsp calculations.^43^ Now that we know that Poisson-TrEsp and PCM-based methods should give almost identical screening factors, this controversy can be discussed in a more stringent manner. Whereas the PCM-based calculations on D1D2cytb559 complexes of photosystem II (containing 6 chlorophyll *a* and 2 pheophytin *a* pigments)^38,39^ reported an exponential distance dependence of the screening factor, the Poisson-TrEsp calculations on photosystem I trimers (containing 288 chlorophyll *a* pigments) suggested no systematic distance dependence.^43^ Instead, the screening factor was found to depend on the mutual orientation of the pigments. The present results are in agreement with this finding (see the screening factors in Table 2 and the distances in Table S1, ESI†). Chromophore pairs 1–2 and 3–4 with the smallest interchromophore distance (10 Å) have a larger screening factor than 2–3 and 1–4 and a smaller one than 2–4 and 1–3, although all the latter 4 pairs have a much larger pigment–pigment distance (20–21 Å). It seems that, either the number of pigment pairs in the PCM study^38,39^ was simply too small to draw any general conclusions, or the different assignment of the dielectric environment in the two methods is responsible for the different distance dependencies found. Whereas in the HF/CIS/PCM^38,39^ study the subcavities were just assigned to the two pigments for which the interaction was calculated, in the Poisson-TrEsp study^43^ all pigment subcavities were considered simultaneously, as done in the present study. Which of the two assigments is more realistic still has to be evaluated. If two pigments interact, the remaining pigments can be seen as polarizable environment. However, part of this polarization (the resonant transition) is explicitly included in the exciton Hamiltonian (eqn (22)). Hence, the polarization of the pigments *via* the remaining high-energy transitions has to be evaluated.

## 6. Conclusions

The polarizable continuum model (PCM) for the calculation of excitonic couplings in solvent/protein environments was implemented in the fragment molecular orbital (FMO) method and used for an in-depth analysis of the excitonic couplings in the water soluble chlorophyll binding protein WSCP.

The previous QM/MMPol study^52^ of WSCP could not provide an explanation for the excitonic coupling in the Chl dimers of WSCP. In this work, the FMO/PCM method has been used to investigate this question. An important aspect of the analysis is to recognize that the splitting between the two low-energy exciton peaks in the experiment is determined by the excitonic coupling between the 0–0 transitions of the Chls. The dipole strength of the latter was inferred^60^ from experimental oscillator strengths in different solvents. Adjusting the quantum chemical transition density of the chromophores such that without surrounding medium, the experimental vacuum transition dipole moment of the 0–0 transition results, gives an excitonic coupling in the Chl *a* dimers of WSCP that is within 10% of the experimental estimate, not only for our FMO/PCM calculations and the improved Poisson-TrEsp method but also for the QM/MMPol value,^52^ providing excellent agreement between calculated and experimental optical spectra.

Our scaling in the calibrated excitonic couplings is robust against variations of the details of the quantum chemical method, as demonstrated by using different functionals in the TDDFT calculations and the HF/CIS method, different geometry optimizations (Table 10), as well as heavy atom transition charges^71^ and crystal-structure chromophore geometries used in the original and the present improved Poisson-TrEsp methods. Whereas previously^59^ the excitonic coupling in WSCP had to be treated as a free parameter, in this work, a structure-based calculation of this coupling has become possible. The coupling obtained with the original Poisson-TrEsp method is too small to reveal the full signature of the low-energy exciton state, which is hidden under the main absorption peak dominated by the high-energy exciton state (Fig. 6).

A detailed analysis of the FMO/PCM calculations reveals that the enhancement of the dipole strength of the chromophores by the polarization of the solvent/protein environment of one chromophore is rather insensitive to the presence of the other chromophores and that the main effect of the reaction field is indeed just a scalar amplification of the transition dipole moment. The screening part of the FMO/PCM calculations can be described quantitatively by the electrostatic Poisson-TrEsp method. The present results suggest a new calibration scheme for the atomic transition charges used in Poisson-TrEsp calculations. This new scheme takes into account reaction field effects by an average reaction field factor determined with FMO0/PCM[0] calculations. Together with the experimental vacuum dipole strength^60^*D*_0_ = 20.2 D^2^, this factor results in an effective transition dipole moment magnitude *μ*_P-TrEsp_ = 5.39 D to which the Poisson-TrEsp transition charges are to be scaled in the calculation of screening part of the coupling. Because the screening part of the coupling involves the solution of the Poisson equation that is linear in the transition charges, the previous Poisson-TrEsp couplings,^105–108^ that were obtained with transition charges scaled to a dipole moment squared equal to 21.0 D^2^ (obtained from an empty spherical cavity analysis of experimental dipole strengths), need to be multiplied by a factor (5.39 D)^2^/21.0 D^2^ = 1.38. Please note that this calibration factor contains the average reaction field factor, obtained by the present FMO0/PCM[0]/CAM-B3LYP calculations and a correction for the different vacuum dipole strengths used in the original and the improved Poisson-TrEsp method. In case of WSCP, the improved Poisson-TrEsp method leads to a significantly better agreement between calculated and experimental optical spectra than the original method (Fig. 6). We expect a similar improvement for the optical spectra and energy transfer calculations of other pigment–protein complexes containing Chl *a* chromophores.

The scaling factor of the improved Poisson-TrEsp method uses only the outcome from theoretical calculations (FMO-TDDFT/PCM) and a single experimental value of the vacuum dipole strength of the 0–0 transition of the pigments, that can be extrapolated from experimental values of the oscillator strength of a given pigment measured in solvents with different refractive index. Note that, such experimental data and extrapolations are available also for some other photosynthetic pigments (Chl *b*, bacteriochlorophyll *a* (BChl *a*), BChl *c*),^60^ but with somewhat larger uncertainty than for Chl *a.* Hence, additional experiments for these and other pigments would be helpful. Besides the molecular structure and the vacuum dipole strength of the pigments, no other experimental input is needed to accurately predict the excitonic couplings with the present methods.

## Conflicts of interest

There are no conflicts to declare.

## Supplementary Material

CP-024-D1CP03566E-s001
