# Supplementary material for: Towards a quantitative description of excitonic couplings in photosynthetic pigment–protein complexes: quantum chemistry driven multiscale approaches
Source: Phys Chem Chem Phys. 2022 Jan 3;24(8):5014–38. doi: 10.1039/d1cp03566e (PMC8865841; doi:10.1039/d1cp03566e)
Supplement: CP-024-D1CP03566E-s001 [file CP-024-D1CP03566E-s001.pdf]

Electronic Supplementary Material (ESI) for Physical Chemistry Chemical Physics.
This journal is © the Owner Societies 2022

Supporting Information

Towards a quantitative description of excitonic couplings in photosynthetic pigment-protein complexes: quantum chemistry driven multiscale approaches

Christian Friedl,[1] Dmitri G. Fedorov,[2,*] Thomas Renger[1,*]

[1] Institut für Theoretische Physik, Johannes Kepler Universität Linz, Altenberger Str. 69, 4040 Linz, Austria

[2] Research Center for Computational Design of Advanced Functional Materials (CD-FMat), National Institute of Advanced Industrial Science and Technology (AIST), Central 2, Umezono 1-1-1, Tsukuba, 305-8568, Japan.

* Corresponding author: E-mail address: d.g.fedorov@aist.go.jp (D.G. Fedorov).

* Corresponding author: E-mail address: thomas.renger@jku.at (T. Renger).

Download links for the MEAD program

MEAD was formerly hosted on **http://www.scripps.edu/bashford** and later on **http://stjudere-search.org/site/lab/bashford/**. On both of these sites MEAD is no longer available. The Internet Archive shows that there was a form on the latter site where one could request a copy of MEAD.
https://web.archive.org/web/20160529023011/http://stjuderesearch.org/site/lab/bashford/
The GNU General Public License under which MEAD was distributed allows its free redistribution. It is available, e.g., also via the following link: **https://github.com/friedc/MEAD_2.2.9**

M	***N***	***d*** **(Å)**	ϑ_M (°)	φ_M (°)	ϑ_N (°)	φ_N (°)
3	4	9.807	-59.6	-57.0	54.3	-59.0
1	2	9.829	-59.5	-61.4	53.7	-59.4
2	3	19.203	-22.4	1.7	23.5	-1.1
1	4	19.352	-22.2	-0.2	24.5	-0.9
2	4	20.817	-26.9	32.1	29.6	30.2
1	3	21.194	-29.0	29.5	30.8	28.9

Table S1: Distances and orientations of pigment pairs in WSCP (for structure see Fig. 1 of the main text). The distance $d=|\boldsymbol{R}^M-\boldsymbol{R}^N|$ between chromophores M and N is measured between the geometric centers of the nitrogen atoms NA, NB, NC, ND of each chromophore respectively (illustrated in Fig. 4 of the main text). ϑ_M is the angle between the distance vector $\boldsymbol{R}^M-\boldsymbol{R}^N$ and the XY plane of chromophore M. φ_M is the angle in the XY plane between the distance vector and the Y direction of chromophore M. ϑ_N and φ_N are defined accordingly for chromophore N.

Atomic coordinates, numbering scheme and pigment alignment

Pigment No	Atom No	Atom Name	Coordinates			Vacuum TrESP Charge	Solvent TrESP Charge
I	*M*		X (Å)	Y (Å)	Z (Å)	$\widetilde{q}_{\alpha}^{M}$ (e)	$\widetilde{q}_{\alpha}^{M}$ (e)
1	1	MG	12.32543155	40.67719294	-4.52312936	-0.0090	-0.0107
1	2	CHA	13.49155542	43.17315986	-6.57991196	0.1411	0.1369
1	3	CHB	14.69984661	41.49777587	-2.22475522	-0.0544	-0.0395
1	4	CHC	11.15925337	38.21592356	-2.50094710	-0.1710	-0.1706
1	5	CHD	9.95982478	39.85097507	-6.91920091	0.0652	0.0470
1	6	NA	13.82715323	42.16734484	-4.42050006	0.0128	0.0136
1	7	C1A	14.14237972	43.11277535	-5.37830452	-0.0974	-0.0934
1	8	C2A	15.26777746	44.01662568	-4.91162684	-0.1174	-0.1069
1	9	C3A	15.75394503	43.31655217	-3.62813841	0.0838	0.0726
1	10	C4A	14.69347340	42.25596910	-3.38537278	0.1005	0.0996
1	11	CMA	17.15676470	42.71666234	-3.76677357	-0.0290	-0.0367
1	12	CAA	14.78891363	45.45698887	-4.66269829	0.0773	0.0714
1	13	CBA	15.91253194	46.43214120	-4.27135569	-0.0073	-0.0039
1	14	CGA	16.87199144	46.68156859	-5.41145272	0.0090	0.0094
1	15	O1A	16.75934185	47.56051486	-6.22818281	-0.0006	-0.0009
1	16	O2A	17.87369424	45.78095802	-5.42223067	-0.0085	-0.0093
1	17	NB	12.87115822	39.96508859	-2.69739738	-0.0707	-0.0682
1	18	C1B	13.84819345	40.43299371	-1.89471165	0.0554	0.0520
1	19	C2B	13.89523528	39.66323008	-0.66323422	0.0209	0.0214
1	20	C3B	12.92360577	38.70886175	-0.75906756	0.0102	0.0123
1	21	C4B	12.25483947	38.91179712	-2.04441746	0.1319	0.1372
1	22	CMB	14.86870286	39.88784809	0.44780111	-0.0230	-0.0246
1	23	CAB	12.61633631	37.68663679	0.24322192	-0.0216	-0.0231
1	24	CBB	12.29179847	36.41585908	0.00293219	0.0218	0.0231

Pigment No	Atom No	Atom Name	Coordinates			Vacuum TrESP Charge	Solvent TrESP Charge
1	25	NC	10.84980043	39.24645431	-4.69528377	0.0020	0.0020
1	26	C1C	10.48560370	38.36055701	-3.74060717	0.1389	0.1403
1	27	C2C	9.33903125	37.60223305	-4.16539928	-0.0324	-0.0243
1	28	C3C	9.01331225	38.06064647	-5.41840516	0.0569	0.0525
1	29	C4C	9.96363881	39.09775252	-5.73987929	-0.0892	-0.0810
1	30	CMC	8.65581547	36.53892033	-3.36247590	-0.0134	-0.0182
1	31	CAC	7.91237362	37.57569586	-6.31562949	-0.0282	-0.0286
1	32	CBC	8.35975004	36.45423994	-7.26062071	0.0152	0.0160
1	33	ND	11.78403841	41.33697265	-6.35213516	0.0622	0.0594
1	34	C1D	10.81105227	40.90216857	-7.24202530	-0.0690	-0.0593
1	35	C2D	10.85917086	41.68890921	-8.45022191	-0.0482	-0.0583
1	36	C3D	11.89460583	42.58680478	-8.24803837	-0.0013	-0.0052
1	37	C4D	12.41968718	42.32747289	-6.96184876	-0.1034	-0.1007
1	38	CMD	9.98594185	41.54971393	-9.65402226	0.0532	0.0633
1	39	CAD	12.65220327	43.68495358	-8.84142588	0.0087	0.0159
1	40	OBD	12.53235957	44.22987038	-9.91582889	-0.0224	-0.0358
1	41	CBD	13.73582082	44.09627308	-7.77798119	-0.0955	-0.1082
1	42	CGD	15.14321840	43.94375987	-8.31718965	0.0289	0.0337
1	43	O1D	16.06294433	44.68753825	-8.06070335	-0.0042	-0.0055
1	44	O2D	15.27282358	42.84895990	-9.07414446	-0.0002	0.0042
1	45	CED	16.58362926	42.60323569	-9.58430219	0.0009	-0.0076
1	46	C1	18.76417192	45.87630622	-6.54086449	0.0105	0.0111
1	47	H	15.46320839	41.75305990	-1.49758020	0.0003	-0.0024
1	48	H	10.75683550	37.46876657	-1.82727062	0.0211	0.0194
1	49	H	9.20722262	39.58810756	-7.65640656	-0.0011	0.0028
1	50	H	17.88067536	43.51158470	-3.96657301	0.0136	0.0159
1	51	H	17.19171241	42.00632871	-4.59924265	0.0077	0.0101

Pigment No	Atom No	Atom Name	Coordinates			Vacuum TrESP Charge	Solvent TrESP Charge
1	52	H	17.45829080	42.18445720	-2.86003995	-0.0015	0.0031
1	53	H	14.03976673	45.45091243	-3.86263894	-0.0168	-0.0156
1	54	H	14.29232890	45.84615841	-5.55618120	-0.0176	-0.0164
1	55	H	15.47176981	47.39624412	-4.00836774	-0.0048	-0.0056
1	56	H	16.46881500	46.05902447	-3.40636643	-0.0055	-0.0062
1	57	H	14.76313730	40.88952353	0.87981663	0.0151	0.0173
1	58	H	15.90257498	39.79651951	0.09618438	0.0135	0.0149
1	59	H	14.73350609	39.16000508	1.25063745	0.0081	0.0096
1	60	H	12.69659734	38.00810018	1.28046792	0.0063	0.0072
1	61	H	12.24198546	36.00998758	-1.00228067	0.0007	-0.0002
1	62	H	12.09025870	35.72928977	0.81870286	0.0047	0.0061
1	63	H	7.86395231	36.05325364	-3.93767163	0.0046	0.0054
1	64	H	8.19960460	36.94974907	-2.45454674	0.0028	0.0049
1	65	H	9.35508547	35.75712666	-3.04646478	0.0022	0.0047
1	66	H	7.07355425	37.22322340	-5.70590019	0.0064	0.0076
1	67	H	7.51614705	38.40990320	-6.90482828	0.0095	0.0088
1	68	H	8.71636750	35.58837064	-6.69436338	-0.0042	-0.0041
1	69	H	9.18069594	36.78602273	-7.90353107	-0.0007	-0.0014
1	70	H	7.53576648	36.12542829	-7.90178375	0.0012	0.0012
1	71	H	10.22361920	42.32709539	-10.38284386	-0.0147	-0.0182
1	72	H	8.92559241	41.63447035	-9.39292887	-0.0264	-0.0344
1	73	H	10.12484416	40.57732216	-10.13964449	-0.0237	-0.0270
1	74	H	13.60717580	45.15610948	-7.54276578	0.0271	0.0279
1	75	H	16.50148375	41.70002176	-10.18671725	-0.0001	0.0015
1	76	H	17.28885229	42.45451250	-8.76364678	-0.0052	-0.0031
1	77	H	16.92001127	43.44354878	-10.19440075	-0.0021	-0.0012
1	78	H	19.56103882	45.16016940	-6.34242863	-0.0026	-0.0023

Pigment No	Atom No	Atom Name	Coordinates			Vacuum TrESP Charge	Solvent TrESP Charge
1	79	H	19.16595062	46.88776840	-6.62023266	-0.0038	-0.0042
1	80	H	18.22772841	45.61995251	-7.45537076	-0.0022	-0.0025
1	81	H	16.06066437	44.04237204	-5.66225713	0.0168	0.0148
1	82	H	15.75897804	44.01495140	-2.78318128	-0.0187	-0.0131
2	1	MG	20.33200762	39.26504377	-10.09126813	0.0024	0.0022
2	2	CHA	17.13598902	38.39744528	-11.01223274	0.1130	0.1015
2	3	CHB	20.53515392	36.44366588	-8.21124079	-0.0395	-0.0262
2	4	CHC	23.50439178	40.13976673	-9.19821393	-0.1692	-0.1719
2	5	CHD	20.07931956	42.12143116	-12.03726487	0.0776	0.0591
2	6	NA	19.02590979	37.67037485	-9.68173250	0.0272	0.0257
2	7	C1A	17.76794445	37.48010894	-10.21912642	-0.1160	-0.1078
2	8	C2A	17.24217326	36.10360363	-9.87973499	-0.0461	-0.0367
2	9	C3A	18.14076168	35.70209666	-8.69460761	0.0444	0.0345
2	10	C4A	19.33229422	36.63443302	-8.86732471	0.0794	0.0797
2	11	CMA	17.47501471	35.92934163	-7.33372152	-0.0284	-0.0349
2	12	CAA	17.39901248	35.16119234	-11.09352836	0.0542	0.0549
2	13	CBA	16.46695194	33.94944816	-11.02264080	-0.0286	-0.0334
2	14	CGA	15.04944083	34.37514934	-11.33979230	0.0156	0.0188
2	15	O1A	14.73647929	34.99321493	-12.33142570	-0.0033	-0.0039
2	16	O2A	14.17973131	33.99928989	-10.39672769	-0.0033	-0.0042
2	17	NB	21.76511495	38.45660351	-8.89190231	-0.0824	-0.0840
2	18	C1B	21.66413585	37.28671722	-8.22427398	0.0600	0.0598
2	19	C2B	22.89286916	37.02546424	-7.49515130	0.0246	0.0260
2	20	C3B	23.72154078	38.08767013	-7.72262512	0.0181	0.0183
2	21	C4B	23.01473102	38.98570222	-8.63314749	0.1328	0.1422
2	22	CMB	23.13276391	35.83118130	-6.62848422	-0.0454	-0.0494
2	23	CAB	25.05521633	38.27797832	-7.15089376	-0.0365	-0.0380

Pigment No	Atom No	Atom Name	Coordinates			Vacuum TrESP Charge	Solvent TrESP Charge
2	24	CBB	25.56411002	39.43083199	-6.71537005	0.0337	0.0359
2	25	NC	21.57405752	40.84621932	-10.54947548	-0.0158	-0.0174
2	26	C1C	22.83656301	41.01962730	-10.08733452	0.1583	0.1619
2	27	C2C	23.40112919	42.23208173	-10.61766696	-0.0464	-0.0379
2	28	C3C	22.43678758	42.79543841	-11.41803595	0.0561	0.0519
2	29	C4C	21.29372482	41.91881546	-11.36581798	-0.0938	-0.0854
2	30	CMC	24.77357290	42.75099095	-10.32009950	0.0093	0.0030
2	31	CAC	22.51404821	44.09190199	-12.16863985	-0.0304	-0.0298
2	32	CBC	22.02717642	45.29258889	-11.34757036	0.0129	0.0134
2	33	ND	18.92553445	40.13517839	-11.24399086	0.0555	0.0541
2	34	C1D	18.95642500	41.30176716	-12.00083384	-0.0601	-0.0512
2	35	C2D	17.70117737	41.46420410	-12.70147013	-0.0632	-0.0776
2	36	C3D	16.94345726	40.36660123	-12.33412928	0.0100	0.0141
2	37	C4D	17.73419728	39.59888290	-11.45124707	-0.0976	-0.0963
2	38	CMD	17.31304237	42.58227292	-13.61162756	0.0610	0.0697
2	39	CAD	15.67202146	39.66925617	-12.52714622	-0.0085	-0.0083
2	40	OBD	14.72562116	39.95510855	-13.22522371	-0.0209	-0.0331
2	41	CBD	15.73132134	38.38018053	-11.62322535	-0.0430	-0.0440
2	42	CGD	14.68220542	38.43535555	-10.53018636	0.0161	0.0182
2	43	O1D	14.08742341	37.47948519	-10.08536455	-0.0066	-0.0085
2	44	O2D	14.52238494	39.67826786	-10.06018732	-0.0023	0.0005
2	45	CED	13.57783109	39.82064461	-8.99885607	0.0046	-0.0002
2	46	C1	12.82959977	34.43259948	-10.60603318	-0.0012	0.0003
2	47	H	20.61336489	35.55136407	-7.59985584	-0.0009	-0.0030
2	48	H	24.52158618	40.40771998	-8.93958889	0.0193	0.0178
2	49	H	20.00981373	43.01013962	-12.65691586	-0.0055	-0.0011
2	50	H	16.59862762	35.28393044	-7.21884953	0.0141	0.0164

Pigment No	Atom No	Atom Name	Coordinates			Vacuum TrESP Charge	Solvent TrESP Charge
2	51	H	17.14416276	36.96837386	-7.23587493	0.0092	0.0104
2	52	H	18.16943961	35.71819093	-6.51523463	0.0009	0.0048
2	53	H	17.17603776	35.69735084	-12.01918524	-0.0098	-0.0119
2	54	H	18.44270714	34.83419710	-11.16058058	-0.0142	-0.0126
2	55	H	16.50495441	33.46106733	-10.04575557	0.0018	0.0029
2	56	H	16.76092322	33.21452266	-11.77947466	0.0007	0.0015
2	57	H	22.89539602	34.89983311	-7.15284324	0.0195	0.0220
2	58	H	22.51139864	35.86165198	-5.72547552	0.0186	0.0205
2	59	H	24.17380713	35.77509820	-6.30235268	0.0153	0.0176
2	60	H	25.66027962	37.37822086	-7.04912854	0.0102	0.0113
2	61	H	24.99801360	40.35614927	-6.73905918	-0.0030	-0.0044
2	62	H	26.56373612	39.47754025	-6.29549600	0.0028	0.0039
2	63	H	25.01945690	43.60369579	-10.95738616	-0.0013	0.0000
2	64	H	25.54058620	41.98658491	-10.48453655	-0.0037	-0.0008
2	65	H	24.86719982	43.08334480	-9.27955543	-0.0032	-0.0005
2	66	H	21.92815291	44.02175433	-13.09185976	0.0111	0.0099
2	67	H	23.54651206	44.26968594	-12.48816226	0.0081	0.0089
2	68	H	20.99792864	45.14239302	-11.00807700	0.0002	0.0002
2	69	H	22.06041539	46.21207062	-11.94084291	0.0015	0.0015
2	70	H	22.65039793	45.43686078	-10.46016154	-0.0033	-0.0032
2	71	H	16.34910591	42.36918565	-14.07793304	-0.0144	-0.0175
2	72	H	18.05263589	42.73770160	-14.40448460	-0.0270	-0.0339
2	73	H	17.22111151	43.52752481	-13.06432479	-0.0251	-0.0281
2	74	H	15.51436344	37.49105522	-12.22252983	0.0143	0.0138
2	75	H	13.52162806	40.88895516	-8.79572366	-0.0006	0.0002
2	76	H	13.91730865	39.27984954	-8.11232112	-0.0059	-0.0045
2	77	H	12.60337414	39.43338659	-9.30083089	-0.0023	-0.0020

Pigment No	Atom No	Atom Name	Coordinates			Vacuum TrESP Charge	Solvent TrESP Charge
2	78	H	12.25521386	34.00126698	-9.78695009	-0.0011	-0.0013
2	79	H	12.45757288	34.07343239	-11.56768031	-0.0008	-0.0018
2	80	H	12.78953302	35.52194304	-10.58036796	0.0019	0.0016
2	81	H	16.18598224	36.14969620	-9.59599637	0.0049	0.0025
2	82	H	18.45609717	34.65547460	-8.76664522	-0.0107	-0.0063
3	1	MG	29.81352548	35.20703989	6.07986765	-0.0058	0.0041
3	2	CHA	28.72672546	35.06551552	9.32335264	0.0909	-0.0794
3	3	CHB	27.56950688	37.65868953	5.43086244	-0.0414	0.0297
3	4	CHC	30.95414082	35.34653669	2.86700219	-0.1733	0.1803
3	5	CHD	32.08549955	32.65785134	6.76690511	0.0800	-0.0607
3	6	NA	28.40515851	36.24470298	7.23544276	0.0218	-0.0178
3	7	C1A	28.12274096	36.03579741	8.57264433	-0.1021	0.0909
3	8	C2A	27.09913333	37.03495146	9.07010239	-0.0505	0.0492
3	9	C3A	26.54322192	37.62166737	7.75873651	0.0158	-0.0062
3	10	C4A	27.56798854	37.17540710	6.72818574	0.0957	-0.0978
3	11	CMA	25.14168594	37.10422722	7.41714014	-0.0146	0.0187
3	12	CAA	27.75312441	38.10516957	9.96562266	0.0463	-0.0459
3	13	CBA	26.72895821	39.02503641	10.64943567	-0.0091	0.0078
3	14	CGA	25.98681891	38.28016677	11.73431673	0.0061	-0.0084
3	15	O1A	26.43933805	38.03109933	12.82493792	-0.0003	0.0009
3	16	O2A	24.76688686	37.88939831	11.32919540	-0.0035	0.0050
3	17	NB	29.32135288	36.27499078	4.41925923	-0.0697	0.0725
3	18	C1B	28.39625051	37.25837660	4.36595495	0.0472	-0.0469
3	19	C2B	28.36458263	37.84032982	3.03536719	0.0303	-0.0309
3	20	C3B	29.28579760	37.16601310	2.28673865	0.0118	-0.0132
3	21	C4B	29.91004312	36.18512782	3.17305543	0.1319	-0.1443
3	22	CMB	27.45067544	38.94568242	2.61403502	-0.0414	0.0441

Pigment No	Atom No	Atom Name	Coordinates			Vacuum TrESP Charge	Solvent TrESP Charge
3	23	CAB	29.58653668	37.40134169	0.87350333	-0.0306	0.0335
3	24	CBB	29.83572069	36.46706567	-0.04404028	0.0301	-0.0328
3	25	NC	31.24782919	34.17029950	5.01023858	-0.0119	0.0175
3	26	C1C	31.57801783	34.39689933	3.71570493	0.1542	-0.1638
3	27	C2C	32.64651121	33.52285354	3.30788999	-0.0330	0.0281
3	28	C3C	32.95724825	32.75071450	4.39956985	0.0518	-0.0477
3	29	C4C	32.07896510	33.16909579	5.46430743	-0.0917	0.0818
3	30	CMC	33.26185149	33.49065325	1.94341034	-0.0155	0.0190
3	31	CAC	33.96596695	31.64343055	4.48089777	-0.0213	0.0185
3	32	CBC	33.36111698	30.26770989	4.17299780	0.0140	-0.0125
3	33	ND	30.35059315	34.08875627	7.67295197	0.0661	-0.0645
3	34	C1D	31.27895953	33.06518759	7.82482555	-0.0769	0.0669
3	35	C2D	31.23439735	32.55437261	9.17402793	-0.0446	0.0531
3	36	C3D	30.24946082	33.29821479	9.80172195	-0.0247	0.0310
3	37	C4D	29.74781154	34.20893619	8.84530290	-0.0840	0.0813
3	38	CMD	32.06760971	31.45853226	9.75297834	0.0718	-0.0824
3	39	CAD	29.52730777	33.50542859	11.05395643	0.0024	-0.0077
3	40	OBD	29.65315788	32.96648980	12.13053736	-0.0235	0.0371
3	41	CBD	28.46635532	34.63255727	10.76934505	-0.0114	0.0109
3	42	CGD	27.05588589	34.10019853	10.92533757	0.0001	-0.0010
3	43	O1D	26.11965688	34.73155311	11.35930544	-0.0030	0.0048
3	44	O2D	26.94599248	32.84803324	10.46727434	0.0055	-0.0097
3	45	CED	25.64112510	32.27207840	10.54295098	-0.0017	0.0094
3	46	C1	24.03826183	37.09676388	12.27525233	-0.0002	-0.0010
3	47	H	26.84458265	38.43412907	5.20799840	0.0008	0.0011
3	48	H	31.35577169	35.42063428	1.86352567	0.0218	-0.0208
3	49	H	32.79275116	31.85982125	6.97230802	-0.0096	0.0057

Pigment No	Atom No	Atom Name	Coordinates			Vacuum TrESP Charge	Solvent TrESP Charge
3	50	H	24.79753543	37.49539441	6.45565075	-0.0016	-0.0017
3	51	H	24.43051857	37.40499749	8.19231690	0.0118	-0.0133
3	52	H	25.13602940	36.01085310	7.35722384	0.0074	-0.0085
3	53	H	28.43528054	38.71003086	9.35720663	-0.0130	0.0119
3	54	H	28.36000648	37.63148318	10.74288819	-0.0059	0.0068
3	55	H	26.01717853	39.42690786	9.92435161	-0.0022	0.0025
3	56	H	27.24941691	39.85962629	11.12588705	-0.0037	0.0048
3	57	H	27.62869712	39.24196756	1.57800714	0.0134	-0.0153
3	58	H	27.57835135	39.83285498	3.24403034	0.0185	-0.0207
3	59	H	26.39954323	38.64462202	2.69084614	0.0179	-0.0195
3	60	H	29.56883996	38.44319206	0.55771227	0.0082	-0.0093
3	61	H	29.82122686	35.40811437	0.19231662	-0.0016	0.0031
3	62	H	30.03881822	36.73635996	-1.07543019	0.0033	-0.0046
3	63	H	33.75733196	34.43671500	1.69747316	0.0023	-0.0044
3	64	H	32.51030220	33.30940902	1.16653074	0.0017	-0.0038
3	65	H	34.00879253	32.69784459	1.86151308	0.0059	-0.0066
3	66	H	34.78706176	31.84049334	3.78338927	0.0039	-0.0043
3	67	H	34.42240563	31.62555083	5.47680379	0.0093	-0.0076
3	68	H	32.95971496	30.23833135	3.15537111	-0.0044	0.0040
3	69	H	32.53860282	30.04091812	4.85788977	0.0002	-0.0003
3	70	H	34.11176413	29.47649400	4.26529307	0.0008	-0.0013
3	71	H	31.97285078	30.53365581	9.17375616	-0.0294	0.0336
3	72	H	31.75801931	31.25398060	10.77962833	-0.0182	0.0214
3	73	H	33.13019016	31.72628322	9.76538288	-0.0289	0.0364
3	74	H	28.58176122	35.43588415	11.50200745	0.0015	0.0003
3	75	H	25.75282317	31.24616163	10.19584840	0.0001	-0.0016
3	76	H	24.94487267	32.81891957	9.90286034	-0.0040	0.0021

Pigment No	Atom No	Atom Name	Coordinates			Vacuum TrESP Charge	Solvent TrESP Charge
3	77	H	25.27455633	32.29447825	11.57058902	-0.0010	0.0001
3	78	H	23.08497329	36.87601114	11.79603104	-0.0008	0.0006
3	79	H	23.88130714	37.65832255	13.19833323	-0.0009	0.0016
3	80	H	24.58591484	36.17889724	12.48744293	0.0015	-0.0011
3	81	H	26.31639799	36.53128694	9.64365161	0.0098	-0.0103
3	82	H	26.50968204	38.71615231	7.79670551	-0.0027	-0.0023
4	1	MG	21.85173606	30.00444529	8.46000170	-0.0072	-0.0079
4	2	CHA	25.03054334	28.67455743	8.40759250	-0.1225	-0.1207
4	3	CHB	21.72021982	29.77520846	5.08694655	0.0289	0.0191
4	4	CHC	18.70270540	31.27101550	8.55468944	0.1824	0.1863
4	5	CHD	22.09377492	30.32063775	11.91479948	-0.0940	-0.0723
4	6	NA	23.17825639	29.28113502	6.98911059	-0.0168	-0.0147
4	7	C1A	24.42930056	28.72979884	7.18144605	0.1011	0.0970
4	8	C2A	24.97641842	28.17237974	5.88481734	0.0988	0.0915
4	9	C3A	24.07739014	28.85336568	4.83419232	-0.0550	-0.0448
4	10	C4A	22.89716561	29.32964127	5.66731821	-0.0808	-0.0831
4	11	CMA	24.78068444	30.01232791	4.12210336	0.0189	0.0227
4	12	CAA	24.85776866	26.63566815	5.86798258	-0.0812	-0.0820
4	13	CBA	25.50924641	25.97502728	4.64291307	0.0407	0.0404
4	14	CGA	27.01107353	26.13081818	4.67859451	-0.0190	-0.0207
4	15	O1A	27.76172625	25.40671541	5.28460780	0.0018	0.0023
4	16	O2A	27.41879903	27.20564608	3.97968150	0.0088	0.0099
4	17	NB	20.42201715	30.38538063	7.06662855	0.0802	0.0838
4	18	C1B	20.56714535	30.25998890	5.73092644	-0.0517	-0.0535
4	19	C2B	19.35547609	30.69816222	5.05872865	-0.0132	-0.0150
4	20	C3B	18.47423785	31.06914503	6.03528700	-0.0187	-0.0185
4	21	C4B	19.16524647	30.90142011	7.31295611	-0.1416	-0.1528

Pigment No	Atom No	Atom Name	Coordinates			Vacuum TrESP Charge	Solvent TrESP Charge
4	22	CMB	19.14931608	30.69052178	3.57860085	0.0269	0.0304
4	23	CAB	17.10777868	31.55062042	5.82861842	0.0289	0.0307
4	24	CBB	16.05063175	31.26157310	6.58794470	-0.0269	-0.0298
4	25	NC	20.61837301	30.66730433	9.97207104	0.0275	0.0316
4	26	C1C	19.37049794	31.16881442	9.80258776	-0.1668	-0.1736
4	27	C2C	18.81769808	31.58000739	11.06601831	0.0363	0.0291
4	28	C3C	19.76992035	31.30464377	12.01707765	-0.0516	-0.0467
4	29	C4C	20.89642359	30.73663484	11.31932238	0.0902	0.0808
4	30	CMC	17.46662104	32.19641112	11.25578532	0.0158	0.0230
4	31	CAC	19.70529662	31.58576451	13.48898420	0.0209	0.0185
4	32	CBC	20.36428524	32.91668892	13.87367823	-0.0153	-0.0148
4	33	ND	23.24754483	29.65301315	9.88592184	-0.0588	-0.0571
4	34	C1D	23.21141964	29.80195061	11.26861195	0.0804	0.0666
4	35	C2D	24.44830716	29.33332339	11.84771060	0.0606	0.0760
4	36	C3D	25.20357243	28.90296491	10.77070219	-0.0031	-0.0023
4	37	C4D	24.42731224	29.11784544	9.60963374	0.0917	0.0928
4	38	CMD	24.82018384	29.31399386	13.29415845	-0.0803	-0.0973
4	39	CAD	26.46302065	28.28040026	10.37161362	-0.0024	-0.0099
4	40	OBD	27.39961303	27.90403687	11.03982978	0.0220	0.0355
4	41	CBD	26.40901256	28.14140822	8.80430215	0.0784	0.0927
4	42	CGD	27.52481169	28.92456942	8.14332112	-0.0254	-0.0317
4	43	O1D	28.14243053	28.56370392	7.16805267	0.0054	0.0077
4	44	O2D	27.71965701	30.10570388	8.74144172	0.0008	-0.0013
4	45	CED	28.73921984	30.92771076	8.17164249	-0.0050	-0.0007
4	46	C1	28.82171843	27.48845800	4.06897846	-0.0075	-0.0085
4	47	H	21.68774225	29.74458728	4.00283117	0.0021	0.0035
4	48	H	17.71418859	31.71411718	8.58423214	-0.0201	-0.0185

Pigment No	Atom No	Atom Name	Coordinates			Vacuum TrESP Charge	Solvent TrESP Charge
4	49	H	22.15254902	30.41205634	12.99540576	0.0093	0.0041
4	50	H	24.11601195	30.51052744	3.40997931	0.0033	0.0003
4	51	H	25.65341587	29.63793941	3.57841207	-0.0126	-0.0139
4	52	H	25.12198477	30.76112246	4.84404084	-0.0090	-0.0103
4	53	H	25.31723536	26.21749497	6.76906781	0.0147	0.0163
4	54	H	23.79672440	26.36182215	5.89980186	0.0150	0.0143
4	55	H	25.29580339	24.90366163	4.65640250	-0.0021	-0.0016
4	56	H	25.11395811	26.39647556	3.71475725	-0.0064	-0.0061
4	57	H	19.95386719	31.22265721	3.05960535	-0.0155	-0.0178
4	58	H	18.20445724	31.16543070	3.30444949	-0.0103	-0.0121
4	59	H	19.12804769	29.66964706	3.17996187	-0.0151	-0.0172
4	60	H	16.95621704	32.17643554	4.95063223	-0.0079	-0.0090
4	61	H	16.12210116	30.60281717	7.44713965	0.0002	0.0019
4	62	H	15.06804189	31.65801055	6.35387584	-0.0039	-0.0048
4	63	H	16.67358171	31.57651984	10.82319531	-0.0026	-0.0059
4	64	H	17.40131832	33.18208974	10.78144643	-0.0031	-0.0059
4	65	H	17.23380488	32.32906595	12.31526845	-0.0052	-0.0067
4	66	H	20.17928919	30.77071541	14.04770360	-0.0070	-0.0053
4	67	H	18.65925290	31.59856047	13.81217845	-0.0043	-0.0049
4	68	H	21.42634827	32.91915515	13.61110215	0.0017	0.0017
4	69	H	20.27705148	33.10140205	14.94911548	-0.0011	-0.0011
4	70	H	19.89171871	33.74991914	13.34501774	0.0046	0.0043
4	71	H	25.80361831	28.85773872	13.42112612	0.0205	0.0255
4	72	H	24.09758587	28.74437805	13.88848408	0.0318	0.0412
4	73	H	24.85697808	30.32656322	13.71134392	0.0309	0.0363
4	74	H	26.56613510	27.09222935	8.53837367	-0.0238	-0.0257
4	75	H	28.69063419	31.87358112	8.70869893	0.0016	0.0007

Pigment No	Atom No	Atom Name	Coordinates			Vacuum TrESP Charge	Solvent TrESP Charge
4	76	H	28.55800721	31.07724932	7.10546381	0.0060	0.0050
4	77	H	29.71804686	30.46234465	8.30483875	0.0027	0.0028
4	78	H	29.40389912	26.61213048	3.77834755	0.0032	0.0038
4	79	H	29.07247054	27.77581286	5.09074777	0.0013	0.0017
4	80	H	28.99860112	28.31260512	3.37847877	0.0018	0.0015
4	81	H	26.02545398	28.44558554	5.75007116	-0.0163	-0.0144
4	82	H	23.73421498	28.13614815	4.08155523	0.0129	0.0081

Table S2: Cartesian atomic coordinates and TrESP charges of the four Chl *a* chromophores in WSCP after geometry optimization of each individual pigment in vacuum with DFT using the CAM-B3LYP XC-functional and a 6-31-G* basis set, as described in the Computational Details section in the main text. The atom numbers are related to the structure of Chl *a* in Fig. S2. The attachments to this document include the above cartesian atomic coordinates in the file ***table_s2_coordinates.xyz*** as well as the two sets of TrESP charges in the file ***table_s2_charges.xyz***.

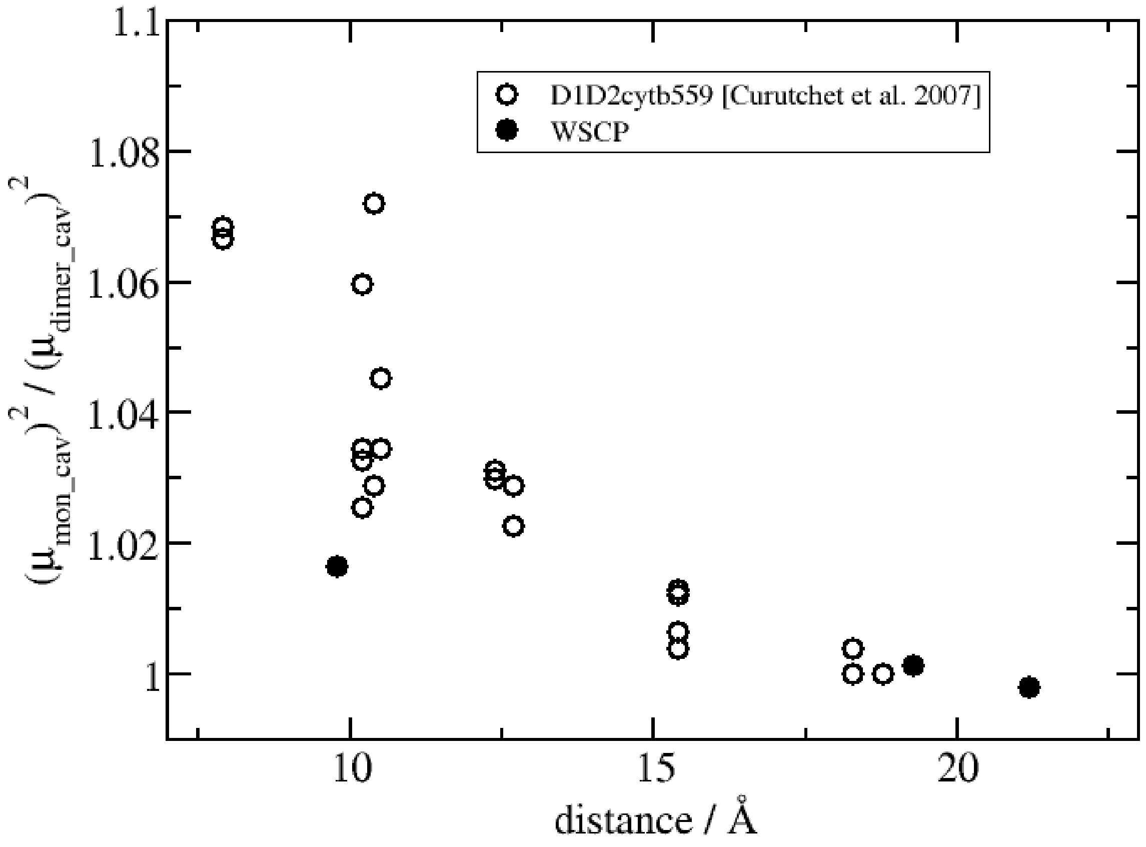

Figure S1: Ratio between dipole strengths obtained with TDDFT/PCM calculations for different Chl *a* pigments by taking into account a single pigment cavity or that of a dimer as a function of the inter-pigment distance in the dimer in D1D2cytb559 complexes (open circles, from the supporting information of ref [36]) of the main text and in WSCP (solid circles, present work).

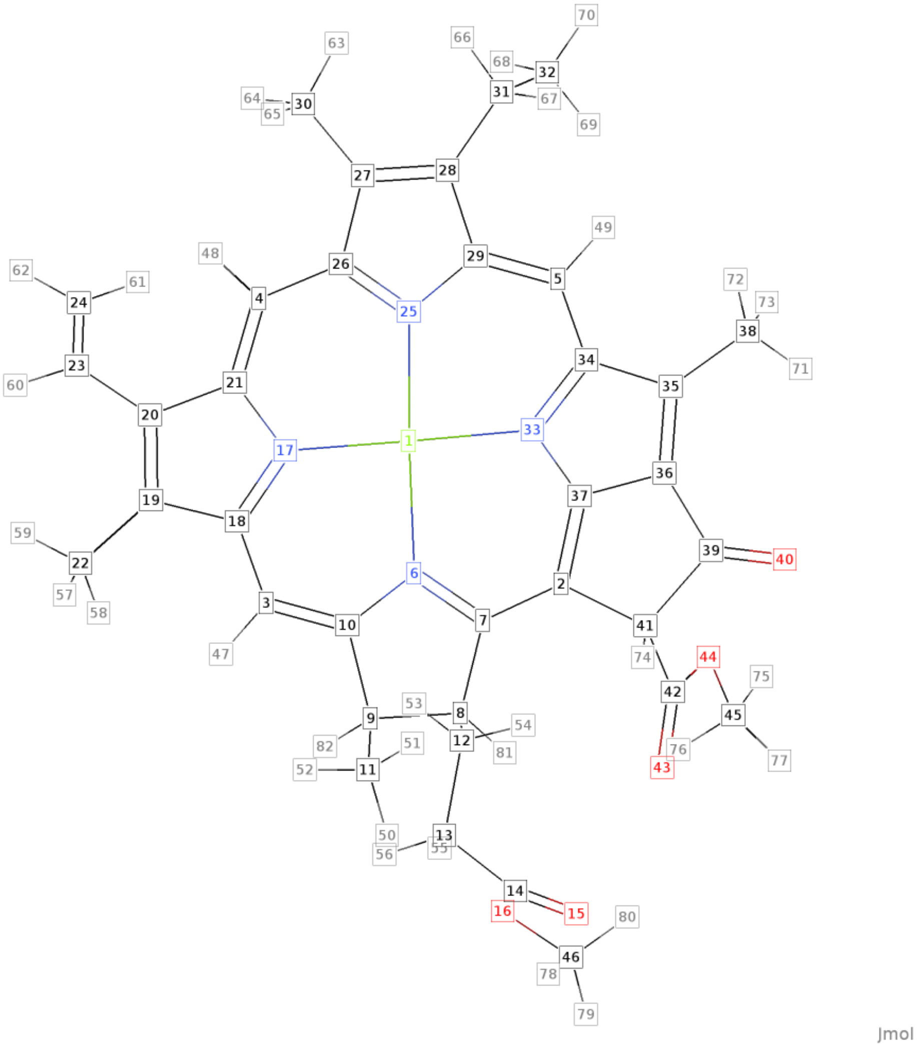

Figure S2: Numbering scheme used for the atomic coordinates of Chl *a* in Tables S2, S3 and S16.

Effect of geometry optimization on FMO1-FRET and FMO1-FRET-PCM1 calculations

Pigment No	Atom No	Atom Name	Coordinates		
			X (Å)	Y (Å)	Z (Å)
1	1	MG	12.32620626	40.67563648	-4.52304068
1	2	CHA	13.43664896	43.22824002	-6.54180337
1	3	CHB	14.71201195	41.48439021	-2.23255654
1	4	CHC	11.16362008	38.21073663	-2.50432382
1	5	CHD	9.93524733	39.87506929	-6.90308155
1	6	NA	13.82715323	42.16734484	-4.42050006
1	7	C1A	14.11805793	43.13838268	-5.35917175
1	8	C2A	15.25090686	44.03453816	-4.89467245
1	9	C3A	15.75312429	43.31624279	-3.62715289
1	10	C4A	14.69884609	42.24927484	-3.38898877
1	11	CMA	17.15547035	42.72045239	-3.78765642
1	12	CAA	14.78375853	45.47429877	-4.62072806
1	13	CBA	15.91789039	46.44159246	-4.24047396
1	14	CGA	16.86288390	46.69414583	-5.39240511
1	15	O1A	16.75535972	47.58873858	-6.19227926
1	16	O2A	17.84639183	45.77427068	-5.43285549
1	17	NB	12.87115822	39.96508859	-2.69739738
1	18	C1B	13.85721110	40.42341616	-1.89972076
1	19	C2B	13.90977727	39.64684522	-0.67260681
1	20	C3B	12.93415083	38.69651513	-0.76710769
1	21	C4B	12.25828771	38.90792165	-2.04733758
1	22	CMB	14.89080025	39.86032030	0.43400674
1	23	CAB	12.62907576	37.66990036	0.23171174
1	24	CBB	12.30285709	36.40050874	-0.01364315
1	25	NC	10.84980043	39.24645431	-4.69528377
1	26	C1C	10.48820021	38.35737300	-3.74258600
1	27	C2C	9.33924243	37.60121551	-4.16576890
1	28	C3C	9.00725861	38.06634499	-5.41437562

Pigment No	Atom No	Atom Name	Coordinates		
1	29	C4C	9.95670386	39.10488016	-5.73558852
1	30	CMC	8.65711708	36.53597992	-3.36449423
1	31	CAC	7.89976130	37.58920906	-6.30773618
1	32	CBC	8.33922258	36.47710441	-7.26716702
1	33	ND	11.78403841	41.33697265	-6.35213516
1	34	C1D	10.77758421	40.93601875	-7.22102912
1	35	C2D	10.76632468	41.78317875	-8.38918604
1	36	C3D	11.79208481	42.69114376	-8.17922347
1	37	C4D	12.37083387	42.37729188	-6.92875261
1	38	CMD	9.85136181	41.68809148	-9.56647314
1	39	CAD	12.51906543	43.82323847	-8.75063966
1	40	OBD	12.35318217	44.41249817	-9.79417048
1	41	CBD	13.63891056	44.19613322	-7.71028424
1	42	CGD	15.03247948	44.04933753	-8.28705754
1	43	O1D	15.95131275	44.80430690	-8.06382872
1	44	O2D	15.14789851	42.93629810	-9.01686311
1	45	CED	16.45235449	42.64508552	-9.52877877
1	46	C1	18.72364925	45.86741814	-6.56214868
1	47	H	15.48029002	41.73487033	-1.50887341
1	48	H	10.76389856	37.46027492	-1.83276102
1	49	H	9.16520081	39.62974366	-7.62804234
1	50	H	17.87631827	43.51788055	-3.98721821
1	51	H	17.18297444	42.01740252	-4.62665335
1	52	H	17.46720814	42.17993606	-2.88915287
1	53	H	14.04944540	45.46244065	-3.80692230
1	54	H	14.27192336	45.87602209	-5.49951063
1	55	H	15.48571261	47.40569209	-3.96382093
1	56	H	16.48512078	46.06016304	-3.38596746
1	57	H	14.79104860	40.85887489	0.87428337
1	58	H	15.92220934	39.76738135	0.07579896
1	59	H	14.75717921	39.12677726	1.23203500

Pigment No	Atom No	Atom Name	Coordinates		
1	60	H	12.71077212	37.98681217	1.27019879
1	61	H	12.24999566	35.99947468	-1.02066819
1	62	H	12.10142413	35.71046230	0.79924017
1	63	H	7.86567561	36.05104767	-3.94093471
1	64	H	8.19996266	36.94504713	-2.45627394
1	65	H	9.35670718	35.75404381	-3.04981195
1	66	H	7.06519052	37.23196557	-5.69504153
1	67	H	7.49921747	38.42907719	-6.88611373
1	68	H	8.69802825	35.60464730	-6.71252915
1	69	H	9.15576647	36.81420182	-7.91284735
1	70	H	7.50998969	36.15677415	-7.90584090
1	71	H	10.09080623	42.46776331	-10.29214160
1	72	H	8.80309552	41.80482305	-9.27060844
1	73	H	9.94264392	40.71847347	-10.06789578
1	74	H	13.52163094	45.24744483	-7.43668568
1	75	H	16.34341514	41.72165672	-10.09228894
1	76	H	17.15846948	42.51693734	-8.70484275
1	77	H	16.79877650	43.45677667	-10.17051385
1	78	H	19.51546667	45.14174952	-6.37775080
1	79	H	19.13506896	46.87503670	-6.63978057
1	80	H	18.17326591	45.62177412	-7.47127654
1	81	H	16.03412392	44.06448537	-5.65543184
1	82	H	15.76544427	44.00262201	-2.77242325
2	1	MG	20.33129563	39.26531711	-10.09069422
2	2	CHA	17.17049046	38.35258108	-11.07687788
2	3	CHB	20.51989122	36.46281849	-8.18007656
2	4	CHC	23.51113955	40.12753432	-9.21089713
2	5	CHD	20.08709007	42.11527668	-12.04822350
2	6	NA	19.02590979	37.67037485	-9.68173250
2	7	C1A	17.78481803	37.45799823	-10.24784584
2	8	C2A	17.25776637	36.08643150	-9.88938075

Pigment No	Atom No	Atom Name	Coordinates		
2	9	C3A	18.12549539	35.72328507	-8.66920987
2	10	C4A	19.32112325	36.64926193	-8.84518167
2	11	CMA	17.42667097	35.99837683	-7.33378231
2	12	CAA	17.45488799	35.11009667	-11.06938000
2	13	CBA	16.53644619	33.88851439	-10.98420800
2	14	CGA	15.12127522	34.28970309	-11.34105202
2	15	O1A	14.82325514	34.86131006	-12.36431201
2	16	O2A	14.23859247	33.95514925	-10.39435018
2	17	NB	21.76511495	38.45660351	-8.89190231
2	18	C1B	21.65599403	37.29442420	-8.21103292
2	19	C2B	22.88698546	37.02615249	-7.48824537
2	20	C3B	23.72626157	38.07565055	-7.73271142
2	21	C4B	23.02056661	38.97572735	-8.64332862
2	22	CMB	23.11804852	35.83551992	-6.61394141
2	23	CAB	25.06975552	38.24838315	-7.17802162
2	24	CBB	25.60263087	39.39245417	-6.74854367
2	25	NC	21.57405752	40.84621932	-10.54947548
2	26	C1C	22.83909373	41.01259092	-10.09255626
2	27	C2C	23.40694877	42.22364821	-10.62220327
2	28	C3C	22.44284784	42.79307235	-11.41806812
2	29	C4C	21.29587479	41.91995121	-11.36608628
2	30	CMC	24.78331305	42.73297926	-10.32586319
2	31	CAC	22.52621454	44.09354389	-12.16011897
2	32	CBC	22.05411385	45.29056448	-11.32548148
2	33	ND	18.92553445	40.13517839	-11.24399086
2	34	C1D	18.96771413	41.28686400	-12.02402373
2	35	C2D	17.73955555	41.41185939	-12.77841841
2	36	C3D	16.99056568	40.30136448	-12.42733031
2	37	C4D	17.75990666	39.56367890	-11.50147623
2	38	CMD	17.36799802	42.50791817	-13.72200438
2	39	CAD	15.73565695	39.57927015	-12.65065513

Pigment No	Atom No	Atom Name	Coordinates		
2	40	OBD	14.80903491	39.83891715	-13.38314505
2	41	CBD	15.78348661	38.30824367	-11.71935900
2	42	CGD	14.71834916	38.39333620	-10.64365530
2	43	O1D	13.99861557	37.48539391	-10.29578870
2	44	O2D	14.71216424	39.59810287	-10.06243313
2	45	CED	13.79175923	39.76038373	-8.97762853
2	46	C1	12.88950245	34.37315790	-10.64766211
2	47	H	20.59061203	35.58003781	-7.55422798
2	48	H	24.53147042	40.39055923	-8.95960155
2	49	H	20.02314362	42.99599949	-12.67973659
2	50	H	16.54502939	35.35998007	-7.21982158
2	51	H	17.09839486	37.04142509	-7.27805308
2	52	H	18.09913078	35.81168399	-6.49132054
2	53	H	17.24901042	35.61607566	-12.01601628
2	54	H	18.50408802	34.79524308	-11.10083309
2	55	H	16.56141263	33.42536482	-9.99468469
2	56	H	16.85434569	33.13933500	-11.71680581
2	57	H	22.87779807	34.90257938	-7.13436125
2	58	H	22.49242926	35.87388648	-5.71420692
2	59	H	24.15711034	35.77568667	-6.28253807
2	60	H	25.66183444	37.33916230	-7.08386407
2	61	H	25.05273492	40.32761069	-6.76329826
2	62	H	26.60801549	39.42077532	-6.34084129
2	63	H	25.03334109	43.58816722	-10.95792303
2	64	H	25.54483591	41.96447039	-10.49690273
2	65	H	24.88178075	43.05751794	-9.28324709
2	66	H	21.93559354	44.03617370	-13.08118052
2	67	H	23.55885520	44.26489877	-12.48254677
2	68	H	21.02489132	45.14717025	-10.98315578
2	69	H	22.09370149	46.21572102	-11.90901331
2	70	H	22.68238805	45.42027778	-10.43902683

Pigment No	Atom No	Atom Name	Coordinates		
2	71	H	16.42538211	42.27237320	-14.21953076
2	72	H	18.13465065	42.65948801	-14.48947310
2	73	H	17.24205176	43.46190562	-13.19700092
2	74	H	15.57849592	37.40647070	-12.30249972
2	75	H	13.92574786	40.78333205	-8.63652658
2	76	H	14.01742323	39.04368710	-8.18482284
2	77	H	12.76957390	39.59837098	-9.32198803
2	78	H	12.30347010	33.98258889	-9.81650196
2	79	H	12.53535153	33.96175365	-11.59500143
2	80	H	12.84741674	35.46213660	-10.68011239
2	81	H	16.19389698	36.13165849	-9.63419151
2	82	H	18.43955366	34.67418204	-8.69765313
3	1	MG	29.80984666	35.20398238	6.07802235
3	2	CHA	28.79274518	35.14014608	9.34690422
3	3	CHB	27.55036260	37.63795063	5.42073415
3	4	CHC	30.93976080	35.33035676	2.86096155
3	5	CHD	32.12804276	32.70117762	6.78460255
3	6	NA	28.40515851	36.24470298	7.23544276
3	7	C1A	28.15335996	36.07563941	8.58406926
3	8	C2A	27.12173653	37.07214284	9.07307360
3	9	C3A	26.54793733	37.62925381	7.75644569
3	10	C4A	27.56052102	37.16536994	6.72265318
3	11	CMA	25.14051134	37.10888949	7.44463131
3	12	CAA	27.75781388	38.16951871	9.94748331
3	13	CBA	26.72153369	39.10764259	10.58882496
3	14	CGA	25.95112466	38.39354462	11.67435319
3	15	O1A	26.35334395	38.21944240	12.79829661
3	16	O2A	24.76884297	37.93365321	11.22838777
3	17	NB	29.32135288	36.27499078	4.41925923
3	18	C1B	28.37912541	37.24151128	4.35773555
3	19	C2B	28.33046625	37.80520383	3.01941162

Pigment No	Atom No	Atom Name	Coordinates		
3	20	C3B	29.25527114	37.13331196	2.27377978
3	21	C4B	29.89816831	36.17132221	3.16862780
3	22	CMB	27.39635874	38.89006967	2.58909273
3	23	CAB	29.54160201	37.35293888	0.85485414
3	24	CBB	29.78757267	36.40924088	-0.05381211
3	25	NC	31.24782919	34.17029950	5.01023858
3	26	C1C	31.57124823	34.38834196	3.71332078
3	27	C2C	32.64444364	33.51797328	3.30824684
3	28	C3C	32.96907067	32.76138889	4.40633221
3	29	C4C	32.09318622	33.18342745	5.47239463
3	30	CMC	33.25322355	33.47915556	1.94111191
3	31	CAC	33.98908779	31.66552311	4.49669402
3	32	CBC	33.39460148	30.27854012	4.22161948
3	33	ND	30.35059315	34.08875627	7.67295197
3	34	C1D	31.32994903	33.11780309	7.84750886
3	35	C2D	31.36383822	32.69224893	9.22622808
3	36	C3D	30.37916689	33.44004654	9.85166075
3	37	C4D	29.80407641	34.27220704	8.86630003
3	38	CMD	32.26668751	31.67255428	9.83954689
3	39	CAD	29.69721271	33.69273097	11.11997257
3	40	OBD	29.88189417	33.21679689	12.21670965
3	41	CBD	28.59224445	34.77176567	10.81763407
3	42	CGD	27.20084705	34.20788536	11.02435947
3	43	O1D	26.27619365	34.80698100	11.52308203
3	44	O2D	27.09426499	32.97707021	10.51297417
3	45	CED	25.79848318	32.37213064	10.58529546
3	46	C1	24.02794281	37.14912109	12.17265554
3	47	H	26.81625469	38.40418191	5.19604982
3	48	H	31.33191994	35.39505386	1.85313829
3	49	H	32.86481207	31.93329801	7.00082077
3	50	H	24.78042035	37.49097265	6.48529772

Pigment No	Atom No	Atom Name	Coordinates		
3	51	H	24.44467486	37.41984070	8.22965767
3	52	H	25.13223977	36.01498103	7.39666811
3	53	H	28.44678228	38.76080629	9.33332882
3	54	H	28.35459025	37.72000104	10.74638660
3	55	H	26.02754760	39.49474899	9.83872292
3	56	H	27.23479047	39.95116139	11.05683346
3	57	H	27.56316620	39.17549795	1.54818547
3	58	H	27.51337481	39.78717966	3.20689916
3	59	H	26.35074015	38.57299880	2.67658622
3	60	H	29.51486978	38.39077086	0.52665088
3	61	H	29.78137758	35.35287654	0.19429280
3	62	H	29.97935586	36.66798245	-1.09006489
3	63	H	33.74886821	34.42362304	1.68941832
3	64	H	32.49774282	33.29623588	1.16856104
3	65	H	33.99850028	32.68491076	1.85890672
3	66	H	34.80067195	31.85774271	3.78686314
3	67	H	34.45606980	31.67095093	5.48778396
3	68	H	32.98527020	30.22533166	3.20810599
3	69	H	32.58008651	30.05701299	4.91788698
3	70	H	34.15338284	29.49667888	4.32389020
3	71	H	32.19986442	30.70849186	9.32404715
3	72	H	31.99990346	31.51990819	10.88719105
3	73	H	33.31440725	31.99094220	9.79941537
3	74	H	28.70644137	35.60800030	11.51163692
3	75	H	25.89512603	31.41881258	10.07185461
3	76	H	25.05735754	33.01080874	10.09991806
3	77	H	25.51027359	32.22221681	11.62709525
3	78	H	23.08037819	36.92425441	11.68399111
3	79	H	23.86247227	37.71829173	13.08905666
3	80	H	24.57545699	36.23363516	12.39778821
3	81	H	26.34916462	36.56606323	9.65851748

Pigment No	Atom No	Atom Name	Coordinates		
3	82	H	26.51530987	38.72417570	7.77239410
4	1	MG	21.85846833	30.01575309	8.45772755
4	2	CHA	24.97520587	28.56288638	8.41586815
4	3	CHB	21.73685211	29.82428554	5.08515411
4	4	CHC	18.69780728	31.26002440	8.55593411
4	5	CHD	22.07797256	30.28886405	11.91876130
4	6	NA	23.17825639	29.28113502	6.98911059
4	7	C1A	24.40237858	28.67715338	7.18123175
4	8	C2A	24.95424747	28.14858726	5.87354653
4	9	C3A	24.08416912	28.88189454	4.83346448
4	10	C4A	22.90686086	29.36097329	5.66657036
4	11	CMA	24.82114646	30.04130552	4.15688419
4	12	CAA	24.80904119	26.61715303	5.79211075
4	13	CBA	25.44584132	25.99674200	4.53762713
4	14	CGA	26.95101920	26.11999141	4.56181179
4	15	O1A	27.69672490	25.30863425	5.05208445
4	16	O2A	27.36980042	27.26806838	3.99726279
4	17	NB	20.42201715	30.38538063	7.06662855
4	18	C1B	20.57657267	30.28885394	5.72938701
4	19	C2B	19.36767485	30.73603152	5.05728705
4	20	C3B	18.47889509	31.08517216	6.03475637
4	21	C4B	19.16371586	30.89838987	7.31344249
4	22	CMB	19.17166795	30.75830890	3.57595284
4	23	CAB	17.11263237	31.56726226	5.82792628
4	24	CBB	16.05180233	31.26814245	6.57819034
4	25	NC	20.61837301	30.66730433	9.97207104
4	26	C1C	19.36590194	31.15685048	9.80362085
4	27	C2C	18.80431028	31.55139693	11.06882291
4	28	C3C	19.75437463	31.27463737	12.02102966
4	29	C4C	20.88921362	30.72302154	11.32211256
4	30	CMC	17.44558419	32.15120167	11.25756786

Pigment No	Atom No	Atom Name	Coordinates		
4	31	CAC	19.67690605	31.53502932	13.49604192
4	32	CBC	20.32574219	32.86349193	13.90535682
4	33	ND	23.24754483	29.65301315	9.88592184
4	34	C1D	23.19106090	29.75684822	11.27249178
4	35	C2D	24.38126028	29.18917529	11.85963771
4	36	C3D	25.12289043	28.72967627	10.78390357
4	37	C4D	24.38520201	29.02710701	9.61631278
4	38	CMD	24.71950341	29.10366447	13.31226748
4	39	CAD	26.35291294	28.04548193	10.38770495
4	40	OBD	27.25772538	27.60698789	11.06034512
4	41	CBD	26.31286653	27.94356909	8.81670806
4	42	CGD	27.46482360	28.69164686	8.18077249
4	43	O1D	28.14333780	28.27800396	7.26933591
4	44	O2D	27.60163374	29.91108303	8.71205717
4	45	CED	28.62391218	30.73243191	8.13941093
4	46	C1	28.78011121	27.50749536	4.08832487
4	47	H	21.71339914	29.81581397	4.00038475
4	48	H	17.70749731	31.69897136	8.58643326
4	49	H	22.12598758	30.35167608	13.00198927
4	50	H	24.17692324	30.56709393	3.44560722
4	51	H	25.69386031	29.65922916	3.61888292
4	52	H	25.16722240	30.76921310	4.89790544
4	53	H	25.26008893	26.15304386	6.67451164
4	54	H	23.74316403	26.36118259	5.81200184
4	55	H	25.21416696	24.93000781	4.50719605
4	56	H	25.05243827	26.46335190	3.63020087
4	57	H	19.97192283	31.31345708	3.07464211
4	58	H	18.22208194	31.22612658	3.30585393
4	59	H	19.16773748	29.74665277	3.15435036
4	60	H	16.96446653	32.20317661	4.95665891
4	61	H	16.11844558	30.59925702	7.42994820

Pigment No	Atom No	Atom Name	Coordinates		
4	62	H	15.07024509	31.66680845	6.34346019
4	63	H	16.66078468	31.52376732	10.82082918
4	64	H	17.37036306	33.13739101	10.78580239
4	65	H	17.20835694	32.27817039	12.31667078
4	66	H	20.15022995	30.71443904	14.04715905
4	67	H	18.62804177	31.53796522	13.80975118
4	68	H	21.39077153	32.87459077	13.65581957
4	69	H	20.22515540	33.03292187	14.98206087
4	70	H	19.85478026	33.70164750	13.38291999
4	71	H	25.70704516	28.65729046	13.44133376
4	72	H	23.99353854	28.48933609	13.85642184
4	73	H	24.72480345	30.09272377	13.78303945
4	74	H	26.41838739	26.89430411	8.52982210
4	75	H	28.56208788	31.68239962	8.66358218
4	76	H	28.45060079	30.85857028	7.06826212
4	77	H	29.60150925	30.27148147	8.28863822
4	78	H	29.33302868	26.69675199	3.61018870
4	79	H	29.07358059	27.58888898	5.13558473
4	80	H	28.95149703	28.44852793	3.56628363
4	81	H	26.00966456	28.40543637	5.76146722
4	82	H	23.73209451	28.19269723	4.05928962

Table S3: Cartesian atomic coordinates of the four Chl *a* chromophores in WSCP after geometry optimization on each dimer in vacuum with DFT using the CAM-B3LYP XC-functional and a 6-31-G* basis set, as described in the Computational Details section in the main text. During the geometry optimizations the coordinates of the nitrogen atoms were held fixed in order to preserve the relative orientation of the pigments. The atom numbers are related to the structure of Chl *a* in Fig. S2.The attachments to this document include the above cartesian atomic coordinates in the file ***table_s3_coordinates.xyz***.

N	Embedding	ω^N (eV)	μ^N (D)	ϑ^N (°)	φ^N (°)
1	none	2.147	5.26	-1.4	-6.8
	impl	2.111	6.33	-1.5	-6.2
	impl+expl		3.65	-3.0	-4.5
2	none	2.140	5.26	-2.7	-6.8
	impl	2.103	6.33	-2.7	-6.0
	impl+expl		3.60	-4.1	-4.9
3	none	2.148	5.30	-1.8	-6.2
	impl	2.112	6.36	-1.9	-5.7
	impl+expl		3.66	-3.4	-4.5
4	none	2.143	5.26	-3.0	-6.4
	impl	2.105	6.34	-3.3	-5.8
	impl+expl		3.63	-5.0	-4.4

Table S4: Same as in Table 3 of the main text, but using the optimized dimer geometry (Table S3).

M	N	$V_{MN}^{ES}(\varepsilon=1)$	$V_{MN}^{ES}(\varepsilon=2)$	$V_{MN}^{ES}(\varepsilon=2)+V_{MN}^{solv}(\varepsilon=2)$	f_{MN}^{rf}	s_{MN}
3	4	132	188	116	1.422	0.618
1	2	131	188	116	1.431	0.620
2	3	34	47	29	1.383	0.600
1	4	32	44	27	1.397	0.602
2	4	10	15	10	1.512	0.680
1	3	9	14	10	1.496	0.703

Table S5: Same as in Table 4 of the main text but using the optimized dimer geometry (Table S3).

Effect of varying the static dielectric constant on FMO0-FRET and FMO0-FRET-PCM0 calculations

N	**Embedding**	$\boldsymbol{\omega^N}$ **(eV)**	$\boldsymbol{\mu^N}$ **(D)**	$\boldsymbol{\vartheta^N}$ **(°)**	$\boldsymbol{\varphi^N}$ **(°)**
1	none	2.140	5.31	0.0	-6.9
	impl	2.100	6.39	-0.1	-5.9
	impl+expl	2.100	3.68	-0.8	-4.4
2	none	2.131	5.32	-1.8	-6.6
	impl	2.090	6.36	-1.8	-5.8
	impl+expl	2.090	3.63	-2.4	-4.5
3	none	2.136	5.36	0.1	-6.4
	impl	2.096	6.43	0.0	-5.7
	impl+expl	2.096	3.71	-1.1	-4.5
4	none	2.134	5.32	-1.4	-6.2
	impl	2.094	6.40	-1.6	-5.6
	impl+expl	2.094	3.66	-3.6	-4.3

Table S6: Same as in Table 1 of the main text, but using $\varepsilon_s = 2$.

M	***N***	$V_{MN}^{ES}(\varepsilon=1)$	$V_{MN}^{ES}(\varepsilon=2)$	$V_{MN}^{ES}(\varepsilon=2)+V_{MN}^{solv}(\varepsilon=2)$	f_{MN}^{rf}	s_{MN}
3	4	147	208	129	1.412	0.621
1	2	142	201	125	1.409	0.621
2	3	35	48	29	1.394	0.599
1	4	32	45	27	1.416	0.602
2	4	10	14	10	1.462	0.684
1	3	9	12	9	1.452	0.716

Table S7: Same as in Table 2 of the main text, but using $\varepsilon_s = 2$.

N	Embedding	ω^N (eV)	μ^N (D)	ϑ^N (°)	φ^N (°)
1	none	2.140	5.31	0.0	-6.9
	impl	2.102	6.42	-0.1	-5.3
	impl+expl	2.102	3.71	-0.7	-3.8
2	none	2.131	5.32	-1.8	-6.6
	impl	2.092	6.42	-1.7	-5.4
	impl+expl	2.092	3.66	-2.4	-4.1
3	none	2.136	5.36	0.1	-6.4
	impl	2.098	6.45	0.0	-5.1
	impl+expl	2.098	3.73	-1.1	-4.0
4	none	2.134	5.32	-1.4	-6.2
	impl	2.095	6.43	-1.7	-5.0
	impl+expl	2.095	3.69	-3.6	-3.6

Table S8: Same as in Table 1 of the main text, but using $\varepsilon_s = 10$.

M	N	$V_{MN}^{ES}(\varepsilon=1)$	$V_{MN}^{ES}(\varepsilon=2)$	$V_{MN}^{ES}(\varepsilon=2)+V_{MN}^{solv}(\varepsilon=2)$	f_{MN}^{rf}	s_{MN}
3	4	147	212	132	1.611	0.621
1	2	142	206	128	1.610	0.621
2	3	35	48	29	1.666	0.600
1	4	32	44	27	1.658	0.603
2	4	10	15	10	1.468	0.681
1	3	9	13	9	1.407	0.711

Table S9: Same as in Table 2 of the main text, but using $\varepsilon_s = 10$.

N	Embedding	ω^N (eV)	μ^N (D)	ϑ^N (°)	φ^N (°)
1	none	2.140	5.31	0.0	-6.9
	impl	2.102	6.42	-0.1	-5.2
	impl+expl	2.102	3.71	-0.7	-3.6
2	none	2.131	5.32	-1.8	-6.6
	impl	2.092	6.42	-1.7	-5.3
	impl+expl	2.092	3.66	-2.4	-4.0
3	none	2.136	5.36	0.1	-6.4
	impl	2.098	6.46	0.0	-5.0
	impl+expl	2.098	3.74	-1.1	-3.8
4	none	2.134	5.32	-1.4	-6.2
	impl	2.096	6.45	-1.7	-4.9
	impl+expl	2.096	3.70	-3.6	-3.6

Table S10: Same as in Table 1 of the main text, but using $\varepsilon_s = 20$.

M	N	$V_{MN}^{ES}(\varepsilon=1)$	$V_{MN}^{ES}(\varepsilon=2)$	$V_{MN}^{ES}(\varepsilon=2)+V_{MN}^{solv}(\varepsilon=2)$	f_{MN}^{rf}	s_{MN}
3	4	147	213	132	1.610	0.621
1	2	142	207	128	1.610	0.621
2	3	35	47	28	1.665	0.601
1	4	32	44	27	1.657	0.603
2	4	10	15	10	1.470	0.680
1	3	9	13	9	1.409	0.710

Table S11: Same as in Table 2 of the main text, but using $\varepsilon_s = 20$.

Effect of varying the functional on FMO0-FRET and FMO0-FRET-PCM0 calculations

N	Embedding	ω^N (eV)	μ^N (D)	ϑ^N (°)	φ^N (°)
1	none	2.431	6.21	0.2	-8.7
	impl	2.391	7.10	0.2	-8.8
	impl+expl	2.391	4.10	-0.4	-7.0
2	none	2.410	6.22	-1.4	-7.9
	impl	2.370	7.10	-1.4	-8.0
	impl+expl	2.370	4.06	-2.1	-6.6
3	none	2.423	6.25	0.2	-7.9
	impl	2.383	7.15	0.2	-8.1
	impl+expl	2.383	4.14	-1.2	-6.9
4	none	2.419	6.22	-1.5	-7.9
	impl	2.377	7.13	-1.8	-8.0
	impl+expl	2.377	4.07	-3.8	-6.7

Table S12: Same as in Table 1 of the main text, but using HF-CIS.

M	N	$V_{MN}^{ES}(\varepsilon=1)$	$V_{MN}^{ES}(\varepsilon=2)$	$V_{MN}^{ES}(\varepsilon=2)+V_{MN}^{solv}(\varepsilon=2)$	f_{MN}^{rf}	s_{MN}
3	4	205	265	164	1.293	0.618
1	2	198	256	158	1.292	0.619
2	3	44	55	33	1.255	0.601
1	4	41	52	31	1.272	0.603
2	4	12	16	11	1.296	0.701
1	3	10	13	10	1.292	0.737

Table S13: Same as in Table 2 of the main text, but using HF-CIS.

N	Embedding	ω^N (eV)	μ^N (D)	ϑ^N (°)	φ^N (°)
1	none	2.159	5.25	0.0	-6.6
	impl	2.115	6.45	0.0	-6.0
	impl+expl	2.115	3.72	-0.9	-4.4
2	none	2.154	5.23	-1.7	-6.9
	impl	2.110	6.42	-1.7	-6.2
	impl+expl	2.110	3.67	-2.2	-5.0
3	none	2.156	5.28	0.1	-6.3
	impl	2.113	6.48	0.1	-5.9
	impl+expl	2.113	3.75	-0.7	-4.8
4	none	2.156	5.24	-1.0	-6.3
	impl	2.112	6.46	-1.3	-5.8
	impl+expl	2.112	3.70	-3.3	-4.6

Table S14: Same as in Table 1 of the main text, but using the B3LYP XC-functional.

M	N	$V_{MN}^{ES}(\varepsilon=1)$	$V_{MN}^{ES}(\varepsilon=2)$	$V_{MN}^{ES}(\varepsilon=2)+V_{MN}^{solv}(\varepsilon=2)$	f_{MN}^{rf}	s_{MN}
3	4	138	206	128	1.492	0.621
1	2	134	200	124	1.492	0.621
2	3	36	51	31	1.440	0.597
1	4	33	48	29	1.458	0.600
2	4	8	13	9	1.583	0.691
1	3	7	12	8	1.570	0.723

Table S15: Same as in Table 2 of the main text, but using the B3LYP XC-functional.

Geometry optimization with Hartree-Fock to eliminate grid dependence

Pigment No	Atom No	Atom Name	Coordinates		
			X (Å)	Y (Å)	Z (Å)
1	1	MG	12.39356320	40.61689038	-4.55971318
1	2	CHA	13.68516146	43.01130108	-6.64824268
1	3	CHB	14.81343882	41.34775597	-2.29748413
1	4	CHC	11.12021273	38.27799663	-2.46038364
1	5	CHD	9.95435005	39.86534116	-6.88748466
1	6	NA	13.97673627	42.03719796	-4.47437405
1	7	C1A	14.30150510	42.97834223	-5.45985957
1	8	C2A	15.37312138	43.91661887	-4.93475700
1	9	C3A	15.94486891	43.10696769	-3.75417445
1	10	C4A	14.83519244	42.10283536	-3.48282167
1	11	CMA	17.26223308	42.39734661	-4.09549894
1	12	CAA	14.74889012	45.25385485	-4.48544011
1	13	CBA	15.77738674	46.33146657	-4.09967419
1	14	CGA	16.47782484	46.88208692	-5.32063793
1	15	O1A	15.99701852	47.69261304	-6.04520182
1	16	O2A	17.67392711	46.34793533	-5.50693648
1	17	NB	12.89280075	39.92286737	-2.72796570
1	18	C1B	13.90316461	40.35686080	-1.96710272
1	19	C2B	13.92370709	39.60704679	-0.69473120
1	20	C3B	12.91561547	38.72597363	-0.74601803
1	21	C4B	12.22682453	38.92813289	-2.04195577
1	22	CMB	14.93089549	39.82011770	0.39490706
1	23	CAB	12.55186410	37.74525627	0.29643389
1	24	CBB	12.30711682	36.46283032	0.09131602
1	25	NC	10.79810915	39.29324834	-4.66113689
1	26	C1C	10.43420682	38.44806981	-3.72252065
1	27	C2C	9.26489214	37.69823743	-4.12081827
1	28	C3C	8.93808167	38.13970601	-5.36544984

Pigment No	Atom No	Atom Name	Coordinates		
1	29	C4C	9.90764820	39.15222878	-5.71709889
1	30	CMC	8.59075342	36.64561495	-3.28592846
1	31	CAC	7.81506141	37.65952682	-6.24634901
1	32	CBC	8.23547909	36.52132527	-7.18603741
1	33	ND	11.91576321	41.22677960	-6.40638580
1	34	C1D	10.88608405	40.86030961	-7.25834875
1	35	C2D	10.93009441	41.62315875	-8.44165913
1	36	C3D	12.03175097	42.47146250	-8.27376964
1	37	C4D	12.57203076	42.17583877	-7.02686706
1	38	CMD	10.01812006	41.54416169	-9.63125384
1	39	CAD	12.78814532	43.55622253	-8.87756602
1	40	OBD	12.65082947	44.12292895	-9.91940464
1	41	CBD	13.91675989	43.92756900	-7.85739771
1	42	CGD	15.28937095	43.71985347	-8.46456101
1	43	O1D	16.20680007	44.46624869	-8.32297854
1	44	O2D	15.37165269	42.58833774	-9.13846908
1	45	CED	16.61082687	42.28575232	-9.75282631
1	46	C1	18.37367733	46.74087437	-6.68047518
1	47	H	15.58392210	41.56620184	-1.58348800
1	48	H	10.69723174	37.55661595	-1.79102758
1	49	H	9.18856773	39.64739452	-7.60940139
1	50	H	18.02955168	43.13069217	-4.31862277
1	51	H	17.14410766	41.75786793	-4.96477062
1	52	H	17.60615626	41.78257461	-3.27131344
1	53	H	14.09904930	45.07137714	-3.63402476
1	54	H	14.12053839	45.65175374	-5.27285123
1	55	H	15.25765285	47.16111365	-3.63630378
1	56	H	16.50805114	45.94957283	-3.39790293
1	57	H	14.88307193	40.83436696	0.77991699
1	58	H	15.93979348	39.65763427	0.02791638
1	59	H	14.76696709	39.13811461	1.21894350

Pigment No	Atom No	Atom Name	Coordinates		
1	60	H	12.51825506	38.12434193	1.30425475
1	61	H	12.35306367	36.01929882	-0.88678064
1	62	H	12.06695909	35.80786780	0.90926895
1	63	H	7.72582214	36.23656951	-3.79255655
1	64	H	8.25208236	37.04540841	-2.33418334
1	65	H	9.26032464	35.81729842	-3.07012956
1	66	H	6.99008446	37.32572686	-5.62692930
1	67	H	7.42886364	38.48679641	-6.83254557
1	68	H	8.57856018	35.66016210	-6.62244267
1	69	H	9.04410722	36.83153194	-7.83912052
1	70	H	7.40096281	36.20850908	-7.80607939
1	71	H	10.10733326	42.44211566	-10.22867021
1	72	H	8.97973668	41.43127102	-9.33778504
1	73	H	10.27689951	40.70028019	-10.26519843
1	74	H	13.83779552	44.98049496	-7.61901770
1	75	H	16.46352123	41.34734659	-10.26331096
1	76	H	17.38881533	42.19047920	-9.00868469
1	77	H	16.88050892	43.05850450	-10.45755203
1	78	H	19.31066862	46.20654455	-6.65296240
1	79	H	18.55009689	47.80666792	-6.67082712
1	80	H	17.80857877	46.46809006	-7.55721392
1	81	H	16.12654746	44.10662133	-5.68666214
1	82	H	16.10139686	43.72534024	-2.87736179
2	1	MG	20.30756365	39.31637283	-10.13175483
2	2	CHA	17.11708965	38.49813512	-11.08797291
2	3	CHB	20.43817567	36.47212421	-8.28892580
2	4	CHC	23.47365982	40.08596935	-9.19196140
2	5	CHD	20.14725535	42.22226183	-11.99377167
2	6	NA	18.97699173	37.68786372	-9.80588565
2	7	C1A	17.69635777	37.53968884	-10.35379699
2	8	C2A	17.15638165	36.16067900	-10.02072364

Pigment No	Atom No	Atom Name	Coordinates		
2	9	C3A	18.05534046	35.75167879	-8.83715857
2	10	C4A	19.24081452	36.69203739	-8.99141123
2	11	CMA	17.37493889	35.93800468	-7.47415124
2	12	CAA	17.30646469	35.21428079	-11.22968528
2	13	CBA	16.62912944	33.84502374	-11.04519100
2	14	CGA	15.12418524	33.96563796	-11.11745655
2	15	O1A	14.52068327	34.06420294	-12.13687700
2	16	O2A	14.55468231	33.97224401	-9.92321813
2	17	NB	21.68983840	38.45146225	-8.93735344
2	18	C1B	21.55631690	37.29048931	-8.28750920
2	19	C2B	22.79390477	36.99706806	-7.53663278
2	20	C3B	23.63399635	38.01727392	-7.75727795
2	21	C4B	22.94696416	38.95575539	-8.67478157
2	22	CMB	22.99516506	35.78228206	-6.68167347
2	23	CAB	24.99237496	38.17333996	-7.19939813
2	24	CBB	25.46601594	39.26684548	-6.62798158
2	25	NC	21.59452140	40.89552685	-10.53066713
2	26	C1C	22.82333370	41.02316568	-10.08156194
2	27	C2C	23.43852120	42.23113806	-10.58176446
2	28	C3C	22.50479234	42.83460357	-11.36595601
2	29	C4C	21.32982151	41.99361187	-11.33805420
2	30	CMC	24.83532204	42.68388458	-10.26006688
2	31	CAC	22.62722364	44.14384437	-12.09984414
2	32	CBC	22.14639251	45.34239716	-11.27067565
2	33	ND	18.93547164	40.24084421	-11.26055219
2	34	C1D	18.98522365	41.41922577	-11.98812837
2	35	C2D	17.77469519	41.62372035	-12.67886462
2	36	C3D	16.98418492	40.51841446	-12.34024508
2	37	C4D	17.74981699	39.73145438	-11.48663051
2	38	CMD	17.39613889	42.77231227	-13.56798222
2	39	CAD	15.70525378	39.86722473	-12.57236469

Pigment No	Atom No	Atom Name	Coordinates		
2	40	OBD	14.77403645	40.18650576	-13.24787309
2	41	CBD	15.72738343	38.54246400	-11.73758681
2	42	CGD	14.62296390	38.53376068	-10.70019713
2	43	O1D	13.95237149	37.58416376	-10.44052296
2	44	O2D	14.51264405	39.69215192	-10.07796221
2	45	CED	13.51158765	39.80334718	-9.08294465
2	46	C1	13.14733109	34.17063133	-9.88741517
2	47	H	20.48086192	35.57960127	-7.69501180
2	48	H	24.48250016	40.32165194	-8.91984837
2	49	H	20.09663135	43.11630303	-12.58807547
2	50	H	16.51170965	35.28553025	-7.40010897
2	51	H	17.03581318	36.96138544	-7.34684913
2	52	H	18.05069269	35.70484742	-6.65898768
2	53	H	16.89397115	35.67909434	-12.11676565
2	54	H	18.36349486	35.05733084	-11.42600249
2	55	H	16.91652068	33.38274838	-10.10932654
2	56	H	16.93026029	33.19347731	-11.85638354
2	57	H	22.92169722	34.87242360	-7.26991861
2	58	H	22.24221516	35.72797711	-5.90127202
2	59	H	23.96514602	35.79693534	-6.20213198
2	60	H	25.62126839	37.30000922	-7.24755885
2	61	H	24.87798289	40.16068181	-6.52418397
2	62	H	26.46318736	39.29391521	-6.22721331
2	63	H	25.07700211	43.60661635	-10.77218447
2	64	H	25.57408659	41.94416061	-10.55556414
2	65	H	24.96261332	42.86226387	-9.19561285
2	66	H	22.06607425	44.09892345	-13.02731224
2	67	H	23.66112134	44.29940689	-12.38724705
2	68	H	21.10523725	45.22996458	-10.98783149
2	69	H	22.24569542	46.26389262	-11.83610598
2	70	H	22.72690782	45.44128662	-10.35954745

Pigment No	Atom No	Atom Name	Coordinates		
2	71	H	16.54725959	42.50267660	-14.18271110
2	72	H	18.21179307	43.06354898	-14.22144701
2	73	H	17.11429805	43.64268873	-12.98149040
2	74	H	15.53740079	37.70899304	-12.40163381
2	75	H	13.57092004	40.81686549	-8.71941118
2	76	H	13.69736191	39.10464023	-8.27958461
2	77	H	12.53655809	39.61264157	-9.50657213
2	78	H	12.87667521	34.14608085	-8.84321151
2	79	H	12.64474323	33.37847037	-10.42315908
2	80	H	12.89701938	35.12573458	-10.32053121
2	81	H	16.11609339	36.20985948	-9.72994487
2	82	H	18.39023150	34.72372016	-8.91983760
3	1	MG	29.81297856	35.16768082	6.07301951
3	2	CHA	28.61034904	34.96560629	9.27868294
3	3	CHB	27.51941285	37.57766250	5.41136004
3	4	CHC	30.99911927	35.40940034	2.90545637
3	5	CHD	32.12370650	32.69219344	6.75649599
3	6	NA	28.32769478	36.16972804	7.22205143
3	7	C1A	28.03211087	35.94574659	8.57293345
3	8	C2A	27.04056970	36.98827734	9.05704410
3	9	C3A	26.45657230	37.50408399	7.72699734
3	10	C4A	27.50776408	37.06401195	6.71970695
3	11	CMA	25.08596718	36.89693099	7.39783574
3	12	CAA	27.76359485	38.09485264	9.85229665
3	13	CBA	26.81947608	39.10388092	10.52929417
3	14	CGA	26.11480363	38.48429926	11.71422532
3	15	O1A	26.62201541	38.34058928	12.77967697
3	16	O2A	24.88104232	38.09969701	11.43029078
3	17	NB	29.32845452	36.27085075	4.44997489
3	18	C1B	28.37391244	37.20515425	4.38634468
3	19	C2B	28.34600349	37.79445612	3.03225365

Pigment No	Atom No	Atom Name	Coordinates		
3	20	C3B	29.29260316	37.17163415	2.31724962
3	21	C4B	29.95071567	36.19922976	3.22064589
3	22	CMB	27.39098711	38.86462417	2.59657845
3	23	CAB	29.62560358	37.41536176	0.89934267
3	24	CBB	29.78150859	36.48231055	-0.02354132
3	25	NC	31.31505610	34.20682140	5.01074127
3	26	C1C	31.65431737	34.44674499	3.76371909
3	27	C2C	32.76107690	33.61251233	3.35465704
3	28	C3C	33.07610495	32.84593909	4.43349341
3	29	C4C	32.16159812	33.21595465	5.48976724
3	30	CMC	33.39395365	33.62795177	1.99130222
3	31	CAC	34.13851261	31.78286257	4.52767245
3	32	CBC	33.61912173	30.38369992	4.17012951
3	33	ND	30.26962534	34.00322831	7.63720697
3	34	C1D	31.24582572	33.03474960	7.80903246
3	35	C2D	31.21268399	32.52892821	9.12318267
3	36	C3D	30.17303544	33.23147433	9.74554726
3	37	C4D	29.65534768	34.09955314	8.79039996
3	38	CMD	32.07764666	31.46272830	9.72970132
3	39	CAD	29.46810607	33.42729679	11.00176386
3	40	OBD	29.60740005	32.90883567	12.06817606
3	41	CBD	28.39856689	34.54038696	10.73797172
3	42	CGD	26.99677366	34.01466103	10.97139875
3	43	O1D	26.13307956	34.62437370	11.52015587
3	44	O2D	26.82184213	32.81229854	10.45664615
3	45	CED	25.54690497	32.21685811	10.61323748
3	46	C1	24.16894437	37.42537579	12.45960461
3	47	H	26.78827961	38.33191778	5.19297250
3	48	H	31.39586975	35.49951335	1.91460169
3	49	H	32.85098266	31.93331416	6.98047623
3	50	H	24.72934973	37.22616546	6.42833913

Pigment No	Atom No	Atom Name	Coordinates		
3	51	H	24.36099460	37.19397689	8.14786369
3	52	H	25.13479536	35.81248626	7.38718864
3	53	H	28.42816937	38.63384704	9.18272765
3	54	H	28.38988590	37.65164769	10.61682807
3	55	H	26.09016799	39.49508323	9.83125584
3	56	H	27.40643355	39.93022401	10.91113059
3	57	H	27.53925638	39.12239868	1.55609892
3	58	H	27.51502019	39.76567079	3.18980409
3	59	H	26.36221336	38.53703941	2.71191203
3	60	H	29.71488981	38.44941653	0.61074375
3	61	H	29.67688963	35.43520729	0.19582543
3	62	H	30.00508210	36.74373905	-1.04205323
3	63	H	33.78422166	34.61074333	1.74203039
3	64	H	32.68179505	33.35562212	1.21689867
3	65	H	34.21708420	32.92717939	1.93229858
3	66	H	34.96148748	32.03799703	3.86937727
3	67	H	34.55494682	31.76754215	5.52939130
3	68	H	33.24302875	30.35804621	3.15286800
3	69	H	32.80990933	30.08670228	4.82862134
3	70	H	34.41229705	29.64729459	4.25563198
3	71	H	31.72948333	30.47249964	9.44820818
3	72	H	32.04468565	31.52625096	10.80959468
3	73	H	33.11165857	31.55212910	9.41385928
3	74	H	28.55138015	35.34498501	11.44576068
3	75	H	25.61842070	31.24517664	10.15104646
3	76	H	24.78893658	32.81115737	10.12294898
3	77	H	25.30362821	32.11759027	11.66084117
3	78	H	23.20095775	37.19636296	12.04154802
3	79	H	24.06194301	38.06729042	13.32194028
3	80	H	24.68553105	36.52002191	12.73514612
3	81	H	26.27711609	36.54020724	9.67792593

Pigment No	Atom No	Atom Name	Coordinates		
3	82	H	26.36848483	38.58473140	7.71559565
4	1	MG	21.84908084	30.01240908	8.47564178
4	2	CHA	25.02828298	28.72831264	8.36403023
4	3	CHB	21.70548427	29.83387097	5.09262792
4	4	CHC	18.70527512	31.28113979	8.55467431
4	5	CHD	22.06705668	30.32145224	11.90986015
4	6	NA	23.16631226	29.30158050	6.96285528
4	7	C1A	24.44350328	28.76075022	7.15975565
4	8	C2A	24.97349009	28.21643133	5.84558694
4	9	C3A	24.08028172	28.94768511	4.82388941
4	10	C4A	22.90001363	29.38205673	5.67934281
4	11	CMA	24.77263677	30.15643677	4.17966675
4	12	CAA	24.80495065	26.68416263	5.78803214
4	13	CBA	25.46459059	26.02200433	4.56551523
4	14	CGA	26.97059984	26.01204816	4.69338707
4	15	O1A	27.56958868	25.22644091	5.35454160
4	16	O2A	27.54719599	26.98550943	4.00728418
4	17	NB	20.44576205	30.43093219	7.08279395
4	18	C1B	20.58414996	30.30126525	5.75917634
4	19	C2B	19.34413281	30.73714734	5.08559979
4	20	C3B	18.48644850	31.10276038	6.04804557
4	21	C4B	19.18975887	30.93992240	7.34174098
4	22	CMB	19.13728559	30.71433716	3.60102575
4	23	CAB	17.10588185	31.58971454	5.85616394
4	24	CBB	16.04544330	31.17159349	6.52507971
4	25	NC	20.59452865	30.68357361	9.98737495
4	26	C1C	19.37982635	31.16039171	9.82866133
4	27	C2C	18.80329396	31.55064303	11.09486826
4	28	C3C	19.74420912	31.27826217	12.03908310
4	29	C4C	20.88652960	30.73022710	11.34392522
4	30	CMC	17.43199627	32.14029127	11.27207831

Pigment No	Atom No	Atom Name	Coordinates		
4	31	CAC	19.65881068	31.52751450	13.52173292
4	32	CBC	20.20769394	32.90097016	13.93109177
4	33	ND	23.23456771	29.65856998	9.87778688
4	34	C1D	23.20475403	29.79950538	11.25590087
4	35	C2D	24.41438707	29.35320615	11.82359311
4	36	C3D	25.18328686	28.93472047	10.73097196
4	37	C4D	24.40835901	29.14980276	9.59608531
4	38	CMD	24.79695016	29.31256732	13.27489193
4	39	CAD	26.45753946	28.35640869	10.33682950
4	40	OBD	27.39834040	28.01443410	10.98750755
4	41	CBD	26.41777000	28.22392859	8.77672508
4	42	CGD	27.52552521	29.03577605	8.13663207
4	43	O1D	28.19490988	28.65947423	7.22612449
4	44	O2D	27.64117526	30.23471192	8.67564502
4	45	CED	28.64799449	31.08566491	8.15928197
4	46	C1	28.95767659	27.10923311	4.13553755
4	47	H	21.66685780	29.81061354	4.02065983
4	48	H	17.72101689	31.70268739	8.58447480
4	49	H	22.13870150	30.40675522	12.97899089
4	50	H	24.10155977	30.69040845	3.51627430
4	51	H	25.63110641	29.82687272	3.60453401
4	52	H	25.12076607	30.85262129	4.93642077
4	53	H	25.21864849	26.23341407	6.68193806
4	54	H	23.74496563	26.44560058	5.78393391
4	55	H	25.14808037	24.98743014	4.51487655
4	56	H	25.17755325	26.51400926	3.64480431
4	57	H	19.84754889	31.36255279	3.09629354
4	58	H	18.13898497	31.03965768	3.33915039
4	59	H	19.27213631	29.71208083	3.20600396
4	60	H	16.97216729	32.32470477	5.08006305
4	61	H	16.10986297	30.41980372	7.29068206

Pigment No	Atom No	Atom Name	Coordinates		
4	62	H	15.06711547	31.56262061	6.31143163
4	63	H	16.65826295	31.47401023	10.90047299
4	64	H	17.33082326	33.08308084	10.74146217
4	65	H	17.21935783	32.33357850	12.31589538
4	66	H	20.19560386	30.75066937	14.05616044
4	67	H	18.62573407	31.44581372	13.84084262
4	68	H	21.24978475	33.00595392	13.64889410
4	69	H	20.13260521	33.03874469	15.00539156
4	70	H	19.65344447	33.69920998	13.44888560
4	71	H	25.85225519	29.09579865	13.37487580
4	72	H	24.24752356	28.53887665	13.80418172
4	73	H	24.59485692	30.25729860	13.76978671
4	74	H	26.59535117	27.18985236	8.51019611
4	75	H	28.59228722	31.99214656	8.74073603
4	76	H	28.46577587	31.29736723	7.11520459
4	77	H	29.62031571	30.62833747	8.26866345
4	78	H	29.44437267	26.20806829	3.79179070
4	79	H	29.22051453	27.30203838	5.16321838
4	80	H	29.23425784	27.94468535	3.51122330
4	81	H	26.01646777	28.46790022	5.71153937
4	82	H	23.73766114	28.28462954	4.03741258

Table S16: Same as in Table S2, but using Hartree-Fock for the geometry optimization. The attachments to this document include the above cartesian atomic coordinates in the file ***table_s16_coordinates.xyz***.

N	Embedding	ω^N (eV)	μ^N (D)	ϑ^N (°)	φ^N (°)
1	none	2.786	5.27	-1.0	-9.5
	impl	2.776	6.06	-1.0	-7.9
	impl+expl	2.776	3.48	-2.1	-5.6
2	none	2.785	5.27	-0.9	-9.5
	impl	2.775	6.06	-1.0	-7.6
	impl+expl	2.775	3.45	-2.9	-4.8
3	none	2.787	5.26	-0.9	-9.4
	impl	2.776	6.06	-1.0	-7.7
	impl+expl	2.776	3.48	-4.0	-6.4
4	none	2.781	5.29	-1.4	-9.8
	impl	2.772	6.06	-1.6	-8.1
	impl+expl	2.772	3.48	-2.3	-5.2

Table S17: Same as in Table 1 of the main text, but using HF-CIS and the geometry from Table S16.

$\boldsymbol{M}$	$\boldsymbol{N}$	$V_{MN}^{ES}(\varepsilon=1)$	$V_{MN}^{ES}(\varepsilon=2)$	$V_{MN}^{ES}(\varepsilon=2)+V_{MN}^{solv}(\varepsilon=2)$	f_{MN}^{rf}	s_{MN}
3	4	156	203	125	1.299	0.619
1	2	155	202	126	1.303	0.622
2	3	25	33	20	1.302	0.604
1	4	25	33	20	1.300	0.604
2	4	9	12	9	1.444	0.693
1	3	7	10	7	1.448	0.714

Table S18: Same as in Table 2 of the main text, but using HF-CIS and the geometry from Table S16.

$\boldsymbol{M}$	$\boldsymbol{N}$	$\varepsilon_s=2$	$\varepsilon_s=4$	$\varepsilon_s=10$	$\varepsilon_s=20$
3	4	91	92	93	93
1	2	89	90	91	91
2	3	21	21	21	20
1	4	20	19	19	19
2	4	7	7	7	7
1	3	6	6	6	6

Table S19: Calibrated excitonic couplings (in units of 1/cm) between 0-0 transitions of Chl *a* pigments in WSCP, obtained by using different values for the static dielectric constant ε_s . The original data are given in Tables S6 and S7 ($\varepsilon_s=2$), Tables S8 and S9 ($\varepsilon_s=10$), and Tables S10 and S11 ($\varepsilon_s=2$). The values of the 4th column ($\varepsilon_s=4$) are identical with that of the 3rd column in Table 8 of the main text.

Comparison of ESP of TrEsp Charges fitted with and without solvent

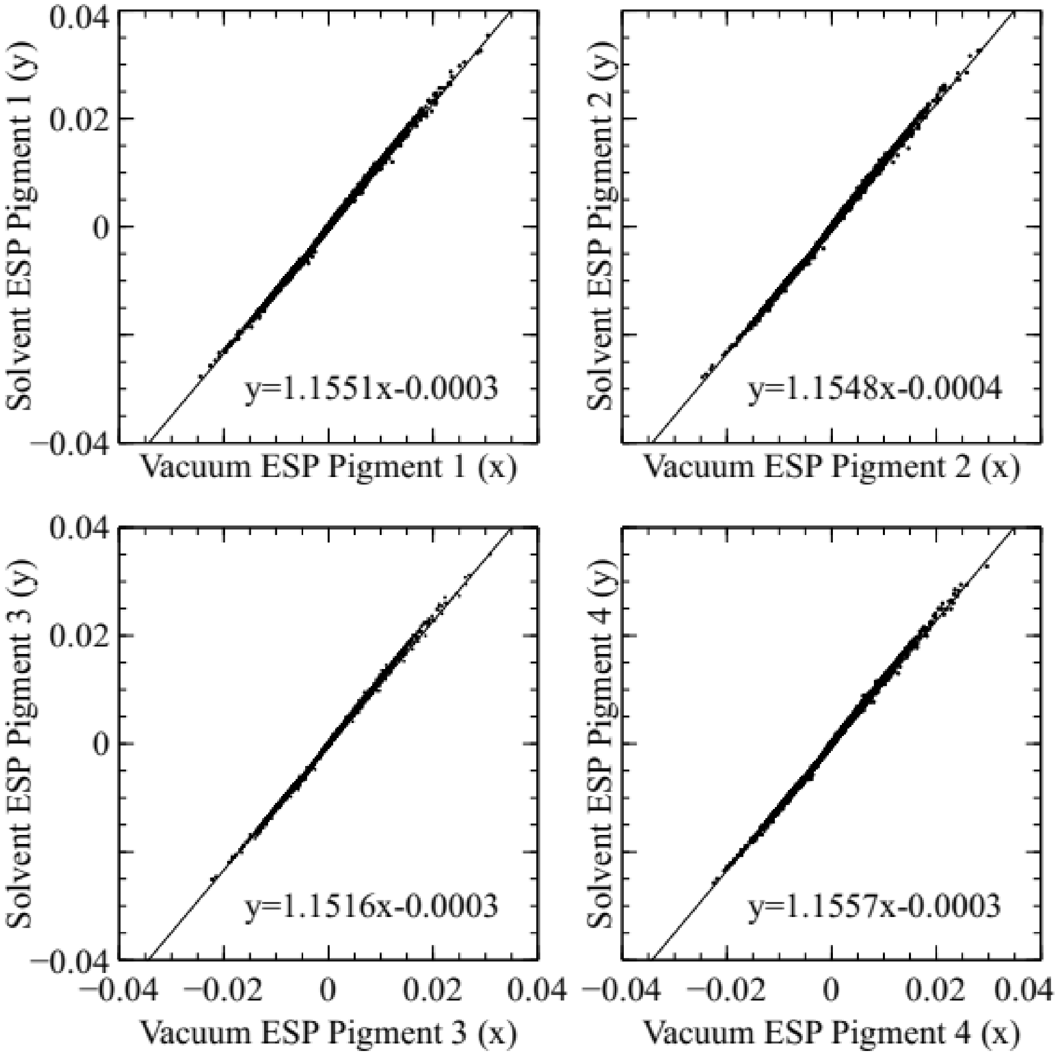

Figure S3: Electrostatic potential from the transition density at the evaluation points used for fitting the charges in Table S2 compared between vacuum and solvent environment for each individual pigment. The thin black lines are result from individual linear regressions.

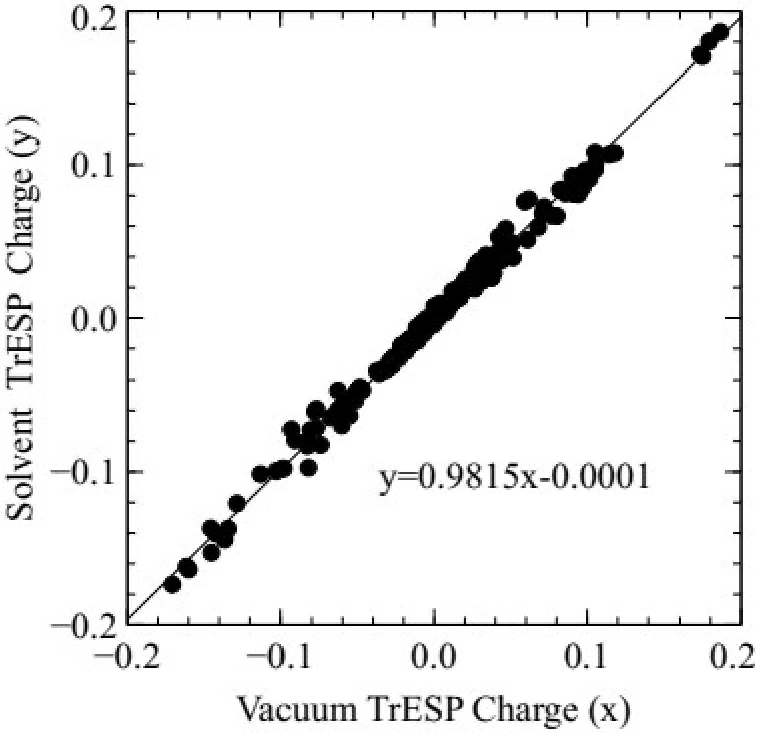

Figure S4: Vacuum TrESP Charges vs. Solvent TrESP Charges from Table S2, all pigments. The thin black line is the result of a linear regression. Although the transition dipole moments of the TrESP charges changes by a factor of 1.2 (see Table 1 of the main text), there is no consistent scaling o the TrESP charges. This is because the charges are not uniquely determined by the ESP, that is fitted by a least squares procedure.

Dipole strengths at varying refractive indices

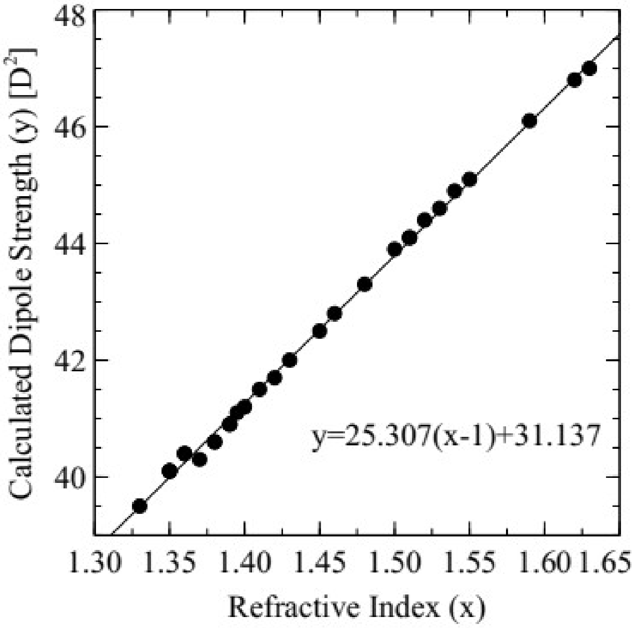

Figure S5: Dependence of dipole strength of pigment 1, calculated with TDDFT/PCM with the CAM-B3LYP XC-funcctional, on the refractive index (coordinates taken from Table S2). The inset shows the result of a linear regression.

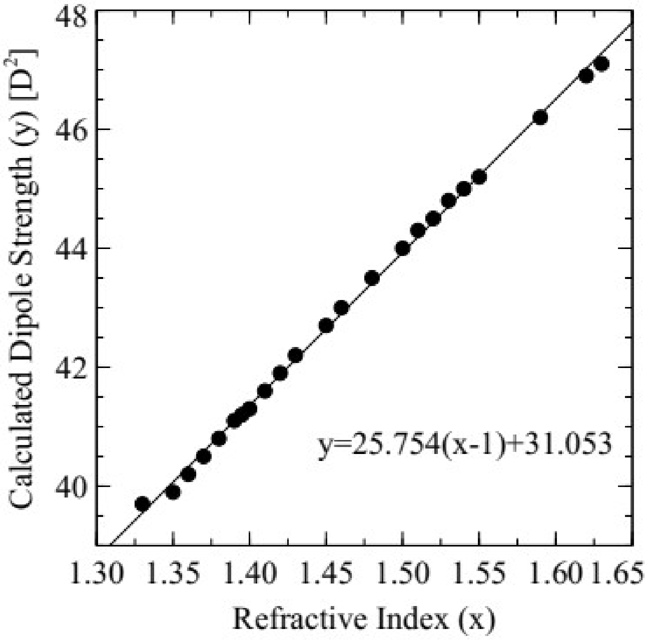

Figure S6: Same as Figure S5 but for Pigment 2.

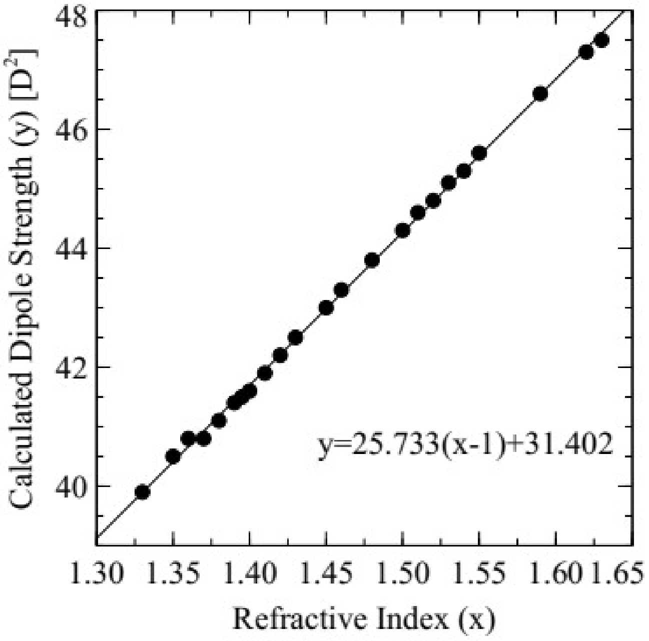

Figure S7: Same as Figure S5 but for Pigment 3.

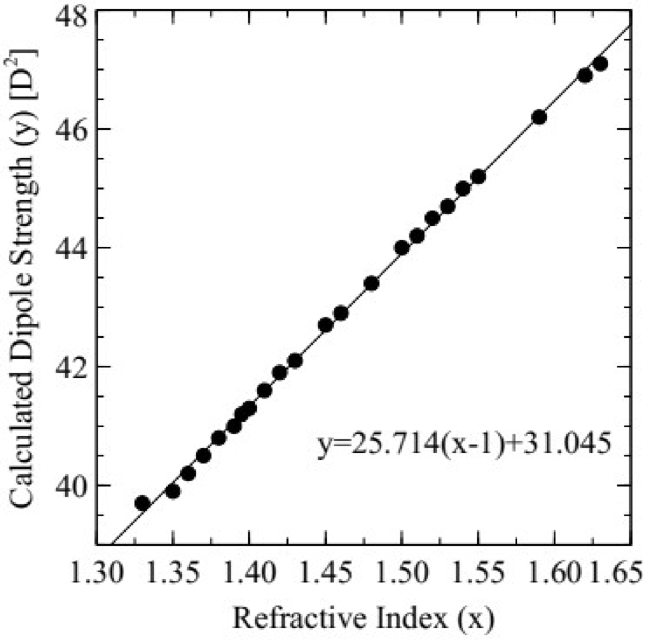

Figure S8: Same as Figure S5 but for Pigment 4.

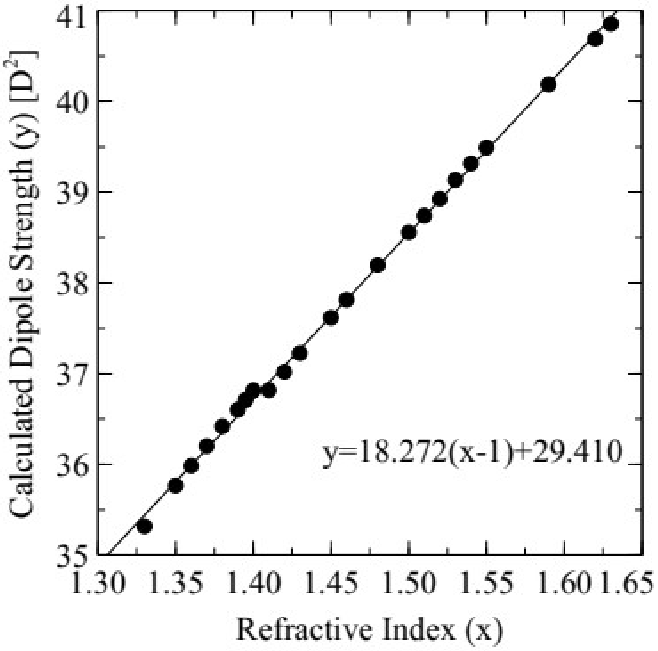

Figure S9: Same as Figure S5 but using HF/CIS calculations.

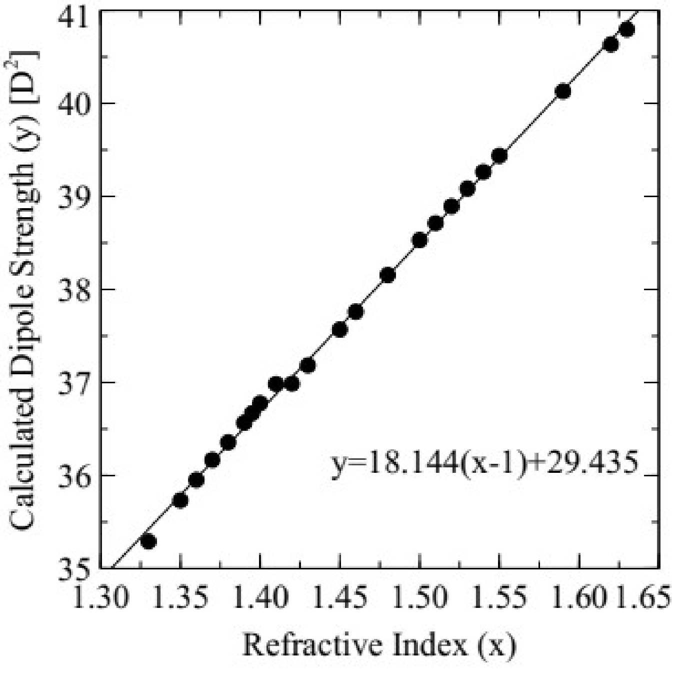

Figure S10: Same as Figure S9 but for pigment 2.

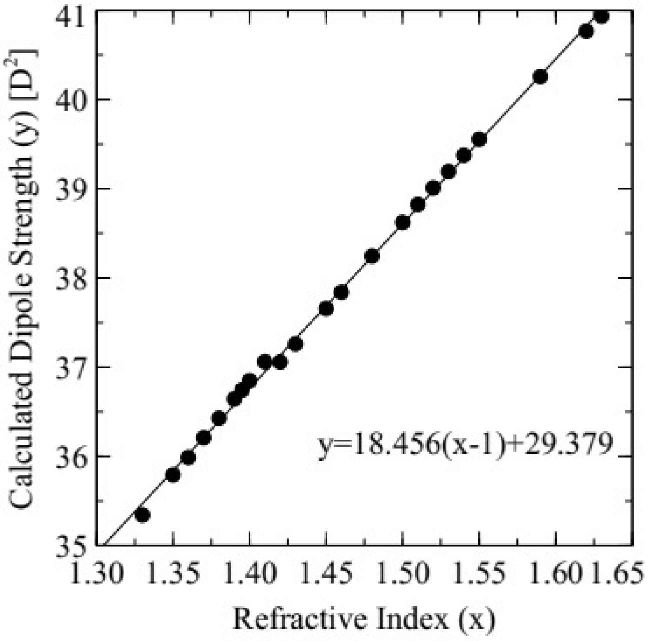

Figure S11: Same as Figure S9 but for Pigment 3.

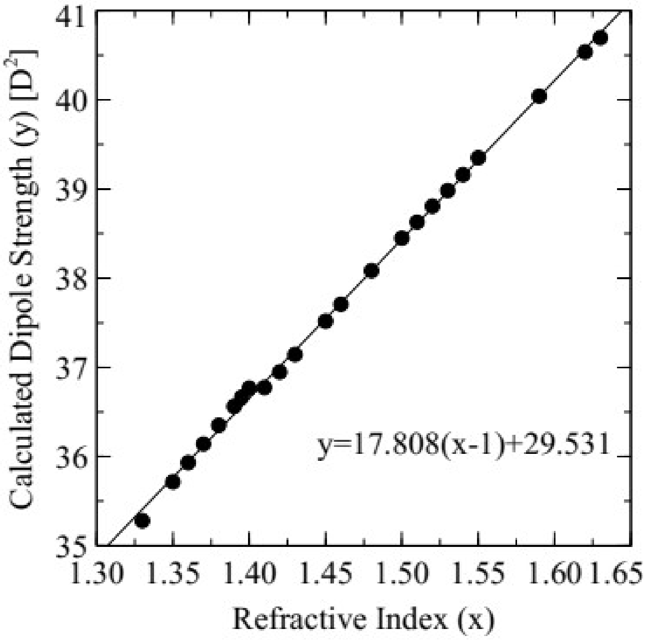

Figure S12: Same as Figure S9 but for Pigment 4.
